# Renal sodium handling and blood pressure changes in gestational protein-restricted offspring: Role of renal nerves and ganglia neurokinin expression

**DOI:** 10.1371/journal.pone.0179499

**Published:** 2017-06-20

**Authors:** Augusto H. Custódio, Marcelo C. de Lima, Bárbara Vaccari, Patrícia A. Boer, José A. R. Gontijo

**Affiliations:** Department of Internal Medicine Faculty of Medical Science, State University of Campinas, Campinas, SP, Brazil; University Medical Center Utrecht, NETHERLANDS

## Abstract

**Background:**

Considering long-term changes in renal sodium handling and blood pressure in maternal protein-restricted (LP) offspring, we assumed that the development of LP hypertension results from abnormal dorsal root ganglia (DRG) neurokinin expression associated with impaired responsiveness of renal sensory receptors, promoting a reduced urinary excretion of sodium. The present study investigates whether increased blood pressure in protein-restricted offspring would be associated with changes in the DRG cells and in renal pelvic wall expression of NK1R, SP and CGRP when compared to NP offspring. In addition, we assessed the tubular sodium handling, estimated by creatinine and lithium clearances before and after bilateral renal denervation in conscious LP offspring relative to age-matched NP counterparts.

**Methods:**

Dams received a normal (NP) or low-protein diet (LP) during their entire pregnancy period. Male NP or LP offspring underwent bilateral surgical renal denervation before the 8-week renal functional test and blood pressure measurements. Immunofluorescence staining in DRG cells was assessed in optical sections by confocal laser scanning microscope.

**Results:**

The current data demonstrated a sustained rise in blood pressure associated with a decrease in fractional excretion of sodium (FENa) by reducing post-proximal tubule sodium rejection in 16-wk old LP rats relative to age-matched NP counterparts. According to this study, bilateral renal denervation attenuated blood pressure and increased FENa in LP offspring. Furthermore, an immunohistochemical analysis showed a reduced expression of SP and CGRP in DRGs of LP when compared with NP rats. Renal pelvis of LP rats did not show a strong CGRP expression related to NP rats, whereas there was no change in SP immunostaining.

**Conclusions:**

These observations raise the possibility that impaired DRG and pelvic neurokinin expression associated with responsiveness of renal sensory receptors in 16-wk old LP offspring are conducive to excess renal reabsorption of sodium and development of hypertension in this programmed model.

## Introduction

Disruptions in fetal programming result in low birthweight, fewer nephrons, and increase the risk of cardiovascular and renal disorders in adults [1–5]. We recently demonstrated that gestational low-protein (LP) intake offspring have a lower birthweight, arterial hypertension, and 30% fewer nephrons [3,4] when compared to normal (NP) protein intake group. In addition, hydroelectrolytic balance studies have shown that arterial hypertension in gestational protein-restricted offspring is associated with decreased renal salt and water excretion when compared with pair-fed, age-matched normal protein intake (NP) rats [3–7].

Previous studies have demonstrated that efferent renal nerve activity (ERNA) is enhanced in many models of hypertension and may be related to water and salt retention, highlighting that both of them contribute to hypertension [8–10]. Additionally, studies in rats have presented that the activation of renal mechanoreceptors (MR) or chemoreceptors (CR) increases ipsilateral afferent renal nerve activity (ARNA) being associated with a decrease in contralateral ERNA and an increased natriuresis. These findings indicate a contralateral inhibitory renorenal reflex response [11–13]. In this way, data from genetically hypertensive rats has demonstrated that increased renal pelvic pressure failed to enhance afferent renal nerve activity. The inhibitory nature of renorenal reflexes indicates that impaired renorenal reflexes could contribute to increased sodium retention and hypertension in this lineage [14,15].

The renal sensorial afferent neurons are predominantly unmyelinated and project to the T10–L3 ipsilateral dorsal root ganglia (DRG) from sensory receptors located in the renal veins, arteries, and renal pelvic wall [11,16–20]. Calcitonin gene-related peptide (CGRP) and substance P (SP) colocalize in these sensory neurons of the renal pelvic wall [21,22]. Furthermore, CGRP regulates the expression of Neurokinin-1 (NK1) receptors in rat spinal neurons [23] and retards the metabolism of SP [12], thereby increasing the amount of SP available for SP receptor stimulation. Three distinct tachykinin receptors, NK_1_, NK_2_, and NK_3_, specific G-protein-coupled membrane receptors, have now been cloned in different species [24,25]. NK_1_R are widely distributed in the renal pelvis and brain, and have been implicated in nociceptive signaling to the spinal cord [26,27].

In the current study, we assumed that LP hypertension development might result, at least in part, from abnormal DRG expression of neurokinins associated with impaired responsiveness of renal sensory receptors, consequently promoting a decreased segmental tubule sodium excretion. To test this hypothesis, the present study was therefore undertaken to investigate whether increased blood pressure in protein-restricted offspring would be associated with changes in the DRG (T13) cells and in renal pelvic wall expression and localization of NK1R, SP and CGRP when compared to NP offspring. In addition, we assess the glomerular filtration rate and tubular sodium handling, estimated by creatinine and lithium clearances in conscious 16-week old LP offspring, before and after the bilateral renal denervation relative to age-matched NP counterparts.

## Material and methods

### Animals

The experiments were conducted on age-matched offspring of sibling-mated Wistar HanUnib rats (250–300g). The environment and housing presented good conditions for managing their health and well-being during the experimental procedure. The study design was approved by the State University of Campinas Institutional Animal Ethics Committee *(protocol CEUA/UNICAMP #2575–1)* and conformed to general guidelines established by the Brazilian College of Animal Experimentation (COBEA). Our local colonies originated from a breeding stock supplied by CEMIB/Unicamp, Campinas, SP, Brazil. Immediately after weaning at 3 weeks of age, animals were maintained under controlled conditions of 25°C and a 12-h light–dark cycle, with free access to tap water and standard rodent laboratory chow (Nuvital, Curitiba, PR, Brazil). We designated as day 1 of pregnancy the day in which the vaginal smear presented sperm. Pregnant dams were fed isocaloric standard rodent laboratory chow (Na content: 135 ± 3 μEq/g; K content: 293 ± 5 μEq/g), with normal 17% protein content (NP) or low 6% protein (LP) content *ad libitum* throughout the entire pregnancy with free access to tap water. All groups returned to the NP diet intake after delivery. The offspring birth weight was measured. The pups weaned in 3 weeks and only one male offspring of each litter was used for each experiment. The male offspring were maintained under a controlled temperature (25°C) and lighting conditions (07:00 am–07:00 pm) and, followed up to 16 weeks of age. Food consumption was monitored daily and normalized to the body weight. Body weight was recorded weekly.

### Surgical procedures

For renal bilaterally denervated offspring [NP_DNx_ (n = 9) and LP_DNx_ (n = 8)], surgery was performed before the 8-week renal functional test and blood pressure measurements. Briefly, the animals were anesthetized with a mixture of ketamine [75 mg/kg^−1^ body weight, injected intraperitoneally (i.p.)] and xyla**z**ine (10 mg/kg^−1^ body weight, i.p.). Once the corneal and pedal reflexes were absent, both kidneys were exposed by dorsal abdominal incisions and surgically denervated with the aid of a stereomicroscope. Denervation was performed by cutting all visible nerves along the renal artery and by stripping the connective tissue passing by and along the course of the renal artery and vein. Immediately after, the renal vessels were wrapped and surrounded with cotton swabs soaked in 10% (v/v) phenol diluted in absolute ethanol [14,28]. Sham-operated rats underwent all surgical procedures but the renal artery was left intact. Postoperative analgesia was performed for 1-day with acetaminophen (40 mg/100g body weight) administrated intraperitoneally, and observed them individual and daily in metabolic cages under controlled environmental conditions. Rats were used for experiments 1 week after renal denervation.

### Blood pressure measurement

The systolic arterial pressure was measured in conscious and previously trained offspring at 6, 8, 10, 12, 14, and 16 weeks of age (NP n = 9, NP_DNx_ n = 9, LP and LP_DNx_ n = 8, and LP n = 8). Blood pressure was measured using an indirect tail-cuff method with an electrosphygmomanometer (IITC Life Science***—***BpMonWin Monitor Version 1.33) combined with a pneumatic pulse transducer/amplifier. This indirect approach allowed repeated measurements with close correlation (correlation coefficient = 0.975) compared with direct intra-arterial recording. The mean of three consecutive readings was taken as the blood pressure.

### Renal function evaluation

Renal function was measured by creatinine and lithium clearance at 10 weeks of age (NP n = 9, NP_DNx_ n = 9, LP and LP_DNx_ n = 8), and 16 weeks of age (LP n = 8, NP n = 8, NP_DNx_ n = 8, LP and LP_DNx_ n = 8) unanaesthetized, unrestrained male offspring. Briefly, fourteen hours before the renal test, 60-μmol LiCl.100^-1^g body weight was given by means of gavage. Subsequently, we housed the rats in metabolic cages with free access to tap water but no food. After an overnight fast, each animal received tap water by gavage (5% of body weight), followed by a second load of the same volume 1-h later. Spontaneously voided urine was collected over a 120-min period into a centrifuge tube and measured gravimetrically. Plasma and urine sodium, potassium and lithium concentrations were measured by flame photometry (Micronal, B262, São Paulo, Brazil), while the creatinine concentrations and plasma osmolality were determined spectrophotometrically (Instruments Laboratory, Genesys V, USA) and by Wide-range Osmometer (Advanced Inst. Inc, MA, USA), respectively. Thereafter, animals were anaesthetized with ketamine and xylazine injected intraperitoneally and sacrificed by cardiac puncture; urine and plasma samples were stored for analysis [3,4,6,7,29,30].

### Immunofluorescence detection of NK1, SP, and CGRP

Sixteen-week-old male NP (n = 15), and LP (n = 15) rats were used for immunofluorescence experiments. Rats were anesthetized and perfused with saline containing 2% heparin via the left carotid artery for 5 min under constant pressure. This was followed by perfusion with 0.1 M phosphate buffer (pH 7.4) containing 4% (w/v) paraformaldehyde and 0.1 M sucrose. After perfusion, the left T13 DRG and kidneys were immediately removed and placed in the same fixative for 1 h, followed by phosphate-buffered saline (PBS) containing 0.1% glycine for 1 h and PBS containing 15% (w/v) sucrose overnight. Then, tissues were immersed in OCT cryoprotector (Tissue-tech) and frozen in liquid nitrogen (−79°C). Sections (7 μm thick) were cut at −22°C using a Leica cryostat and collected on saline-coated slides. For immunohistochemistry, sections were blocked in PBS containing 3% normal donkey serum and 3% bovine serum albumin for 45 min to minimize nonspecific reactions. After blocking, sections were labeled with rabbit anti-NK1R antiserum (1:100 dilution; Novus Biologicals®), rabbit anti-CGRP antiserum (1:100 dilution; Neuromics®), or goat anti-SP antiserum (1:600; Santa Cruz), at 4°C overnight. Then, sections were incubated in anti-rabbit DyLight® 594-labeled secondary antibody (1:200 dilution) or rabbit anti-goat Cy™ 3-labeled secondary antibody (1:200 dilution) for 2 hours at room temperature. Then, sections were rinsed in 0.1 M PBS and cover-slipped with Vectashield anti-fading medium containing DAPI (Vector). The sections were examined with a confocal laser scanning microscope (LSM 780***—***ZEISS) and digital images were taken using fixed settings and specific software (LSM; Zeiss). The present study take in account the protocol for quantitation of fluorescence intensity from Waters (2007) [31]. The fluorescence microscopy digital image background was subtracted from intensity value measurements to reveal the real signal. To avoid errors due to an inhomogeneous background, we measured pixels immediately adjacent to object of interest [32]. This is especially important in the current study making measurements of intracellular structures once the background in the cytoplasm is different from the background outside of cells, and is usually inhomogeneous. No immunoreactivity was observed when the primary antibodies were omitted.

### Cellular population analysis

Immunofluorescence staining in DRG cells was assessed in optical sections by confocal laser scanning microscope (CLSM). Using this approach, immunostaining was easily recognized. To determine the cell size, five random DRG sections immunostained with NK_1_R, SP, and CGRP antisera were selected from fifteen different T13 DRG groups. Images were analyzed using Image J software. Briefly, the boundaries of immunostained cells and nuclei profiles were traced manually using a computer mouse and the intensity of fluorescence and cell areas were automatically calculated. To compare the current quantitative results with previous DRGs analysis, the cell areas were transformed into cell diameters by assuming that dorsal ganglion neurons are circular and, that neuron subpopulations diameter intervals were 10–25, 25–37.5 and 37.5–60 μm respectively, to small, intermediate and large cells [33,34]. All quantified sections were optimally stained that same day.

### Catecholamine assay

Plasma catecholamines were extracted from the medium using Al_2_O_5_ (alumina) and HBA (dihydroxybenzylamine) as internal standard, and quantified by ion-pair reverse phase chromatography coupled with electrochemical detection (0.5 V) as described by Di Marco et al. [35]. Kidney norepinephrine contents were assessed by the Anton and Sayre method [36].

### Data presentation and statistical analysis

Results were expressed as mean ± standard deviation (SD), scatter dot plots, or median and quartile deviation as appropriate. Creatinine clearance (C_Cr_) was used to estimate glomerular filtration rate (GFR) and lithium clearance (C_Li_) was used to assess proximal tubule output. Fractional sodium excretion (FE_Na_) was calculated as C_Na_/C_Cr_×100, where C_Na_ is sodium clearance. Fractional proximal (FEP_Na_) and post-proximal (FEPP_Na_) sodium excretion were calculated as C_Li_/C_Cr_ × 100 and C_Na_/C_Li_ × 100, respectively [3,4,6,7,29,30]. Data obtained over time were analyzed using two-way ANOVA or nonparametric analysis using the Kruskal–Wallis test. When statistically significant differences were indicated between selected means by ANOVA, *post hoc* comparisons were performed with Bonferroni’s contrast test. Comparisons involving only two means within or between groups were carried out using a Student’s t*-*test. The level of significance was set at P ≤ 0.05.

## Results

**Fig 1:**
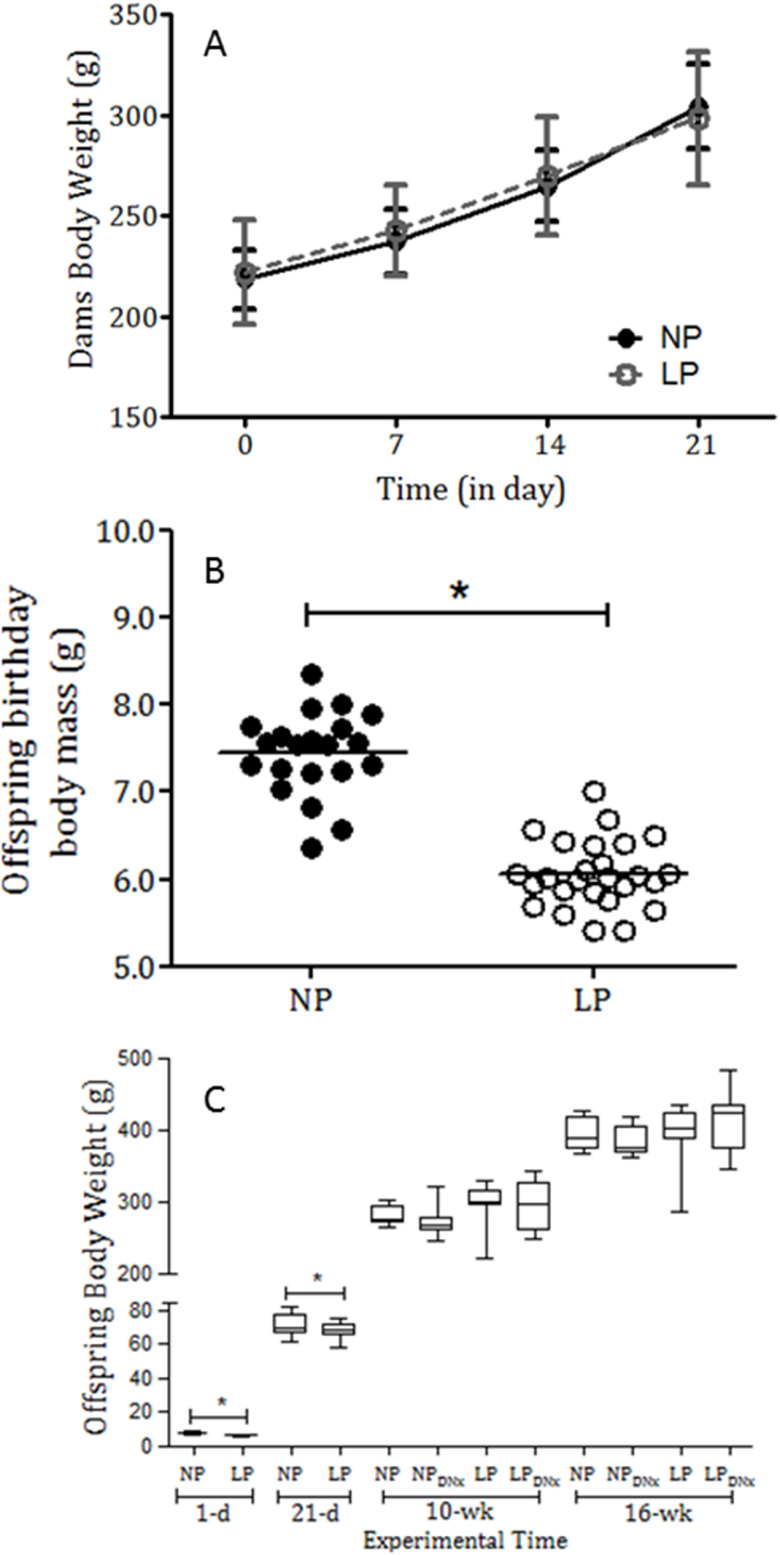
Dams’ body weights (A), offspring body weight at birth (B) and (C) 1-day, 21-day, 10-week, and 16-week-old NP and LP body weight (in grams) compared with bilateral renal denervated NP and LP offspring. The results are expressed as means ± SD, means ± scatter dot plot, and median and quartile deviation. Data were analyzed using nonparametric analysis by Kruskal–Wallis or Student´s t-test and two-way ANOVA test with *post hoc* comparisons by Bonferroni’s contrast test. The level of significance was set at *P < 0.05.

Table 1 shows serum sodium, lithium, and potassium levels from NP and LP sham and denervated offspring, with no significant differences in NP rats compared with the LP group. In general, water and food sodium intake and, plasma osmolality were similar in male offspring of NP and LP groups when normalized by body weight (Table 1). Maternal protein restriction during pregnancy did not significantly change the body mass of pregnant dams during gestation (Fig 1A). In addition, it did not affect the number of offspring per litter and the proportion of male and female offspring (P = 0.3245). The birthweight of LP male pups (n = 26) was significantly reduced compared with NP pups (n = 21) (6.06 ± 0.075 g *vs*. 7.44 ± 0.10 g; P = 0.001) (Table 1 and Fig 1B). The body mass of LP pups (67.9 ± 0.99 g, n = 17) remained lower than age-matched NP pups (72.10 ± 1.42 g, n = 20) until weaning at 21 days after birth (P = 0.05) (Fig 1C). However, after 10 weeks of age, the body weight of LP and NP rats was not significantly different. Bilateral renal denervation did not affect body mass in any experimental group (Fig 1C).

**Table 1:** Serum sodium, lithium, and potassium levels and sodium and water intake and plasma osmolality in maternal normal protein intake (NP) offspring and maternal low-protein intake (LP) offspring compared with bilateral renal denervated NP (NP_DNx_) and LP (LP_DNx_) rats (n = 10 animals for each group). The data represent the means ± SEM. The level of significance was set at *P ≤ 0.05 (one-way ANOVA or Student’s t-test).

*Groups/Parameters*	*Na*^*+*^ *(mM)*	*Li*^*+*^ *(μM)*	*K*^*+*^ *(mM)*	*Na*^*+*^*Intake (mmol*.*wk*^*-1*^.*100g*^*-1*^ *bw)*	*H*_*2*_*O Intake (ml*.*100g*^*-1*^*bw)*	*Plasma Osmolality* *(mOsm*.*kg*^*-1*^*H*_*2*_*O)*
***NP (n = 10)***	138 ± 3.6	85 ± 21	4.3 ±0.6	13.6 ± 2.4	22.5 ± 7.6	293 ± 8.0
***LP (n = 10)***	142 ± 4.1	78 ± 20	4.1± 0.5	12.7 ± 2.1	23.7 ± 5.2	297 ± 7.0
***NP***_***DNx***_ ***(n = 10)***	140 ± 2.5	91 ± 23	4.1 ±0.6	13.2 ± 2.6	26.8 ± 7.4	295 ± 7.0
***LP***_***DNx***_ ***(n = 10)***	141 ± 3.7	87 ± 24	3.9 ±0.7	11.9 ± 4.9	25.7 ± 6.2	293 ± 8.0

### Blood pressure measurement

**Fig 2:**
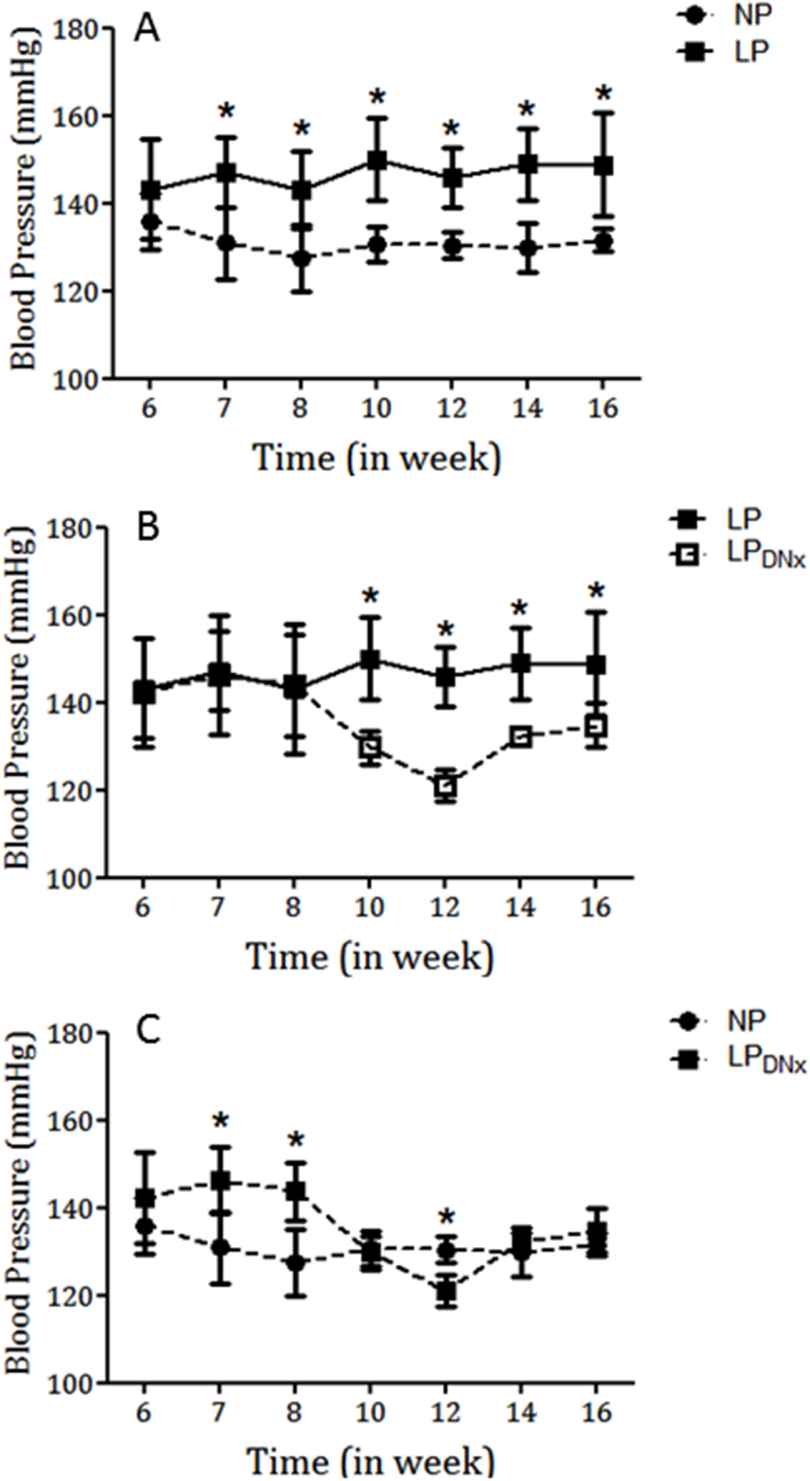
Graphic representation of the systolic blood pressure (mmHg) time-course measured in conscious male NP (n = 8) and LP (n = 8) offspring (A) compared with gender and age-matched bilateral renal-denervated LP (n = 8) offspring (panel B *vs*. LP, n = 8) and (panel C *vs*. NP, n = 8) offspring. Values are means ± SD. *P < 0.05; (one-way ANOVA; *post hoc* Bonferroni’s contrast test).

As shown in Fig 2A, tail systolic arterial pressure (mmHg) was significantly higher in LP offspring compared with NP offspring between 7 weeks and 16 weeks of age. The changes in systolic blood pressure from 7 weeks to 16 weeks of age were as follows: 7 weeks: LP, 147 ± 9.0 mmHg *vs*. NP, 131 ± 8.0 mmHg, P = 0.001; 16 weeks: LP, 149 ± 12 mmHg *vs*. NP, 131 ± 2.0 mmHg, P = 0.001 (Fig 2A). Fig 2 also shows the effect of bilateral renal denervation on blood pressure at 8 weeks of age. The continuous increase in blood pressure in LP offspring was significantly reduced by bilateral renal denervation (Fig 2B) over a 10-week period (between 6 weeks and 16 weeks of age). Renal bilateral phenol denervation significantly prevented arterial pressure increase for up to 8 weeks compared with the non-denervated LP group (P = 0.001). In addition, there was no significant difference in blood pressure between the LP renal bilaterally denervated rats and the NP group (Fig 2C). This attenuation in blood pressure was associated with a significant increase in urinary sodium excretion and a decrease in proximal sodium reabsorption, as described below.

### Renal function evaluation

**Fig 3:**
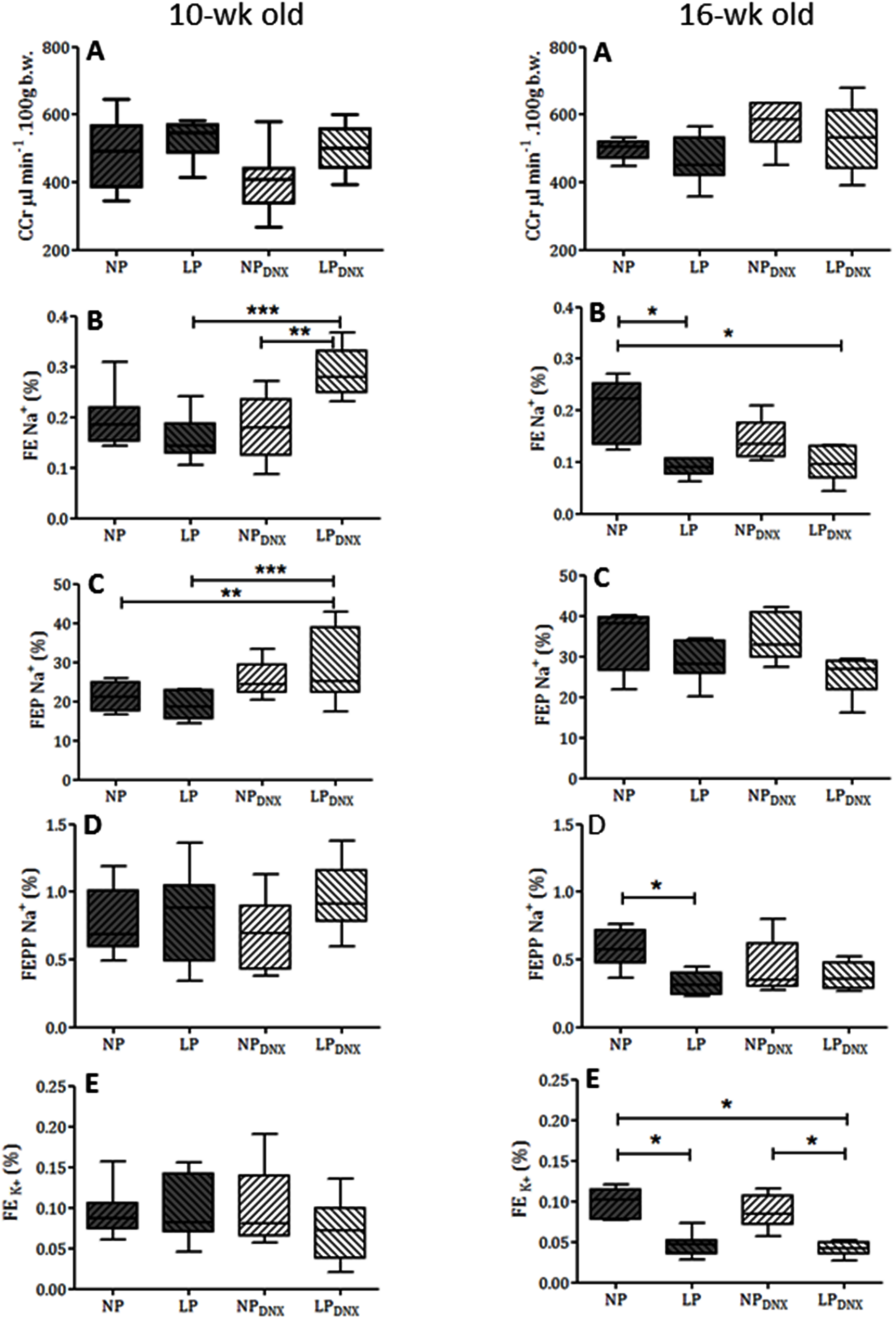
Renal function studies using creatinine clearance (CCr, panel A), fractional sodium excretion (FENa+, panel B), proximal (FEPNa+, panel C) and post-proximal (FEPPNa+, panel D), fractional sodium excretion and fractional potassium excretion (FEK+, panel E), in male 10-week-old and 16-week-old NP and LP offspring compared with age-matched NP_DNx_ and LP_DNx_ groups (n = 10 animals for each group). Results are expressed as median and quartile deviation. Data were analyzed using nonparametric analysis by Kruskal–Wallis test with *post hoc* Bonferroni’s contrast test. The level of significance was set at *P ≤ 0.05, **P ≤ 0.01 or **P ≤ 0.001.

Renal function in 10- and 16-week-old NP and LP offspring is summarized in Fig 3. The urinary flow rates (data not included) and the GFR, estimated by C_Cr_, did not significantly differ between the groups even after bilateral renal denervation. Fractional urinary sodium excretion (FE_Na_, Fig 3B) in 10-week old LP rats was unchanged compared with age-matched NP rats (LP: 0.1562 ± 0.041% *vs*. NP: 0.1935 ± 0.051%; P = 0.1347). However, renal denervation in 10-week old LP_DNx_ rats transiently enhanced fractional urinary sodium excretion compared with LP non-denervated and NP_DNx_ rats. The enhanced FE_Na_ in renal-denervated LP rats (0.2894 ± 0,047%, P = 0.0001) was accompanied by a significant increase in proximal sodium excretion (LP_DNx_: 28.15 ± 3.38% *vs*. LP: 19.11 ± 1.27%; P = 0.0127), while FEPP_Na_ and FE_K_ were unchanged compared with age-matched sham-operated offspring. Like 10-week-old rats, 16-week-old NP and LP urinary flow rates (data not included) and GFRs estimated by CCr did not significantly differ among the all studied groups. At this age, fractional urinary sodium excretion was significantly lower in 16-week-old LP rats compared with age-matched NP offspring (16-week-old LP: 0.089 ± 0.006% *vs*. NP: 0.199 ± 0.028%; P = 0.006). The decreased FE_Na_ in LP rats was accompanied by a significant reduction in FEPP_Na_ (16-week-old LP: 0.324 ± 0.031% *vs*. NP: 0.589 ± 0.07%; P = 0.0013) and FE_K_ (16-week-old LP: 0.046 ± 0.006% *vs*. NP: 0.0973 ± 0.008%; P = 0.0002), compared with age-matched NP control rats. At 16 weeks of age, the FE_Na_ increase in LP offspring caused by renal denervation was significantly attenuated and sodium excretion levels were like non-denervated NP and LP rats (Fig 3B). Likewise, increased proximal sodium excretion in renal denervated LP rats at 10 weeks of age was not detected at 16 weeks of age.

### NK_1_R, SP, and CGRP immunostaining in DRG cells

**Fig 4:**
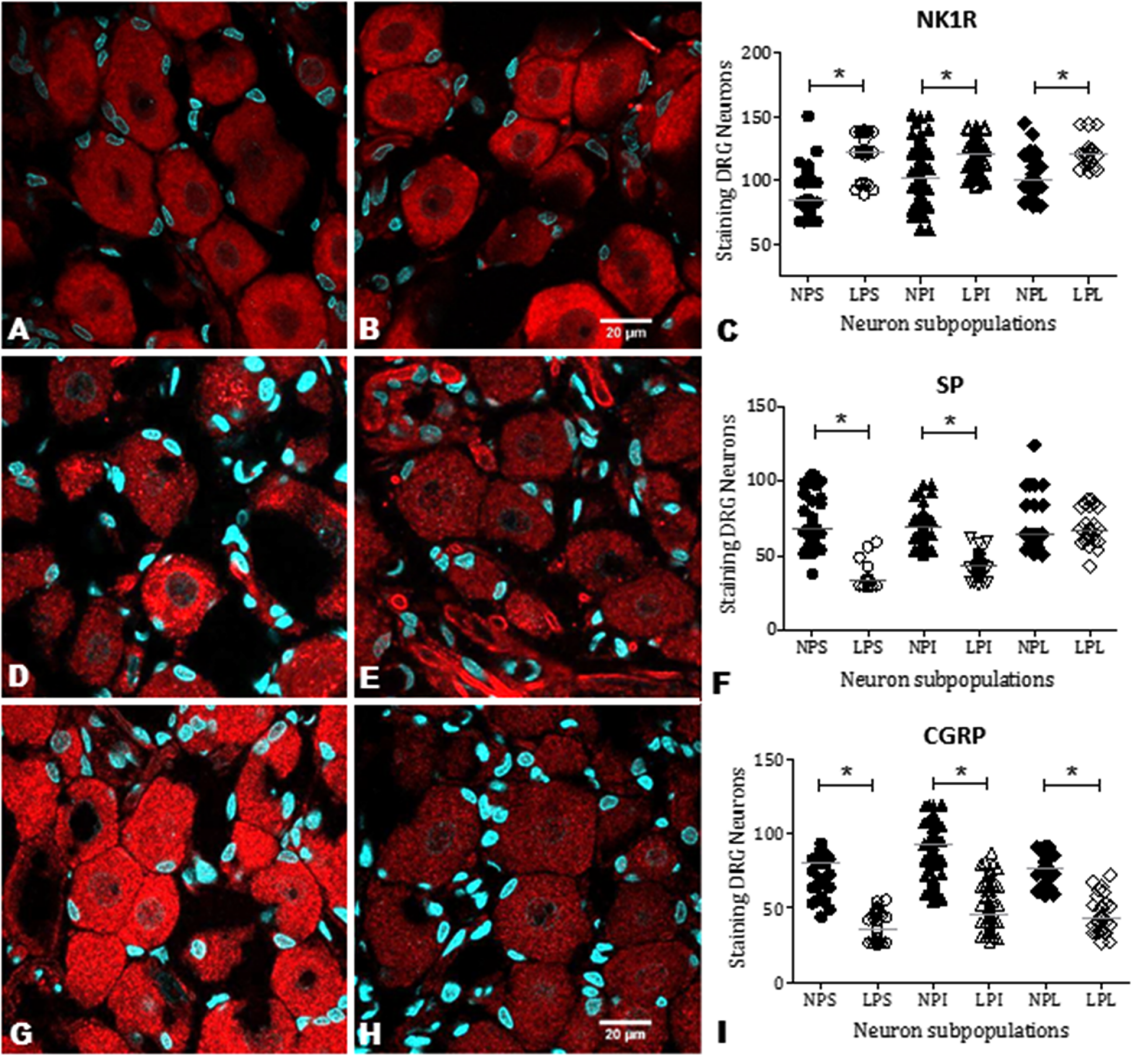
CLSM images and graphics showing the immunoreactivity for NK_1_R, SP, and CGRP in T13 DRG cells. The images show small (S), intermediate (I) and large (L) neurons surrounded by satellite cells. The receptor and neurokinins are present in both nuclear and cytosolic compartments. NK_1_R immunoreactivity was enhanced in LP (B) compared with NP (A) as shown by the histogram of quantification (C). Conversely, SP and CGRP expression was reduced in LP (E and H, respectively) compared with NP (D and G) rats. The statistical significance can be viewed in F and I. The data are reported as the means ± scatter dot plot; n = 15 animals for each group; *P ≤ 0.05 vs. NP offspring (Student’s t-test).

**Fig 5:**
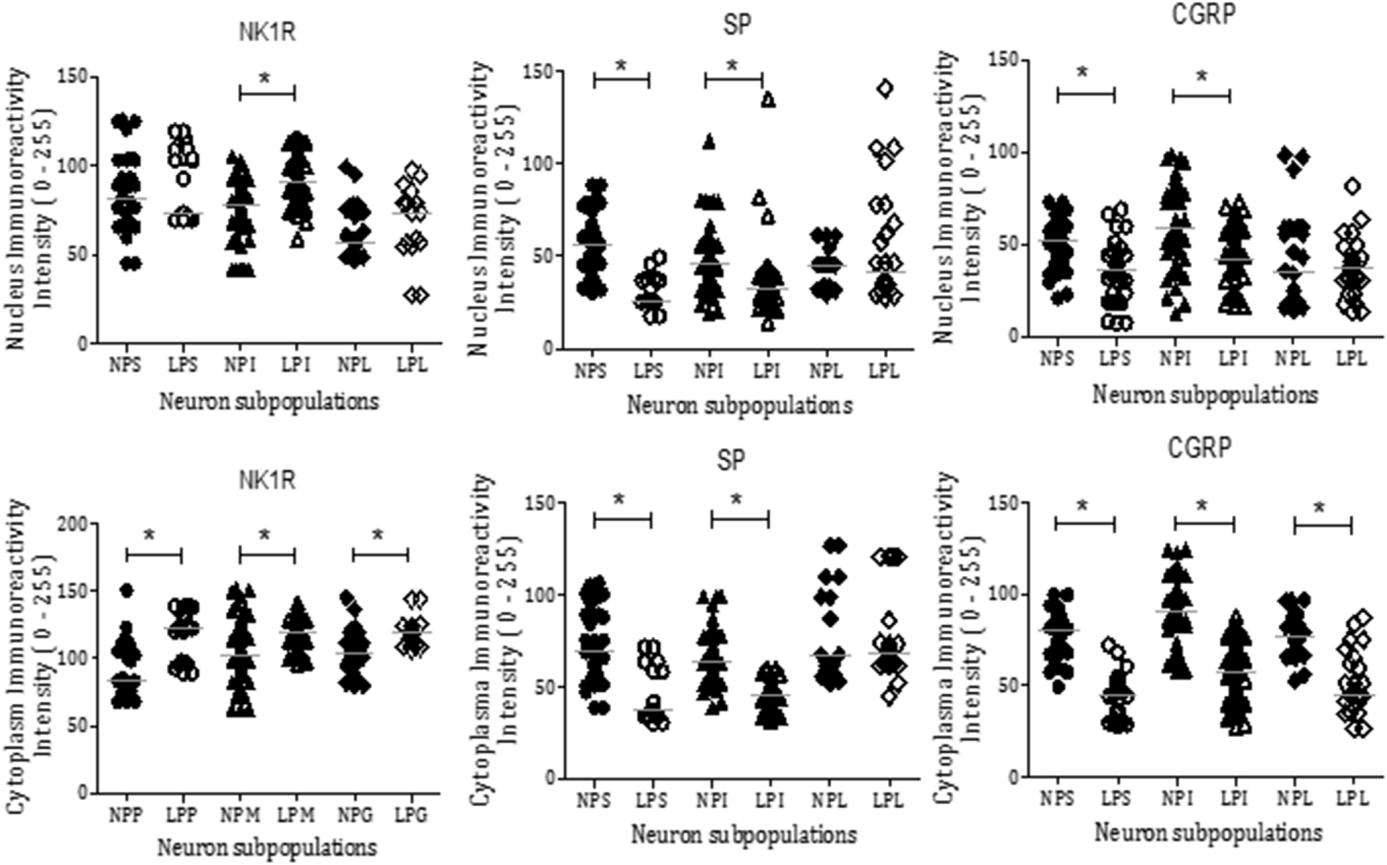
Comparative statistical analysis of NK_1_R, SP, and CGRP expression in the nucleus and cytoplasm. The graphical representation depicts immunoreactivity in the cytosol and nucleus from small (S), intermediate (I) and large (L) neurons of T13 DRG from LP and NP rats. The data are reported as the means ± scatter dot plot; n = 15 animals for each group; *P ≤ 0.05 vs. NP offspring (Student’s t-test).

**Fig 6:**
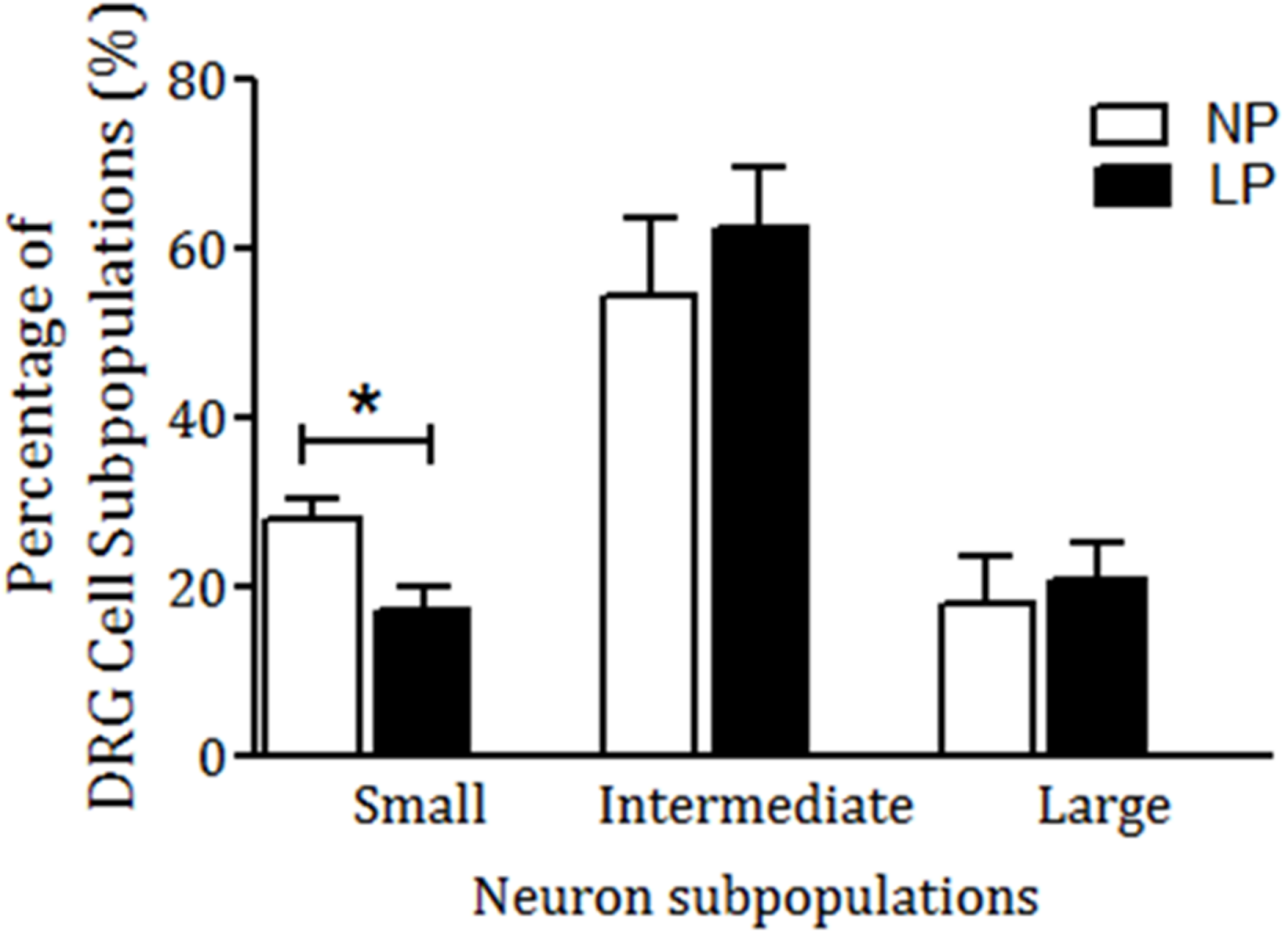
Percentage of DRG cell subpopulations: small (S), intermediate (I) and large (L) neurons expressing NK_1_R, SP, and CGRP in serial 7 μm thick ganglia sections. The data are reported as means ± SD. *P ≤ 0.05 vs. NP offspring (Student’s t-test).

**Fig 7:**
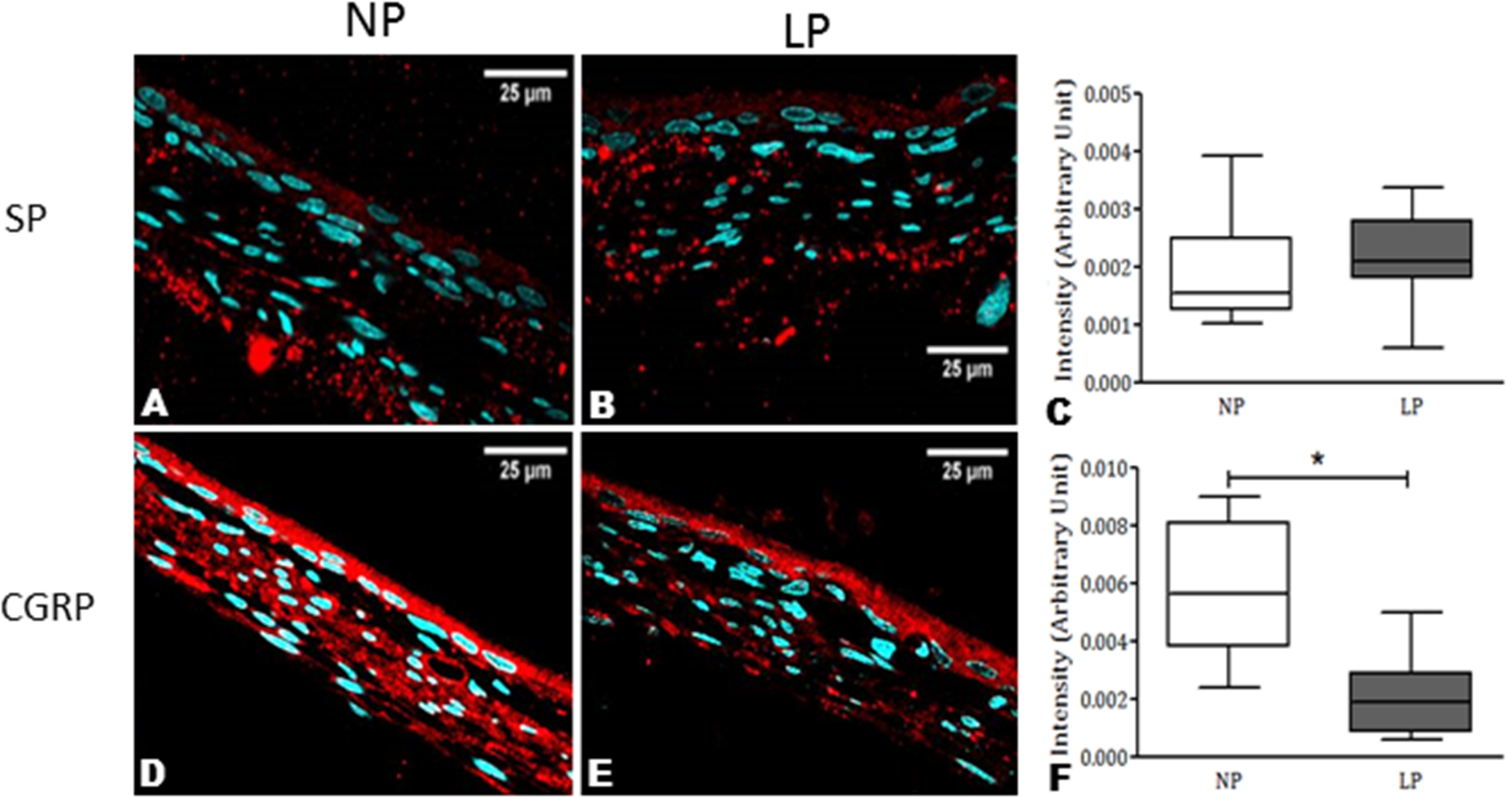
Comparative expression of SP and CGRP in the renal pelvis of 16-week-old rats. The pictures show a normal distribution of these neurokinins in NP (A and D). In LP offspring, no difference was observed in SP immunoreactivity (A, B, and C), but CGRP immunoreactivity was significantly more intense in NP (D, E, and F) compared with age-matched LP offspring. The data are reported as means ± SD. *P ≤ 0.05 vs. NP offspring (Student’s t-test).

NK_1_R staining was observed in all DRG neuronal subpopulations of 16-week-old NP (n = 15) and LP offspring (n = 15). NK_1_R immunostaining was significantly higher in LP rats than age-matched NP offspring (Fig 4A–4C). NK_1_R expression was significantly different in the nucleus, only in the intermediate (I) neurons of the LP offspring (p = 0.0002). On the other hand, NK_1_R expression was significantly higher in the cytosol of all LP subpopulation neurons (Fig 5; p < 0.0023). Conversely, SP and CGRP immunoreactivity in the DRG neurons of LP rats was significantly lower than NP offspring (Fig 4D–4I). SP was strongly and homogeneously expressed throughout the cell. In LP rats, SP immunoreactivity was reduced in the nucleus and cytosol of S and I neurons (p = 0.0001) when compared with age-matched NP offspring. CGRP neuronal immunoreactivity was reduced in the nucleus and cytosol of DRG cells in LP rats compared with NP rats (Fig 5; p = 0.0001). Quantification of cell size in five random sections of T13 DRGs revealed that the percentage of intermediate and large cells did not vary significantly between NP and LP offspring, but the number of small DRG cells was lower in LP offspring (LP: 17.1 ± 2.8% *vs*. age-matched NP: 27.7 ± 2.6%, n = 15 for each group, p = 0.001) (Fig 6). Additionally, SP immunoreactivity in the renal pelvis of LP offspring was similar to the NP group (A, B, and C), but CGRP immunoreactivity was significantly higher in NP (D, E, and F) than age-matched LP rats (Fig 7).

### Catecholamine quantification

As shown in Table 2, the plasma concentrations of NE (p = 0.0012), EPI (p = 0.0138) and DA (p = 0.0012) levels were higher in the LP than in the NP rats. In addition, the kidney NE levels was higher in LP than observed in NP rats (p = 0.0408). The effective renal denervation was confirmed by decreased renal NE concentration in both NP and LP denervated offspring when compared to sham-operated kidneys, (P < 0.0001).

**Table 2:** Plasma catecholamine quantification in controls (NP) and gestational low-protein (LP) offspring and, kidney tissue of bilateral renal denervated (DNx) groups compared to appropriate controls.

*Groups/Catecholamines*	*Norepinephrine*	*Epinephrine*	*Dopamine*
***NP (plasma*, *in pg/ml)***	128.0 ± 15.0	116.5 ± 32.8	132.0 ± 14.4
***LP (plasma*,* in pg/ml)***	275.0 ± 61.7^a^	169.4 ± 29.3^a^	177.3 ± 18.1^a^
***NP (kidney*, *in μg/g of tissue)***	223.1 ± 51.2	-	-
***NP***_***DNx***_*** (kidney*, *in μg/g of tissue)***	61.3 ± 41.2^b^	-	-
***LP (kidney*, *in μg/g of tissue)***	298.4 ± 67.3^b^	-	-
***LP***_***DNx***_*** (kidney*, *in μg/g of tissue)***	84.3 ± 28.7^c^	-	-

The data are reported as the means ± SD of *n* = 5 animals for each group. To plasma catecholamine levels analysis

^a^ *vs*. *NP* to *P* ≤ 0.001 (Student-t test); To kidney catecholamine concentrations analysis

^b^ *vs*. *NP* to *P* ≤ 0.05

^c^ *vs*. NP_DNx_ to P ≤ 0.05; (ANOVA with Bonferroni’s contrast test).

## Discussion

Environmental and genetic factors influence organ development, potentially leading to abnormal functional and structural effects in tissues and organs. Gestational protein restriction is associated with low birthweight and increased risk for the development of cardiovascular diseases, kidney dysfunction and metabolic syndrome in adult life [1–7]. The current study sustains a pathophysiological association between renal nerve activity, decreased renal sodium excretion, and enhanced blood pressure in maternal protein-deprived offspring model. The present data also confirmed that there was a significant reduction in the birthweight of LP male offspring compared with NP offspring [3–7]. This effect has been linked with a significant enhancement in arterial blood pressure beyond 7 weeks of age in LP rats relative to age-matched NP counterparts. Furthermore, the immunohistochemistry analyses demonstrated an increased expression of NK_1_ receptors and a reduced expression of the neurokinins SP and CGRP in the T13 DRGs of 16-week-old LP rats compared with NP rats. The renal pelvis of LP rats did not strongly express CGRP when compared with NP rats, whereas no change was observed in SP immunostaining.

Our results agree with previous studies, which demonstrated a marked and sustained rise in arterial blood pressure that was associated with a decreased urinary sodium excretion in LP offspring [3–7]. Thus, we hypothesized that offspring hypertension induced by maternal low-protein intake is associated, at least in part, with changes in renal neural activity and, consequently, in the renal sodium excretion. In addition, maternal low-protein intake reduced urinary sodium excretion by decreasing post-proximal tubule sodium rejection, although the creatinine clearance was not changed and sodium was normally filtered. The decreased renal potassium excretion observed in LP offspring when compared to age-matched NP group, suggest that the striking tubular sodium reabsorption in LP offspring occurs before distal nephron segment. However, the precise mechanism underlying the chronic arterial hypertension in offspring induced by maternal low-protein intake has not been clearly identified. Arterial pressure is thought to be controlled by the renal-mediated regulation of fluid and electrolytes. Experimental studies support the hypothesis that fetal programming is correlated with dysregulation of sodium transporters in different segments of nephron [37–39]. These alterations lead to a lower rate of urinary sodium excretion and sodium retention. However, which nephron segments and sodium transporters are affected by fetal programming remains unclear. To the best of our knowledge, the present findings demonstrate for the first time that bilateral renal denervation markedly attenuates the increase in arterial pressure and increased tubular sodium excretion in LP offspring. The enhanced urinary sodium excretion in LP_DNx_ offspring was associated to a reduction in proximal tubular sodium reabsorption that is incompletely, compensated by distal nephron segments. This increase urinary sodium excretion suggest an indirect but close relationship between enhanced renal nerve activity and attenuated sodium excretion in the development of hypertension in LP offspring. By the way, the progressive rises in arterial pressure in 16-wk old LP_DNx_ offspring may be related to kidney reinnervation [40,41].

The influence of the sympathetic nervous system (SNS) on renal function during development has not been well investigated in gestational protein-restricted model. Mizuno et al (2013) [42] proposed that increased blood pressure in protein-restricted offspring, in response to physical stress, would be associated with an increased renal sympathetic nerve activity (SNA) indicating a role for an increase in renal SNA in the developmental programming of increased blood pressure in this hypertensive model. Alexander and colleagues (2005), in placental insufficiency rat model, demonstrated that increased arterial pressure is abolished by bilateral renal denervation at 3 months of age in male offspring, and at 1-year old female offspring. These authors suggest that activation of the renal nerves is established in utero in male offspring, whereas an additional stimulus such as increased leptin plasma level in female intrauterine growth restriction offspring, may serve as a secondary stimulus demonstrating sex-specific programming of increased blood pressure [43–45]. Results of many studies have suggested that the SNS modulates renal function to influence the pathogenesis of hypertension. Electrical stimulation of the renal nerves in acute or chronic experiments enhances sodium reabsorption, particularly in the proximal convoluted tubule [46]. Additionally, electrical stimulation of the renal nerves at low frequency or intrarenal infusion of norepinephrine causes hypertension [47,48] by increasing sodium reabsorption in the proximal tubule and the loop of Henle. These effects are independent of changes in renal hemodynamics [46,49]. Taken together with previous findings [10,25,28,30,50,51], the current study demonstrated that bilateral renal denervation delays the development of arterial hypertension associated with reduced sodium reabsorption by the proximal and/or post-proximal tubule segments in this particular experimental model of developmental programming.

Although the rationale for renal denervation has generally been to interrupt sympathetic (efferent) nerve activity directed to the kidney, denervation of the renal plexus also deprives the kidney of its sensory innervation. Selective renal afferent nerves may have markedly widespread effects on the renorenal sympathetic reflexes and urinary sodium excretion [10,15,25,27]. Previous studies [10,25,27] have shown that increasing the renal pelvic pressure increased the release of SP and CGRP from the ipsilateral renal pelvis, contralateral urinary sodium excretion, and ipsilateral ARNA in rats. Additionally, studies [10,15,25,27] from normotensive rats demonstrated that SP and CGRP elicit a similar renorenal reflex response, including an increase in renal pelvic pressure. Moreover, treatment with SP and h-CGRP8–37 receptor antagonists or capsaicin, which depletes sensory neurons of SP, blocked the ARNA response, which increased renal pelvic pressure [10,15,25,27]. However, Kopp et al. [15] have demonstrated that increasing the renal pelvic pressure or pelvic administration of SP in spontaneously hypertensive rats failed to increase ARNA and did not elicit a contralateral renorenal reflex in these rats. The results of the current study show decreased SP and CGRP expression in T13 DRG cells in adult LP offspring compared with age-matched NP rats. In addition, NK_1_R immunoreactivity was significantly increased in the DRG cytosol and nucleus of 16-week-old LP offspring. NK_1_R is widely distributed in various tissues and organs and the high expression of NK_1_R in the sensory nervous system suggests that it plays an important role in regulating neuronal SP and CGRP synthesis. Although we have demonstrated that NK_1_R expression is increased in the DRG of LP offspring, little is known about the signaling pathways that regulate the NK_1_ receptor gene. Since the promoter region of the NK_1_ receptor gene contains a cAMP response element, we hypothesize that the decreased levels of SP and CGRP in DRG nuclei regulate the expression of NK_1_ receptors via a pathway involving activation of the transcription factor cAMP response element binding protein. Our findings that NK_1_R immunoreactivity is increased in DRG neurons may reflect a reduced synthesis of DRG neurokinins and explain a possible blunted renal sensory receptor activity in LP offspring. However, we cannot exclude the possibility that antinatriuresis observed in LP offspring could be associated with impaired neural responses to renal sensory receptor stimulation and defects in SP receptor-membrane (NK_1_) coupling mechanisms.

The present report showed higher renal and plasma catecholamine levels in LP offspring (Table 2) when compared to age-matched NP rats. In addition, as demonstrated in previous study [45], the bilateral renal denervation reduced kidney catecholamine concentrations in both NP and LP groups, though the decreased arterial blood pressure was observed only in growth-restricted offspring relative to renal denervated control rats. Previous studies have showed that arterial hypertension associated to renal nerve hyperactivity may involve alterations in tubular sodium reabsorption, arteriolar resistance and renin release [8,14,28,46,49]. Taking in account the current and previous studies, we may suggest a hyperactive state in the peripheral sympathetic nervous system, including to the kidneys, at least in part, caused by reduced afferent renal activity in LP offspring in adult life.

The current study has demonstrated a unimodal subpopulation distribution of SP and CGRP in NP and LP offspring, which skewed towards intermediate and large diameter cells. This skewed distribution and differences in subcellular staining showed that DRG neurons consist of various subpopulations. Significantly fewer small neurons were SP- and CGRP-positive in LP offspring compared with the NP group. On the other hand, more intermediate and large neurons were observed in NP and LP offspring. The percentage of SP- and CGRP-positive cells did not vary significantly between different DRG subpopulations in LP and NP rats. The precise relationship between primary afferent function and the neurochemical characterization of DRGs is still unclear. However, primary afferents of small and intermediate DRG neurons are important for the transmission of nociceptive, chemo, and mechanoreceptor information from the periphery to the CNS. They consist, respectively, of unmyelinated (*C*-conduction velocity) and thinly myelinated (*Aδ*-conduction velocity) fibers, which arise from a population of cells in the sensory ganglia [33,34]. Our results provide evidence that distinct sensory neuronal populations have different functions, which may involve a differential number (small number of unmyelinated and thinly myelinated neuron cells) and expression of neurotransmitters in response to afferent renal stimuli in LP offspring. Based on our findings, we assume the hypothesis that an impaired renorenal reflex activity in LP offspring may be associated with a decreased expression of SP and CGRP in DRG neurons, increasing the renal retention of sodium. Defects in the level of SP receptors have been reported in non-neural vascular tissue and axonal membrane of hypertensive subjects [52,53]. In addition, an increased pain threshold associated with a reduction of CNS SP levels has been reported in hypertensive men and rats [51–55]. Based on these observations, we suggest that the impaired response to natriuresis associated with hypertension in LP rats is partly related to a defect in SP and CGRP synthesis and release by renal sensory neurons.

Our findings raise the possibility of an impaired responsiveness of renal sensory receptors in maternal protein-restricted offspring is related to altered distribution of neurokinins and their receptors in DRG neurons and, consequently, decreased concentration of SP in the renal pelvis. In conclusion, although the precise mechanism for enhanced sodium retention in LP rats is still unclear, the current data suggested that changes in renal nerve activity and DRG neurokinin expression are conducive to excessive hydroelectrolytic tubule reabsorption, which might potentiate hypertension in LP offspring. These observations may provide a fresh framework for understanding the pathophysiology of impaired tubular sodium handling associated with the development of arterial hypertension in this programmed model.

## Supporting information

S1 File (DOCX)

S2 File (DOC)

S1 Thesis (PDF)

## References

[1] Ashton N. Perinatal development and adult blood pressure. Braz J Biol Res. 2000; 33: 731–740. 10.1590/s0100-879x2000000700002 10881047

[2] Barker DJP. In utero programming of chronic disease. Clin Sci. 1998; 95: 115–128. 9680492

[3] Mesquita FF, Gontijo JA, Boer PA. Expression of renin-angiotensin system signaling compounds in maternal protein-restricted rats: effect on renal sodium excretion and blood pressure. Nephrol Dial Transplant. 2010; 25: 380–388. doi: 10.1093/ndt/gfp505 19793932 10.1093/ndt/gfp505

[4] Mesquita FF, Gontijo JA, Boer PA. Maternal undernutrition and the offspring kidney: from fetal to adult life. Braz J Med Biol Res. 2010; 43: 1010–1018. 21049242 10.1590/s0100-879x2010007500113

[5] Dasinger JH, Davis GK, Newsome AD, Alexander BT. Developmental Programming of Hypertension: Physiological Mechanisms. Hypertension. 2016; 68(4):826–831. doi: 10.1161/HYPERTENSIONAHA.116.06603 27550912 10.1161/HYPERTENSIONAHA.116.06603 PMC5016247

[6] Vaccari B, Mesquita FF, Gontijo JA, Boer PA. Fetal kidney programming by severe food restriction: effects on structure, hormonal receptor expression and urinary sodium excretion in rats. J Renin Angiotensin Aldosterone Syst. 2015; 16(1):33–46. doi: 10.1177/1470320313481081 23482371 10.1177/1470320313481081

[7] Sene L de B, Mesquita FF, de Moraes LN, Santos DC, Carvalho R, Gontijo JA, Boer PA. Involvement of renal corpuscle microRNA expression on epithelial-to-mesenchymal transition in maternal low protein diet in adult programmed rats. PLoS One. 2013; 8(8): e71310 doi: 10.1371/journal.pone.0071310 23977013 10.1371/journal.pone.0071310 PMC3747155

[8] DiBona GF. Sympathetic neural control of the kidney in hypertension. Hypertension. 1992; 19: 28–35. 10.1161/01.hyp.19.1_suppl.i28 1730452

[9] Kopp UC, Smith LA. Inhibitory renorenal reflexes: a role for substance P or other capsaicin sensitive neurons. Am J Physiol. 1991; 260: R232–R239. 1704197 10.1152/ajpregu.1991.260.1.R232

[10] Oparil S. The renal afferent nerves in the pathogenesis of hypertension. Can J Physiol Pharmacol. 1987; 65: 1548–1558. 3319106 10.1139/y87-244

[11] Kopp UC, Smith LA. Inhibitory renorenal reflexes: a role for renal prostaglandins in activation of renal sensory receptors. Am J Physiol. 1991; 261: R1513–R1521. 1750575 10.1152/ajpregu.1991.261.6.R1513

[12] Gontijo JR, Smith LA, Kopp UC. CGRP activates renal pelvic substance P receptors by retarding substance P metabolism. Hypertension. 1999; 33: 493–498. 9931154 10.1161/01.hyp.33.1.493

[13] Gontijo JA, Kopp UC. Activation of renal pelvic chemoreceptors in rats: Role of calcitonin gene-related peptide receptors. Acta Physiol Scand. 1999; 166: 159–165. doi: 10.1046/j.1365-201x.1999.00540.x 10383496 10.1046/j.1365-201x.1999.00540.x

[14] Boer PA, Morelli JM, Figueiredo JF, Gontijo JA. Early altered renal sodium handling determined by lithium clearance in spontaneously hypertensive rats (SHR): role of renal nerves. Life Sci. 2005; 76: 1805–1815. doi: 10.1016/j.lfs.2004.09.029 15698858 10.1016/j.lfs.2004.09.029

[15] Kopp UC, Smith LA, DiBona GF. Impaired renorenal reflexes in spontaneously hypertensive rats. Hypertension. 1987; 9: 69–75. 3793202 10.1161/01.hyp.9.1.69

[16] Kopp UC, Olson LA, DiBona GF. Renorenal reflex responses to mechano- and chemoreceptor stimulation in the dog and rat. Am J Physiol. 1984; 246: F67–F77. 6696080 10.1152/ajprenal.1984.246.1.F67

[17] Moss NG. Electrophysiological characteristics of renal sensory receptors and afferent renal nerves. Miner Electrolyte Metab. 1989; 15: 59–65. 2644524

[18] Niijima A. Observation on the localization of mechanoreceptors in the kidney and afferent nerve fibers in the renal nerves in the rabbit. J Physiol. 1975; 245: 81–90. 1127615 10.1113/jphysiol.1975.sp010836 PMC1330846

[19] Ferguson M, Bell C. Ultrastructural localization and characterization of sensory nerves in the rat kidney. J Comp Neural. 1988; 247: 9–16. 10.1002/cne.902740103 2458398

[20] Liu L, Barajas L. The rat nerves during development. Anat Embriol. 1993; 188: 345–361. 10.1007/BF00185944 7506501

[21] Su HC, Wharton J, Polak JM, Mulderry PK, Ghate MA, Gibson SJ, Terenghi G, Morrison JF, Ballesta J, Bloom SR. Calcitonin gene-related peptide immunoreactivity in afferent neurons supplying the urinary tract: combined retrograde tracing and immunohistochemistry. Neuroscience. 1986; 18: 727–747. 2427972 10.1016/0306-4522(86)90066-7

[22] Zheng F, Lawson SN. Neurokinin A in rat renal afferent neurons and in nerve fibers within smooth muscle and epithelium of rat and guinea-pig renal pelvis. Neuroscience. 1997; 76: 1245–1255. 9027883 10.1016/s0306-4522(96)00441-1

[23] Seybold VS, McCarson KE, Mermelstein PG, Groth RD, Abrahams LG. Calcitonin gene-related peptide regulates expression of neurokinin1 receptors by rat spinal neurons. J Neurosci. 2003; 23: 1816–1824. 12629185 10.1523/JNEUROSCI.23-05-01816.2003 PMC6741973

[24] Hershey AD, Krause JE. Molecular characterization of a functional c DNA encoding the rat substance P receptor. Science. 1990; 247: 958–962. 2154852 10.1126/science.2154852

[25] Gerard NP, Garraway LA, Eddy RL, Shows TB, Iijima H, Paquet JL, et al Human substance P receptor (NK-1): Organization of the gene, chromosome localization, and functional expression of cDNA clones. Biochemistry. 1991; 30: 10640–10646. 1657150 10.1021/bi00108a006

[26] Pennefather JN, Lecci A, Candenas ML, Patak E, Pinto FM, Maggi CA. Tachykinins and tachykinin receptors: A growing family. Life Sci. 2004; 74: 1445–1463. 14729395 10.1016/j.lfs.2003.09.039

[27] De Koninck Y, Henry JL. Substance P-mediated slow excitatory postsynaptic potential elicited in dorsal horn neurons in vivo by noxious stimulation. Proc Natl Acad Sci USA. 1991; 88: 11344–11348. 1722327 10.1073/pnas.88.24.11344 PMC53131

[28] Xavier F, Magalhães AMF, Gontijo JAR. Effect of inhibition of nitric oxide synthase on blood pressure and renal sodium handling in renal denervated rats. Braz J Med Biol Res. 2000; 33: 347–354. 10719388 10.1590/s0100-879x2000000300014

[29] Ciampone S, Borges R, de Lima IP, Mesquita FF, Cambiucci EC, Gontijo JA. Long-term exercise attenuates blood pressure responsiveness and modulates kidney angiotensin II signalling and urinary sodium excretion in SHR. J Renin Angiotensin Aldosterone Syst. 2011; 12(4): 394–403. doi: 10.1177/1470320311408750 21628355 10.1177/1470320311408750

[30] Lutaif NA, Gontijo LM, Figueiredo JF, Gontijo JA. Altered urinary sodium excretion response after central cholinergic and adrenergic stimulation of adult spontaneously hypertensive rats. J Physiol Sci. 2015; 65(3): 265–275. doi: 10.1007/s12576-015-0364-9 25690463 10.1007/s12576-015-0364-9 PMC10717338

[31] Waters JC. Live-cell fluorescence imaging. Methods Cell Biol. 2007; 81:115–140. doi: 10.1016/S0091-679X(06)81007-1 17519165 10.1016/S0091-679X(06)81007-1

[32] Murray JM, Appleton PL, Swedlow JR, Waters JC. Evaluating performance in three-dimensional fluorescence microscopy. J. Microsc. 2007; 228:390–405. doi: 10.1111/j.1365-2818.2007.01861.x 18045334 10.1111/j.1365-2818.2007.01861.x PMC2438600

[33] Alvarez F. J., Morris H. R., and Priestley J. V. Sub-populations of smaller diameter trigeminal primary afferent neurons defined by expression of calcitonin gene-related peptide and the cell surface oligosaccharide recognized by monoclonal antibody LA4. J. Neurocytol. 1991; 20:716–731. 1960536 10.1007/BF01187846

[34] Aline Boer P, Ueno M, Sant’ana JS, Saad MJ, Gontijo JAR. Expression and localization of NK1R, substance P and CGRP are altered in dorsal root ganglia neurons of spontaneously hypertensive rats (SHR). Mol. Brain Res. 2005; 138:35–44. doi: 10.1016/j.molbrainres.2005.03.015 15869822 10.1016/j.molbrainres.2005.03.015

[35] Di Marco GS, Naffah-Mazzacoratti Md Mda G, Vio CP. Mesangial cells are able to produce catecholamines in vitro, J Cell Biochem. 2003; 89: 144–151. doi: 10.1002/jcb.10485 12682915 10.1002/jcb.10485

[36] Anton AH, Sayre DF. A study of the factors affecting the aluminum oxide-trihydroxyindole procedure for the analysis of catecholamines, J Pharmacol Exp Ther. 1962; 138: 360–375. 14013351

[37] Manning J, Beutler K, Knepper MA, Matti Vehaskari V. Upregulation of renal BSC1 and TSC in prenatally programmed hypertension. Am J Physiol. 2002; 283: F202–F206. 10.1152/ajprenal.00358.2001 12060603

[38] Alwasel SH, Ashton N. Prenatal programming of renal sodium handling in the rat. Clin Sci. 2009; 117: 75–84. doi: 10.1042/CS20080294 19128240 10.1042/CS20080294

[39] Baum M. Role of the kidney in the prenatal and early postnatal programming of hypertension. Am J Physiol. 2010; 298: F235–F247. 10.1152/ajprenal.00288.2009 PMC2822514 19794108

[40] Kline RL. Renal nerves and experimental hypertension: evidence and controversy. Can J Physiol Pharmacol 1987; 65: 1540–1547. 3319105 10.1139/y87-243

[41] Oparil S. The renal afferent nerves in the pathogenesis of hypertension. Can J Physiol Pharmacol. 1987; 65: 1548–1558. 3319106 10.1139/y87-244

[42] Mizuno M, Siddique K, Baum M, Smith SA. Prenatal programming of hypertension induces sympathetic overactivity in response to physical stress. Hypertension. 2013; 61(1):180–186. doi: 10.1161/HYPERTENSIONAHA.112.199356 23150514 10.1161/HYPERTENSIONAHA.112.199356 PMC3525329

[43] Alexander BT, Hendon AE, Ferril G, Dwyer TM. Renal denervation abolishes hypertension in low-birth-weight offspring from pregnant rats with reduced uterine perfusion. Hypertension. 2005; 45(4):754–8. doi: 10.1161/01.HYP.0000153319.20340.2a 15699462 10.1161/01.HYP.0000153319.20340.2a

[44] Ojeda NB, Grigore D, Yanes LL, Iliescu R, Robertson EB, Zhang H, Alexander BT. Testosterone contributes to marked elevations in mean arterial pressure in adult male intrauterine growth restricted offspring. Am J Physiol Regul Integr Comp Physiol. 2007; 292: R758–R763. doi: 10.1152/ajpregu.00311.2006 16917022 10.1152/ajpregu.00311.2006

[45] Intapad S, Tull FL, Brown AD, Dasinger JH, Ojeda NB, Fahling JM, Alexander BT. Renal denervation abolishes the age-dependent increase in blood pressure in female intrauterine growth-restricted rats at 12 months of age. Hypertension. 2013; 61(4):828–834. doi: 10.1161/HYPERTENSIONAHA.111.00645 23424240 10.1161/HYPERTENSIONAHA.111.00645 PMC3626267

[46] DiBona GF. Neural control of the kidney: functionally specific renal sympathetic nerve fibers. Am J Physiol. 2000; 279: R1517–R1524. 10.1152/ajpregu.2000.279.5.R1517 11049831

[47] Collis MG, DeMey C, Vanhoutte PM. Renal vascular reactivity in young hypertensive rats. Hypertension. 1980; 2: 45–52. 6966255 10.1161/01.hyp.2.1.45

[48] Patel KP, Kline RL, Mercer PF. Noradrenergic mechanism in the brain and peripheral organs of normotensive and spontaneously hypertensive rats at various ages. Hypertension. 1981; 3: 682–690. 6117514 10.1161/01.hyp.3.6.682

[49] DiBona GF. Nervous Kidney. Interaction between renal sympathetic nerves and rennin-angiotensin system in the control of renal function. Hypertension. 2000; 36: 1083–1088. 11116129 10.1161/01.hyp.36.6.1083

[50] Rudd MA, Grippo RS, Arendshorst WJ. Acute renal denervation produces diuresis and natriuresis in young SHR but not WKy rats. Am J Physiol. 1986; 251: F655–F661. 3766742 10.1152/ajprenal.1986.251.4.F655

[51] Schobel HP, Ringkamp M, Behrmann A, Forster C, Schmieder RE, Handwerker HO. Hemodynamic and sympathetic nerve responses to painful stimuli in normotensive and borderline hypertensive subjects. Pain. 1996; 66: 117–124. 8880832 10.1016/0304-3959(96)03079-5

[52] Huang A, Koller A. Both nitric oxide and prostaglandin-mediated responses are impaired in skeletal arterioles of hypertensive rats. J Hypertens. 1996; 14: 887–895. 8818928 10.1097/00004872-199607000-00012

[53] Quyyumi AA, Mulcahy D, Andrews NP, Husain S, Panza JA, Cannon RO. Coronary vascular nitric oxide activity in hypertension and hypercholesterolemia. Circulation. 1997; 95: 104–110. 8994424 10.1161/01.cir.95.1.104

[54] Sitsen JMA, De Jong W. Hypoalgesia in genetically hypertensive rats is absent in rats with experimental hypertension. Hypertension. 1983; 5: 185–190. 6826214 10.1161/01.hyp.5.2.185

[55] Virus RM, Knuepfer MM, Mcmanus DQ, Brody MJ, Gebhart GF. Capsaicin treatment in adult Wistar–Kyoto and spontaneously hypertensive rats: effects on nociceptive behavior and cardiovascular regulation. Eur J Pharmacol. 1981; 72: 209–217. 6166489 10.1016/0014-2999(81)90275-2

