# Supplementary material for: Renal sodium handling and blood pressure changes in gestational protein-restricted offspring: Role of renal nerves and ganglia neurokinin expression
Source: PLoS One. 2017 Jun 20;12(6):e0179499. doi: 10.1371/journal.pone.0179499 (PMC5478103; doi:10.1371/journal.pone.0179499)
Supplement: S2 File — (DOC) [file pone.0179499.s002.doc]

RENAL SODIUM LEVELS AND BLOOD PRESSURE CHANGES IN GESTATIONAL PROTEIN-RESTRICTED OFFSPRING: ROLE OF RENAL NERVES AND GANGLIA NEUROKININ EXPRESSION

Running Title: Gestational low-protein intake and renal nerve activity

Augusto H. Custódio, Marcelo C. de Lima, Bárbara Vaccari, Patrícia A. Boer, José A. R. Gontijo

Department of Internal Medicine School of Medicine, State University of Campinas, 13083-887 Campinas, SP, Brazil

Competing interest: We affirm that there are no conflicts of interest

Acknowledgments: Grants from CNPq (No. 500868/91-3), CAPES and FAPESP (2013/12486-5) supported this work.

Correspondence:

J.A.R. Gontijo, Departamento de Clínica Médica, Faculdade de Ciências Médicas, Universidade Estadual de Campinas, 13083-592 Campinas, SP, Brazil.

Phone: 55-19-3521 8019; FAX: 55-19-3521 8925

E-mail: [gontijo@fcm.unicamp.br](mailto:gontijo@fcm.unicamp.br)

ABSTRACT

**Background.** Long-term changes in renal sodium handling have been demonstrated in maternal protein-restricted (LP) offspring; therefore, we hypothesized that hypertension may occur in LP offspring, at least in part, as a result of sustained renal sympathetic nerve overactivity and abnormal neuropeptide expression in renal sensory nerves. To investigate this, we examined whether low protein intake during pregnancy altered the expression of SP, CGRP, and NK1 receptors in the kidney and dorsal root ganglia (DRG). In addition, we investigated whether modified neurokinin expression was associated with changes in urinary sodium excretion and blood pressure in 16-week-old male LP offspring.

**Methods.** Pregnant dams received a normal (NP) or low-protein diet (LP) for the duration of their pregnancy. In renal bilaterally denervated male NP or LP offspring, surgery was performed prior to the start of the 8-week renal function test and blood pressure measurement. DRG cells were studied by confocal laser scanning microscopy.

**Results.** We confirmed a reduced birthweight in male LP offspring compared with age-matched NP offspring after 7 weeks of age, which was associated with enhanced blood pressure. Immunohistochemical analyses demonstrated an increased expression of NK1 receptors and a reduced expression of SP and CGRP in the DRGs of 16-week-old LP offspring. These findings show that kidney denervation attenuates increased arterial pressure and enhances urinary sodium excretion in LP offspring.

**Conclusions.** To the best of our knowledge, this study is the first to show increased natriuresis associated with reduced blood pressure after bilateral renal denervation in LP compared with NP offspring. Our findings indicate a functional relationship between renal nerve activity, attenuated sodium excretion, and the development of hypertension in adult LP offspring.

***Keywords***: Low-protein diet, fetal programming, arterial hypertension, renal nerve, SP, CGRP, NK1 receptors, renal function

INTRODUCTION

Disruptions in fetal programming result in low birthweight, fewer nephrons, and increase the risk of cardiovascular and renal disorders in adults [1–4]. We recently demonstrated that gestational low-protein (LP) intake offspring have a lower birthweight, arterial hypertension, and 30% fewer nephrons [3,4]. In addition, hydroelectrolytic balance studies have shown that arterial hypertension in gestational protein-restricted offspring is associated with decreased renal salt and water excretion when compared with pair-fed, age-matched normal protein intake (NP) rats [3–6].

Increased sympathetic nerve traffic causes detrimental hypertension leading to end organ damage [7–10]. Efferent renal nerve activity (ERNA) is enhanced in many models of hypertension and may be related to water and salt retention, both of which contribute to hypertension [11–13]. Renal sensorial afferent neurons are predominantly unmyelinated and project to the T10–L3 ipsilateral dorsal root ganglia (DRG) from sensory receptors located in the renal veins, arteries, and renal pelvic wall [14–19]. Previous studies in rats have shown that the activation of renal mechanoreceptors (MR) or renal chemoreceptors (CR) increases ipsilateral afferent renal nerve activity (ARNA). This is associated with a decrease in contralateral ERNA and an increased urinary flow rate and natriuresis. These findings indicate a contralateral inhibitory renorenal reflex response [14,20,21]. Calcitonin gene-related peptide (CGRP) and substance P (SP) colocalize in many neurons and CGRP-expressing neurons are more abundant than SP neurons in the renal pelvic wall [22,23]. Close to 90% of neurons in T10–L3 DRGs express CGRP [24,25] and 24% of renal afferent neurons express SP [24].

SP is a tachykinin with nanomolar affinity and it interacts with specific G-protein-coupled membrane receptors (GPCRs) [25]. Three distinct tachykinin receptors, NK_1_, NK_2_, and NK_3_, have now been cloned in different species [26,27]. NK_1_ receptors (NK_1_R) are widely distributed in the renal pelvis and brain, and have been implicated in nociceptive signaling to the spinal cord [28]. Furthermore, CGRP regulates the expression of NK_1_R in rat spinal neurons [29] and retards the metabolism of SP [20], thereby increasing the amount of SP available for SP receptor stimulation.

Long-term changes in renal sodium handling have been demonstrated in LP offspring [5–8]; therefore, we hypothesized that hypertension in LP offspring may be partly caused by sustained renal sympathetic nerve overactivity accompanied by abnormal expression of renal sensory nerve neuropeptides. This could promote a reduction of urinary sodium excretion and increased arterial hypertension. To investigate this, we measured the glomerular filtration rate and tubular sodium levels by creatinine and lithium clearance in conscious, gestational protein-restricted renal-denervated offspring and compared these measurements with low-protein and normal maternal food intake sham-operated rats. In addition, we examined the expression and localization of NK_1_R, SP, CGRP, SP receptors, and CGRP receptors in DRG (T13) cells in LP offspring and NP controls.

MATERIALS AND METHODS

***Animals—***The experiments were conducted on age-matched offspring of sibling-mated Wistar HanUnib rats (250–300 g). The experimental protocol and study design were approved by the Institutional Animal Ethics Committee *(protocol #2575-1)* and followed the general guidelines established by the Brazilian College of Animal Experimentation (COBEA). Our local colonies originated from a breeding stock supplied by CEMIB/Unicamp, Campinas, SP, Brazil. Immediately after weaning at 3 weeks of age, offspring were maintained under controlled conditions of 25°C and a 12-h light–dark cycle, with free access to tap water and standard rodent laboratory chow (Nuvital, Curitiba, PR, Brazil). Detection of sperm in the vaginal smear was designated as day 1 of pregnancy. Pregnant dams were fed isocaloric standard rodent laboratory chow with normal 17% protein content (NP) or low 6% protein (LP) content *ad libitum* throughout the entire pregnancy. All dams received NP chow after delivery. Food consumption was monitored daily and normalized to the body weight. Body weight was recorded weekly. Male pups were weighed, and fed NP chow until 16 weeks of age.

***Surgical procedures—***For renal bilaterally denervated offspring [NP_DNx_ (n = 9) and LP_DNx_ (n = 8)], surgery was performed before the 8-week renal functional test and blood pressure measurements. Briefly, the animals were anesthetized with a mixture of ketamine [75 mg/kg^−1^ body weight, injected intraperitoneally (i.p.)] and xyla**z**ine (10 mg/kg^−1^ body weight, i.p.). Once the corneal and pedal reflexes were absent, both kidneys were exposed by dorsal abdominal incisions and surgically denervated with the aid of a stereomicroscope. Denervation was performed by cutting all visible nerves along the renal artery and by stripping the connective tissue passing by and along the course of the renal artery and vein. Immediately after, the renal vessels were wrapped and surrounded with cotton swabs soaked in 10% (v/v) phenol diluted in absolute ethanol [8,30]. Sham-operated rats underwent all surgical procedures but the renal artery was left intact. Rats were used for experiments 1 week after renal denervation. Rats were observed individually in metabolic cages under controlled temperature, light (12-h light–dark cycle), and humidity.

***Blood pressure measurement—***The systolic arterial pressure was measured in conscious offspring at 6, 8, 10, 12, 14, and 16 weeks of age (NP n = 9, NP_DNx_ n = 9, LP and LP_DNx_ n = 8, and LP n = 8). Blood pressure was measured using an indirect tail-cuff method with an electrosphygmomanometer (IITC Life Science***—***BpMonWin Monitor Version 1.33) combined with a pneumatic pulse transducer/amplifier. This indirect approach allowed repeated measurements with close correlation (correlation coefficient = 0.975) compared with direct intra-arterial recording. The mean of three consecutive readings was taken as the blood pressure.

***Renal function evaluation—***Renal function was measured by creatinine and lithium clearance at 10 (NP n = 9, NP_DNx_ n = 9, LP and LP_DNx_ n = 8), and 16 (LP n = 8, NP n = 8, NP_DNx_ n = 8, LP and LP_DNx_ n = 8) weeks of age in unanaesthetized, unrestrained male offspring. Briefly, 14 h before renal function was tested, 60 µmol/100g^−1^ body weight LiCl was administered by gavage. After an overnight fast, each animal received tap water by gavage (5% of body weight), followed by a second load of the same volume 1 h later. Spontaneously voided urine was collected over a 120-min period into a graduated centrifuge tube. At the end of the experiment, blood samples were drawn through a cardiac puncture in anesthetized rats, and urine and plasma samples were collected for analysis [3–6,31,32].

***Immunofluorescence detection of NK1, SP, and CGRP—***Sixteen-week-old male NP (n = 3), and LP (n = 3) rats were used for immunofluorescence experiments. Rats were anesthetized and perfused with saline containing 2% heparin via the left carotid artery for 5 min under constant pressure. This was followed by perfusion with 0.1 M phosphate buffer (pH 7.4) containing 4% (w/v) paraformaldehyde and 0.1 M sucrose. After perfusion, the left T13 DRG and kidneys were immediately removed and placed in the same fixative for 1 h, followed by phosphate-buffered saline (PBS) containing 0.1% glycine for 1 h and PBS containing 15% (w/v) sucrose overnight. Then, tissues were immersed in OCT cryoprotector (Tissue-tech) and frozen in liquid nitrogen (−79°C). Sections (7 µm thick) were cut at −22°C using a Leica cryostat and collected on saline-coated slides. For immunohistochemistry, sections were blocked in PBS containing 3% normal donkey serum and 3% bovine serum albumin for 45 min to minimize nonspecific reactions. After blocking, sections were labeled with rabbit anti-NK1R antiserum (1:100 dilution; Novus Biologicals®), rabbit anti-CGRP antiserum (1:100 dilution; Neuromics®), or goat anti-SP antiserum (1:600; Santa Cruz), at 4°C overnight. Then, sections were incubated in anti-rabbit DyLight® 594-labeled secondary antibody (1:200 dilution) or rabbit anti-goat Cy™ 3-labeled secondary antibody (1:200 dilution) for 2 hours at room temperature. Then, sections were rinsed in 0.1 M PBS and cover-slipped with Vectashield anti-fading medium containing DAPI (Vector). The sections were examined with a confocal laser scanning microscope (LSM 780***—***ZEISS) and digital images were captured using specific software (LSM; Zeiss). No immunoreactivity was observed when the primary antibodies were omitted.

***Cellular population analysis—***Immunofluorescence staining in DRG cells was assessed in optical sections by confocal laser scanning microscope (CLSM). Using this approach, immunostaining was easily recognized. To determine the cell size, five random DRG sections immunostained with NK_1_R, SP, and CGRP antisera were selected from three different T13 DRG groups. Images were analyzed using Image J software. Briefly, the boundaries of immunostained cells and nuclei profiles were traced manually using a computer mouse and the intensity of fluorescence and cell areas were automatically calculated. To compare our results with previous quantitative studies on DRGs, the cell areas were transformed into cell diameters by assuming that ganglion cells are circular and that the ranges of diameters in the different subpopulations were 10–25 µm for small cells, 25–37.5 µm for intermediate cells, and 37.5–60 µm for large cells. All quantified sections were optimally stained that same day.

***Data presentation and statistical analysis—***Results were expressed as mean ± standard deviation (SD), scatter dot plots, or median and quartile deviation as appropriate. Creatinine clearance (C_Cr_) was used to estimate glomerular filtration rate (GFR) and lithium clearance (C_Li_) was used to assess proximal tubule output. Fractional sodium excretion (FE_Na_) was calculated as C_Na_/C_Cr_×100, where C_Na_ is sodium clearance. Fractional proximal (FEP_Na_) and post-proximal (FEPP_Na_) sodium excretion were calculated as C_Li_/C_Cr_ × 100 and C_Na_/C_Li_ × 100, respectively [3–6,31,32]. Data obtained over time were analyzed using a repeated measures one-way ANOVA or nonparametric analysis using the Kruskal–Wallis test. When statistically significant differences were indicated between selected means by ANOVA, *post hoc* comparisons were performed with Bonferroni’s contrast test. Comparisons involving only two means within or between groups were carried out using a Student’s t*-*test. The level of significance was set at P ≤ 0.05.

RESULTS

The serum sodium, lithium, and potassium levels from NP and LP sham and denervated offspring are presented in Table 1 and show no significant differences in NP rats compared with the LP group. In general, food and sodium intake were similar in male offspring of NP and LP groups when normalized by body weight (Table 1). Maternal protein restriction during pregnancy did not significantly change the body mass of pregnant dams during gestation (Figure 1A). In addition, it did not affect the number of offspring per litter and the proportion of male and female offspring (P = 0.3245). The birthweight of LP male pups (n = 26) was significantly reduced compared with NP pups (n = 21) (6.06 ± 0.075 g *vs.* 7.44 ± 0.10 g; P = 0.001) (Table 1 and Figure 1B). The body mass of LP pups (67.9 ± 0.99 g, n = 17) remained lower than age-matched NP pups (72.10 ± 1.42 g, n = 20) until weaning at 21 days after birth (P = 0.05) (Figure 1C). However, after 10 weeks of age, the body weight of LP and NP rats was not significantly different. Bilateral renal denervation did not affect body mass in any experimental group (Figure 1C).

***Blood pressure measurement—***As shown in Figure 2A, tail systolic arterial pressure (mmHg) was significantly higher in LP offspring compared with NP offspring between 7 weeksand 16 weeks of age. The changes in systolic blood pressure from 7 weeks to 16 weeks of age were as follows: 7 weeks: LP, 147 ± 8.9 mmHg *vs*. NP, 130.9 ± 8.1 mmHg, P = 0.001; 16 weeks: LP, 148.8 ± 11.7 mmHg *vs*. NP, 131 ± 1.9 mmHg, P = 0.001 (Figure 3A). Figure 2 also shows the effect of bilateral renal denervation on blood pressure at 8 weeks of age. The continuous increase in blood pressure in LP offspring was significantly reduced by bilateral renal denervation (Figure 2B) over an 8-week period (between 10 weeks and 16 weeks of age). Renal bilateral phenol denervation significantly prevented arterial pressure increase for up to 8 weeks compared with the non-denervated LP group (P = 0.001). In addition, there was no significant difference in blood pressure between the LP renal bilaterally denervated rats and the NP group (Figure 2C). This attenuation in blood pressure was associated with a significant increase in urinary sodium excretion and a decrease in proximal sodium reabsorption, as described below.

***Renal function evaluation—***Renal function in 10- and 16-week-old NP and LP offspring is summarized in Figure 3. The urinary flow rates (data not included) and the GFR, estimated by C_Cr_, did not significantly differ between the groups even after bilateral renal denervation. Fractional urinary sodium excretion (FE_Na_, Figure 3B) in 10-week old LP rats was unchanged compared with age-matched NP rats (LP: 0.1562 ± 0.041% *vs.* NP: 0.1935 ± 0.051%; P = 0.1347). However, renal denervation in 10-week old LP_DNx_ rats transiently enhanced fractional urinary sodium excretion compared with LP non-denervated and NP_DNx_ rats. The enhanced FE_Na_ in renal-denervated LP rats (0.2894 ± 0,047%, P = 0.0001) was accompanied by a significant increase in proximal sodium excretion (LP_DNx_: 28.15 ± 3.38% *vs.* LP: 19.11 ± 1.27%; P = 0.0127), while FEPP_Na_ and FE_K_ were unchanged compared with age-matched sham-operated offspring. Similar to 10-week-old rats, 16-week-old NP and LP urinary flow rates (data not included) and GFRs estimated by CCr did not significantly differ among the all studied groups. At this age, fractional urinary sodium excretion was significantly lower in 16-week-old LP rats compared with age-matched NP offspring (16-week-old LP: 0.089 ± 0.006% *vs.* NP: 0.199 ± 0.028%; P = 0.006). The decreased FE_Na_ in LP rats was accompanied by a significant reduction in FEPP_Na_ (16-week-old LP: 0.324 ± 0.031% *vs.* NP: 0.589 ± 0.07%; P = 0.0013) and FE_K_ (16-week-old LP: 0.046 ± 0.006% *vs.* NP: 0.0973 ± 0.008%; P = 0.0002), compared with age-matched NP control rats. At 16 weeks of age, the FE_Na_ increase in LP offspring caused by renal denervation was significantly attenuated and sodium excretion levels were similar to non-denervated NP and LP rats (Figure 3B). Likewise, increased proximal sodium excretion in renal denervated LP rats at 10 weeks of age was not detected at 16 weeks of age.

***NK_1_R, SP, and CGRP immunostaining in DRG cells—***NK_1_R staining was observed in all DRG neuronal subpopulations of 16-week-old NP and LP offspring. NK_1_R immunostaining was significantly higher in LP rats than age-matched NP offspring (Figure 4A–C). NK_1_R expression was detected in the nucleus and cytosol and only in the intermediate (I) neurons of LP offspring. NK_1_R expression was significantly higher in the cytosol of all LP neurons (Figure 5). Conversely, SP and CGRP immunoreactivity in the DRG neurons of LP rats was significantly lower than NP offspring (Figures 4D–I). SP was strongly and homogeneously expressed throughout the cell. In LP rats, SP immunoreactivity was reduced in the nucleus and cytosol of S and I neurons compared with age-matched NP offspring.

CGRP neuronal immunoreactivity was reduced in the nucleus and cytosol of DRG cells in LP rats compared with NP rats (Figure 5). Quantification of cell size in five random sections of T13 DRGs revealed that the percentage of intermediate and large cells did not vary significantly between NP and LP offspring, but the number of small DRG cells was lower in LP offspring (LP: 17.1 ± 2.8% *vs.* age-matched NP: 27.7 ± 2.6%, n = 15 for each group, P = 0.001) (Figure 6). Additionally, SP immunoreactivity in the renal pelvis of LP offspring was similar to the NP group (A, B, and C), but CGRP immunoreactivity was significantly higher in NP (D, E, and F) than age-matched LP rats (Figure 7).

Discussion

Previous studies have shown that kidney development is influenced by modifications in the intrauterine environment [1–6]. This organizational phenomenon is termed “early-life programming.” Here, in a maternal protein-deprived offspring model, we hypothesized an association between neural kidney activity and abnormal renal sodium levels. We suggested that permanent changes in the kidney underlie, at least in part, the development of arterial hypertension. We confirmed that the birthweight of LP male offspring was significantly reduced compared with NP offspring [3–6]. This effect was associated with a significant enhancement in arterial blood pressure after 7 weeks of age in LP rats compared with NP rats. Furthermore, our immunohistochemistry analyses demonstrated an increased expression of NK_1_ receptors and a reduced expression of the neurokinins SP and CGRP in the T13 DRGs of 16-week-old LP rats compared with NP rats. CGRP was not strongly expressed in the renal pelvis of LP rats compared with NP rats, whereas no change was observed in SP immunostaining. We also observed a reduced FE_Na_ after 10 weeks of age, which was significantly more pronounced in 16-week-old LP offspring. This may have been caused by a variety of mechanisms, such as post-glomerular arteriolar resistance, decreased renal blood flow, overexcitation of renal sympathetic nerves, and/or direct tubule effects in LP offspring, which may have elevated the arterial blood pressure. Moreover, the higher renal sodium levels in 16-week-old LP rats may be attributed to abnormalities in intrinsic renal mechanisms. We previously demonstrated a normal whole-kidney GFR in LP offspring, despite a lower number of glomeruli using different models of fetal programming, which is indicative of a compensatory glomerular hyperfiltration mechanism [3–6]. Thus, a reduction in nephron number [3–6] and whole-kidney glomerular filtration area would reduce urinary sodium excretion, enhancing susceptibility to hypertension and reducing renal reserve. Therefore, limited compensation for renal injury may, at least in part, explain the higher prevalence of hypertension and renal disease observed in populations with lower birthweights [2–4].

We have previously demonstrated that the expression of AngII receptors and signaling compounds is dramatically downregulated in LP rats, suggesting that mechanisms other than antinatriuresis are present in these animals [5–8]. We did not rule out an indirect mechanism for the decreased renal sodium excretion. Selkurt et al. (1965) and others [33] have proposed that decreased peritubular pressure might stimulate sodium reabsorption by decreasing modular blood flow. Additionally, Cowley (1997) confirmed that a reduced interstitial volume and/or pressure across the peritubular capillaries could enhance tubular sodium reabsorption [34]. In the present study, hemodynamic glomerular changes increased the fractional filtration rate and/or decreased the renal blood flow in LP rats, which may have enhanced sodium reabsorption in proximal segments of the nephron.

The present study tested the hypothesis that offspring hypertension induced by maternal low-protein intake is related, at least in part, to changes in renal neural activity and renal sodium excretion. Our results agree with previous studies, which demonstrated a marked and sustained rise in arterial blood pressure that was associated with a decrease in urinary sodium excretion [3–6]. In addition, maternal low-protein intake reduced urinary sodium excretion by decreasing proximal tubule sodium rejection, although the GFR was not changed and sodium was normally filtered. The precise mechanism underlying the chronic arterial hypertension in offspring induced by maternal low-protein intake has not been identified. Arterial pressure is thought to be controlled by the renal-mediated regulation of fluid and electrolytes. To the best of our knowledge, the present findings demonstrate for the first time that bilateral renal denervation markedly attenuates the increase in arterial pressure and increased tubular sodium excretion in LP rats. Enhanced urinary sodium excretion is related to a reduction in proximal tubular sodium reabsorption that is incompletely compensated by distal nephron segments. This increase in proximal sodium excretion provides evidence of a functional relationship between renal nerve activity and attenuated sodium excretion in the development of hypertension in LP rats. Furthermore, the rise in arterial pressure until 16 weeks of age may be related to renal reinnervation and increased norepinephrine content [35,36]. Complete renal denervation delayed the rise in blood pressure in other rat models of hypertension, including spontaneous hypertension and angiotensin-II-induced hypertension [37,38]. However, no effect was reported in salt-sensitive Dahl rats and the prevention of hypertension by renal denervation remains controversial in DOCA-salt and one-kidney, one clip models [37,38].

The influence of the sympathetic nervous system (SNS) on renal function during development has not been well investigated. Results of several studies have suggested that the SNS modulates renal function to influence the pathogenesis of hypertension. Electrical stimulation of the renal nerves in acute or chronic experiments enhances sodium reabsorption, particularly in the proximal convoluted tubule [39]. Additionally, electrical stimulation of the renal nerves at low frequency or intrarenal infusion of norepinephrine causes hypertension [40,41] by increasing sodium reabsorption in the proximal tubule and the loop of Henle. These effects are independent of changes in renal hemodynamics [39,42]. Taken together with previous findings [10,13,27,30,32,43,48], we have shown that bilateral renal denervation delays the development of arterial hypertension associated with reduced sodium reabsorption by the proximal and/or post-proximal tubule segments in different hypertensive experimental models.

Although the rationale for renal denervation has generally been to interrupt sympathetic (efferent) nerve activity directed to the kidney, denervation of the renal plexus also deprives the kidney of its sensory enervation. Selective renal afferent nerves may have markedly widespread effects on the renorenal sympathetic reflexes and urinary sodium excretion [13,27,28,44]. Previous studies [13,27,28] have shown that increasing the renal pelvic pressure increased the release of SP and CGRP from the ipsilateral renal pelvis, contralateral urinary sodium excretion, and ipsilateral ARNA in rats. Additionally, studies [13,27,28,44] in normotensive rats demonstrated that SP and CGRP elicit a similar renorenal reflex response, including an increase in renal pelvic pressure. Moreover, treatment with SP and h-CGRP8–37 receptor antagonists or capsaicin, which depletes sensory neurons of SP, blocked the ARNA response, which increased renal pelvic pressure [13,27,28,44]. However, Kopp et al. [44] have demonstrated that increasing the renal pelvic pressure or pelvic administration of SP in spontaneously hypertensive rats failed to increase ARNA and did not elicit a contralateral renorenal reflex in these rats. The results of the current study show decreased SP and CGRP expression in T13 DRG cells in adult LP offspring compared with age-matched NP rats. In addition, NK_1_R immunoreactivity was significantly increased in the DRG cytosol and nucleus of 16-week-old LP offspring. NK_1_R is widely distributed in various tissues and organs and the high expression of NK_1_R in the sensory nervous system suggests that it plays an important role in regulating neuronal SP and CGRP synthesis. Although we have demonstrated that NK_1_R expression is increased in the DRG of LP offspring, little is known about the signaling pathways that regulate the NK_1_ receptor gene. Since the promoter region of the NK_1_ receptor gene contains a cAMP response element, we hypothesize that the decreased levels of SP and CGRP in DRG nuclei regulate the expression of NK_1_ receptors via a pathway involving activation of the transcription factor cAMP response element binding protein. Our findings that NK_1_R immunoreactivity is increased in DRG neurons may reflect a reduced synthesis of DRG neurokinins and explain a possible blunted renal sensory receptor activity in LP offspring. However, we cannot exclude the possibility that antinatriuresis observed in LP offspring could be associated with impaired neural responses to renal sensory receptor stimulation and defects in SP receptor-membrane (NK_1_) coupling mechanisms.

The present study has demonstrated a unimodal subpopulation distribution of SP and CGRP in NP and LP offspring, which skewed towards intermediate and large diameter cells. This skewed distribution and differences in subcellular staining showed that DRG neurons consist of various subpopulations. Significantly fewer small neurons were SP- and CGRP-positive in LP offspring compared with the NP group. On the other hand, more intermediate and large neurons were observed in NP and LP offspring. The percentage of SP- and CGRP-positive cells did not vary significantly between different DRG subpopulations in SP and NP rats. The precise relationship between primary afferent function and the neurochemical characterization of DRGs is still unclear. However, primary afferents of small and intermediate DRG neurons are important for the transmission of nociceptive, chemo, and mechanoreceptor information from the periphery to the CNS. They consist, respectively, of unmyelinated (*C*-conduction velocity) and thinly myelinated (*Aδ*-conduction velocity) fibers, which arise from a population of cells in the sensory ganglia. Our results provide evidence that distinct sensory neuronal populations have different functions, which may involve a differential number (small number of unmyelinated and thinly myelinated neuron cells) and expression of neurotransmitters in response to afferent renal stimuli in LP offspring. Based on our findings, we hypothesized that an impaired renorenal reflex activity in LP offspring may be associated with a decreased expression of SP and CGRP in DRG neurons, increasing the renal retention of sodium.

Defects in the level of SP receptors have been reported in non-neural vascular tissue and axonal membrane of hypertensive subjects [46,47]. In addition, an increased pain threshold associated with a reduction of CNS SP levels has been reported in hypertensive men and rats [48–50]. Based on these observations, we suggest that the impaired response to natriuresis associated with hypertension in LP rats is partly related to a defect in SP and CGRP synthesis and release by renal sensory neurons. Our findings support the hypothesis that impaired responsiveness of renal sensory receptors in maternal protein-restricted offspring may be related to a decreased release of SP from the renal pelvis and an altered distribution of SP and/or CGRP and their receptors in DRG neurons. These observations may provide a fresh framework for understanding the etiology of impaired symmetrical renal sensory responses commonly associated with the development of arterial hypertension in this programmed model. Although the precise mechanism for enhanced sodium retention in LP rats is still unclear, the current data suggest that changes in renal nerve activity are conducive to excessive hydroelectrolytic tubule reabsorption, which might potentiate hypertension. The present study supports the association between reduced natriuresis, reciprocal changes in renal nerve activity, and DRG neurokinin expression with the higher blood pressure in LP rats.

References

1. Ashton N. Perinatal development and adult blood pressure. Braz J Biol Res. 2000; 33: 731-740.
2. Barker DJP**.** In utero programming of chronic disease. Clinical Science. 1998; 95: 115-128.
3. Mesquita FF, Gontijo JA, Boer PA**.** Expression of renin-angiotensin system signaling compounds in maternal protein-restricted rats: effect on renal sodium excretion and blood pressure. Nephrol Dial Transplant. 2010; 25: 380-388.
4. Mesquita FF, Gontijo JA, Boer PA**.** Maternal undernutrition and the offspring kidney: from fetal to adult life. Braz J Med Biol Res. 2010; 43: 1010-1018.
5. Vaccari B, Mesquita FF, Gontijo JA, Boer PA. Fetal kidney programming by severe food restriction: effects on structure, hormonal receptor expression and urinary sodium excretion in rats. J Renin Angiotensin Aldosterone Syst. 2015; 16(1) :33-46.
6. Sene L de B, Mesquita FF, de Moraes LN, Santos DC, Carvalho R, Gontijo JA, Boer PA. Involvement of renal corpuscle microRNA expression on epithelial-to-mesenchymal transition in maternal low protein diet in adult programmed rats. PLoS One. 2013; 8(8): e71310.
7. Johns EJ, Kopp UC, DiBona GF. Neural control of renal function. Compr Physiol. 2011; 1:731-767.
8. Boer PA, Morelli JM, Figueiredo JF, Gontijo JA. Early altered renal sodium handling determined by lithium clearance in spontaneously hypertensive rats (SHR): role of renal nerves. Life Sci. 2005; 76: 1805-1815.
9. Oparil S, Sripairojthikoon W, Wyss JM. The renal afferent nerves in the pathogenesis of hypertension. Can J Physiol Pharmacol. 1987; 65:1548-1558
10. Beierwaltes WH, Arendshorst WJ, Klemmer PJ. Electrolyte and water balance in young spontaneously hypertensive rats. Hypertension. 1982; 4:908-915.
11. G.F. DiBona, Sympathetic neural control of the kidney in hypertension. Hypertension. 1992; 19: 28-35.
12. U.C. Kopp, L.A. Smith, Inhibitory renorenal reflexes: a role for substance P or other capsaicin sensitive neurons. Am J Physiol. 1991; 260: R232-R239.
13. Oparil S. The renal afferent nerves in the pathogenesis of hypertension. Can J Physiol Pharmacol. 1987; 65: 1548-1558.
14. Kopp UC, Smith LA. Inhibitory renorenal reflexes: a role for renal prostaglandins in activation of renal sensory receptors. Am J Physiol. 1991; 261: R1513-R1521.
15. Kopp UC, Olson LA, DiBona GF. Renorenal reflex responses to mechano- and chemoreceptor stimulation in the dog and rat. Am J Physiol. 1984; 246: F67-F77.
16. Moss NG. Electrophysiological characteristics of renal sensory receptors and afferent renal nerves. Miner Electrolyte Metab. 1989; 15: 59-65.
17. Niijima A. Observation on the localization of mechanoreceptors in the kidney and afferent nerve fibers in the renal nerves in the rabbit. J Physiol. 1975; 245: 81-90.
18. Ferguson M, Bell C. Ultrastructural localization and characterization of sensory nerves in the rat kidney. J Comp Neural. 1988; 247: 9-16.
19. Liu L, Barajas L. The rat nerves during development. Anat Embriol. 1993; 188: 345-361.
20. Gontijo JR, Smith LA, Kopp UC. CGRP activates renal pelvic substance P receptors by retarding substance P metabolism. Hypertension. 1999; 33: 493-498.
21. Gontijo JA, Kopp UC. Activation of renal pelvic chemoreceptors in rats: Role of calcitonin gene-related peptide receptors. Acta Physiol Scand. 1999; 166: 159-165.
22. Su HC, Wharton J, Polak JM, Mulderry PK, Ghate MA, Gibson SJ, Terenghi G, Morrison JF, Ballesta J, Bloom SR. Calcitonin gene-related peptide immunoreactivity in afferent neurons supplying the urinary tract: combined retrograde tracing and immunohistochemistry. Neuroscience. 1986; 18: 727-747.
23. Zheng F, Lawson SN. Neurokinin A in rat renal afferent neurons and in nerve fibers within smooth muscle and epithelium of rat and guinea-pig renal pelvis. Neuroscience. 1997; 76: 1245-1255.
24. Kuo DC, Oravitz JJ, Eskay R, De Groat WC. Substance P in renal afferent perikarya identified by retrograde transport of fluorescent dye. Brain Res. 1984; 323: 168-171.
25. Pennefather JN, Lecci A, Candenas ML, Patak E, Pinto FM, Maggi CA. Tachykinins and tachykinin receptors: A growing family. Life Sci. 2004; 74: 1445-1463.
26. Hershey AD, Krause JE. Molecular characterization of a functional c DNA encoding the rat substance P receptor. Science. 1990; 247: 958-962.
27. Gerard NP, Garraway LA, Eddy RL, Shows TB, Iijima H, Paquet JL, et al. Human substance P receptor (NK-1): Organization of the gene, chromosome localization, and functional expression of cDNA clones. Biochemistry. 1991; 30: 10640-10646.
28. De Koninck Y, Henry JL. Substance P-mediated slow excitatory postsynaptic potential elicited in dorsal horn neurons in vivo by noxious stimulation. Proc Natl Acad Sci USA. 1991; 88: 11344-11348.
29. Seybold VS, McCarson KE, Mermelstein PG, Groth RD, Abrahams LG. Calcitonin gene-related peptide regulates expression of neurokinin1 receptors by rat spinal neurons. J Neurosci. 2003; 23: 1816-1824.
30. Xavier F, Magalhães AMF, Gontijo JAR. Effect of inhibition of nitric oxide synthase on blood pressure and renal sodium handling in renal denervated rats. Braz J Med Biol Res. 2000; 33: 347-354.
31. Ciampone S, Borges R, de Lima IP, Mesquita FF, Cambiucci EC, Gontijo JA. Long-term exercise attenuates blood pressure responsiveness and modulates kidney angiotensin II signalling and urinary sodium excretion in SHR. J Renin Angiotensin Aldosterone Syst. 2011; 12(4): 394-403.
32. Lutaif NA, Gontijo LM, Figueiredo JF, Gontijo JA. Altered urinary sodium excretion response after central cholinergic and adrenergic stimulation of adult spontaneously hypertensive rats. J Physiol Sci. 2015; 65(3): 265-275.
33. Selkurt EE, Womack I, Dailey WN. Mechanism of natriuresis and diuresis during elevated renal arterial pressure. Am J Physiol. 1965; 209: 95-99.
34. Cowley AW Jr. Role of the renal medulla in volume and arterial pressure regulation. Am J Physiol. 1997; 273: R1-R15.
35. Kline RL. Renal nerves and experimental hypertension: evidence and controversy. Can J Physiol Pharmacol 1987; 65: 1540-1547.
36. Oparil S. The renal afferent nerves in the pathogenesis of hypertension. Can J Physiol Pharmacol. 1987; 65: 1548-1558.
37. Van Vliet BN, Hall JE, Lohmeier TE, Mizelle HL. Renal circulation. In: Bennett T & Gardiner S (Editors), Nervous Control of Blood Vessels. Harwood Academic Publishers, Philadelphia, 1994.
38. Kopp UC. Renorenal reflexes in hypertension. Hypertension. 1993; 11: 765-773.
39. DiBona GF. Neural control of the kidney: functionally specific renal sympathetic nerve fibers. Am J Physiol. 2000; 279: R1517-R1524.
40. Collis MG, DeMey C, Vanhoutte PM. Renal vascular reactivity in young hypertensive rats. Hypertension. 1980; 2: 45-52.
41. Patel KP, Kline RL, Mercer PF. Noradrenergic mechanism in the brain and peripheral organs of normotensive and spontaneously hypertensive rats at various ages. Hypertension. 1981; 3: 682-690.
42. DiBona GF. Nervous Kidney. Interaction between renal sympathetic nerves and rennin-angiotensin system in the control of renal function. Hypertension. 2000; 36: 1083-1088.
43. Rudd MA, Grippo RS, Arendshorst WJ. Acute renal denervation produces diuresis and natriuresis in young SHR but not WKy rats. Am J Physiol. 1986; 251: F655-F661.
44. Kopp UC, Smith LA, DiBona GF. Impaired renorenal reflexes in spontaneously hypertensive rats. Hypertension. 1987; 9: 69-75.
45. Egashira K, Suzuki S, Hirooka H, Kai H, Sugimachi M, Imaizumi T, Takeshita A. Impaired endothelium-dependent vasodilation of large epicardial and resistance coronary arteries in patients with essential hypertension. Hypertension. 1995; 25: 201-206.
46. Huang A, Koller A. Both nitric oxide and prostaglandin-mediated responses are impaired in skeletal arterioles of hypertensive rats. J Hypertens. 1996; 14: 887-895.
47. Quyyumi AA, Mulcahy D, Andrews NP, Husain S, Panza JA, Cannon RO. Coronary vascular nitric oxide activity in hypertension and hypercholesterolemia. Circulation. 1997; 95: 104-110.
48. Schobel HP, Ringkamp M, Behrmann A, Forster C, Schmieder RE, Handwerker HO. Hemodynamic and sympathetic nerve responses to painful stimuli in normotensive and borderline hypertensive subjects. Pain. 1996; 66: 117-124.
49. Sitsen JMA, De Jong W. Hypoalgesia in genetically hypertensive rats is absent in rats with experimental hypertension. Hypertension. 1983; 5: 185-190.
50. Virus RM, Knuepfer MM, Mcmanus DQ, Brody MJ, Gebhart GF. Capsaicin treatment in adult Wistar–Kyoto and spontaneously hypertensive rats: effects on nociceptive behavior and cardiovascular regulation. Eur J Pharmacol. 1981; 72: 209-217.

TABLE AND FIGURES

**Table 1.** Serum sodium, lithium, and potassium levels and sodium intake and body weight related to age in maternal normal protein intake (NP) offspring and maternal low-protein intake (LP) offspring compared with bilateral renal denervated NP (NP_DNx_) and LP (LP_DNx_) rats. The data represent the means ± SEM. The level of significance was set at *P ≤ 0.05 (one-way ANOVA or Student’s t-test).

**Figure 1.** Dams’ body weights (A), offspring body weight at birth (B) and (C) 1-day, 21-day, 10-week, and 16-week-old NP and LP body weight (in grams) compared with bilateral renal denervated NP and LP offspring. The results are expressed as means ± SD, means ± scatter dot plot, and median and quartile deviation. Data were analyzed using nonparametric analysis by a Kruskal–Wallis or one-way ANOVA tests with *post hoc* comparisons by Bonferroni’s contrast test. The level of significance was set at *P < 0.05.

**Figure 2.** Graphic representation of the systolic blood pressure (mmHg) time-course measured in conscious male NP (n = 8) and LP (n = 8) offspring (A) compared with gender and age-matched bilateral renal-denervated LP (n = 8) offspring (panel B *vs*. LP, n = 8) and (panel C *vs*. NP, n = 8) offspring. Values are means ± SD. *P < 0.05; (one-way ANOVA; *post hoc* Bonferroni’s contrast test).

**Figure 3.** Renal function studies using creatinine clearance (CCr, panel A), fractional sodium excretion (FENa+, panel B), proximal (FEPNa+, panel C) and post-proximal (FEPPNa+, panel D), fractional sodium excretion and fractional potassium excretion (FEK+, panel E), in male 10-week-old and 16-week-old NP and LP offspring compared with age-matched NP_DNx_ and LP_DNx_ groups (n = 10 for each group). Results are expressed as median and quartile deviation. Data were analyzed using nonparametric analysis by Kruskal–Wallis test with *post hoc* Bonferroni’s contrast test. The level of significance was set at *P ≤ 0.05, **P ≤ 0.01 or **P ≤ 0.001.

**Figure 4.** CLSM images and graphics showing the immunoreactivity for NK_1_R, SP, and CGRP in T13 DRG cells. The images show small (S), intermediate (I) and large (L) neurons surrounded by satellite cells. The receptor and neurokinins are present in both nuclear and cytosolic compartments. NK_1_R immunoreactivity was enhanced in LP (B) compared with NP (A) as shown by the histogram of quantification (C). Conversely, SP and CGRP expression was reduced in LP (E and H, respectively) compared with NP (D and G) rats. The statistical significance can be viewed in F and I. The data are reported as the means ± scatter dot plot. ∗P ≤ 0.05 vs. NP offspring (Student’s t-test).

**Figure 5.** Comparative statistical analysis of NK_1_R, SP, and CGRP expression in the nucleus and cytoplasm. The graphical representation depicts immunoreactivity in the cytosol and nucleus from small (S), intermediate (I) and large (L) neurons of T13 DRG from LP and NP rats. The data are reported as means ± scatter dot plot. ∗P ≤ 0.05 vs. NP offspring (Student’s t-test).

**Figure 6.** Percentage of DRG cell subpopulations: small (S), intermediate (I) and large (L) neurons expressing NK_1_R, SP, and CGRP in serial 7 μm thick ganglia sections. The data are reported as means ± SD. ∗P ≤ 0.05 vs. NP offspring (Student’s t-test).

**Figure 7.** Comparative expression of SP and CGRP in the renal pelvis of 16-week-old rats. The pictures show a normal distribution of these neurokinins in NP (A and D). In LP offspring no difference was observed in SP immunoreactivity (A, B and C), but CGRP immunoreactivity was significantly more intense in NP (D, E and F) compared with age-matched LP offspring. The data are reported as means ± SD. ∗P ≤ 0.05 vs. NP offspring (Student’s t-test).

*TABLE 1*

| ***Groups/Parameter*** | ***Na+ (mM)*** | ***Li+ (mM)*** | ***K+***  ***(mM)*** | ***Sodium intake (mmol/wk/100g b.w.)*** | ***Body weight***  ***Date of birth 16-wk-old*** |
| --- | --- | --- | --- | --- | --- |
| ***NP*** | 138 ± 3.6 | 85 ± 21 | 4.3 ±0.6 | 13.6 ± 2.4 | 7.44 ± 0.10 397 ± 22 |
| ***LP*** | 142 ± 4.1 | 78 ± 20 | 4.1± 0.5 | 12.7 ± 2.1 | 6.06 ± 0.07* 373 ± 48 |
| ***NP_DNx_*** | 140 ± 2.5 | 91 ± 23 | 4.1 ±0.6 | 13.2 ± 2.6 | - 385 ± 23 |
| ***LP_DNx_*** | 141 ± 3.7 | 87 ± 24 | 3.9 ±0.7 | 11.9 ± 4.9 | - 398 ± 38 |
