# Supplementary material for: Renal sodium handling and blood pressure changes in gestational protein-restricted offspring: Role of renal nerves and ganglia neurokinin expression
Source: PLoS One. 2017 Jun 20;12(6):e0179499. doi: 10.1371/journal.pone.0179499 (PMC5478103; doi:10.1371/journal.pone.0179499)
Supplement: S1 Thesis — (PDF) [file pone.0179499.s003.pdf]

UNICAMP

AUGUSTO HENRIQUE CUSTÓDIO

‘’ ESTUDO DA DENERVAÇÃO RENAL BILATERAL E DA IMUNORREATIVIDADE PARA SUBSTÂNCIA P (SP), CGRP E RECEPTOR 1 PARA NEUROCININA (NK1R) NO GÂNGLIO DA RAIZ DORSAL E PAREDE PÉLVICA RENAL NA PROLE DE RATAS SUBMETIDAS A RESTRIÇÃO PROTEICA GESTACIONAL.’’

CAMPINAS

2014

UNIVERSIDADE ESTADUAL DE CAMPINAS

Faculdade de Ciências Médicas

AUGUSTO HENRIQUE CUSTÓDIO

‘’ ESTUDO DA DENERVAÇÃO RENAL BILATERAL E DA IMUNORREATIVIDADE PARA SUBSTÂNCIA P (SP), CGRP E RECEPTOR 1 PARA NEUROCININA (NK1R) NO GÂNGLIO DA RAIZ DORSAL E PAREDE PÉLVICA RENAL NA PROLE DE RATAS SUBMETIDAS A RESTRIÇÃO PROTEICA GESTACIONAL.’’

Orientador: Prof. Dr. José Antonio Rocha Gontijo

Dissertação de Mestrado apresentada ao Programa de Pós-Graduação em Fisiopatologia Médica da Faculdade de Ciências Médicas da Universidade Estadual de Campinas para obtenção do título de Mestre em Ciências.

ESTE EXEMPLAR CORRESPONDE A VERSÃO FINAL DA DISSERTAÇÃO DE MESTRADO APRESENTADA PELO ALUNO AUGUSTO HENRIQUE CUSTÓDIO E ORIENTADO PELO PROF. DR. JOSÉ ANTONIO ROCHA GONTIJO.

Assinatura do orientador

____

CAMPINAS
2014

Ficha catalográfica
Universidade Estadual de Campinas
Biblioteca da Faculdade de Ciências Médicas
Maristella Soares dos Santos - CRB 8/8402

C969e

Custódio, Augusto Henrique, 1983-
Estudo da denervação renal bilateral e da imunorreatividade para substância P (SP), CGRP e receptor 1 para neurocinina (NK1R) no gânglio da raiz dorsal e parede pélvica renal na prole de ratas submetidas a restrição proteica gestacional / Augusto Henrique Custódio. -- Campinas, SP : [s.n.], 2014.

Orientador : Jose Antonio Rocha Gontijo.
Dissertação (Mestrado) - Universidade Estadual de Campinas, Faculdade de Ciências Médicas.

1. Desnutrição proteica. 2. Hipertensão. 3. Gânglios espinais. 4. Retardo do crescimento fetal. I. Gontijo, Jose Antonio Rocha,1956-. II. Universidade Estadual de Campinas. Faculdade de Ciências Médicas. III. Título.

Informações para Biblioteca Digital

Título em outro idioma: Study about renal denervation and immunoreactivity for substance P (SP), CGRP and neurokinin 1 receptor (NK1R) in dorsal root gangion and renal pelvic wall in the offspring of rats submitted to gestational protein restriction
Palavras-chave em inglês:
Protein malnutrition
Hypertension
Ganglia, Spinal
Fetal growth retardation
Área de concentração: Fisiopatologia Médica
Titulação: Mestre em Ciências
Banca examinadora:
Jose Antonio Rocha Gontijo [Orientador]
Wagner Jose Favaro
Eduardo Barbosa Coelho
Data de defesa: 24-06-2014
Programa de Pós-Graduação: Fisiopatologia Médica

BANCA EXAMINADORA DA DEFESA DE MESTRADO

AUGUSTO HENRIQUE CUSTÓDIO

Orientador (a) PROF(A). DR(A). JOSE ANTONIO ROCHA GONTIJO

MEMBROS:

1. **PROF(A). DR(A). JOSE ANTONIO ROCHA GONTIJO** ____________________
2. **PROF(A). DR(A). WAGNER JOSE FAVARO** ____________________
3. **PROF(A). DR(A). EDUARDO BARBOSA COELHO** ____________________

Programa de Pós-Graduação em Fisiopatologia Médica da Faculdade de Ciências Médicas da Universidade Estadual de Campinas

Data: 24 de junho de 2014

DEDICATÓRIAS

Dedicatórias:

À Deus, no qual confio e sei que está sempre me guiando pelos caminhos da vida.

À minha Família, que sempre mostrou que a humildade é o maior dos atributos que o homem pode ter.

À minha esposa, sempre ao meu lado, em todos os momentos sejam felizes ou tristes.

Há um tempo para tudo

"Tudo tem o seu tempo determinado, e há um tempo para tudo.
Há um tempo de nascer, e um tempo de morrer;
Um tempo de plantar, e um tempo de arrancar o que se plantou;
Há um tempo de matar, e um tempo de curar;
Um tempo de derrubar, e um tempo de edificar;
Há Um tempo de chorar, e um tempo de rir;
Um tempo de prantear, e um tempo de dançar;
Há Um tempo de espalhar pedras, e um tempo de ajuntar pedras;
Um tempo de abraçar, e um tempo de afastar-se de abraçar;
Há Um tempo de buscar, e um tempo de perder;
Um tempo de guardar, e um tempo de lançar fora;
Há um tempo de rasgar, e um tempo de coser;
Um tempo de estar calado, e um tempo de falar;
Há um tempo de amar, e um tempo de odiar;
Um tempo de guerra, e um tempo de paz." (Ecl. 3, 1-8)

Agradecimentos:

A Universidade Estadual de Campinas, a Faculdade de Ciências Médicas e especialmente ao Programa de Pós-Graduação em Fisiopatologia Médica à Regina e ao prof. Dr. Roger Frigério

Aos colegas do Laboratório de Metabolismo Hidrossalino: Ize, Flávia, Luiz Fernando, Noemi, Amanda, Vinícius Rodrigues, Vinicius Canale, Daniel, Silmara, Carmem Mir, Nelson, Rafael, Virgínia, Benito, Gabriel, Bárbara, Agnes, Heloísa, Daniele, Eduardo, Sônia, Jéssica, Alessandra, Andreia, Ana Letícia, Ana Tereza ao Professor José Francisco Figueiredo e tantos outros que passaram por este Laboratório sem os quais não seria possível realizar este trabalho.

A minha Brother Leticia Helena que me aguentou durante 2 anos as idas e vindas da Unicamp, Valeu mesmo!!!!

Aos técnicos do INFABIC, Mariana e Victor, pela ajuda com a coleta das imagens no microscópio Confocal, a Kamila do laboratório do professor Faria pela disponibilidade e ajuda com o Criostato.

Aos funcionários do Biotério, Sr. José, Marino, Sr. Antonio, Adriana Zaparolli. Aos funcionários e colegas do Núcleo de Medicina e Cirurgia Experimental, Cris, Erica, Jorge, William, Miguel, Ana, Benedito, Ivonete, Cida, Érica do CEMIB...

Ao CNPQ, pelo financiamento durante o mestrado, a CAPES e a FAPESP, que sempre nos apoiou nos trabalhos.

Agradeço também ao Fundo de Auxílio ao Estudo, Pesquisa e Extensão - FAEPEX pelo auxílio ponte concedido na fase final do trabalho de mestrado.

Agradecimento Especial:

Ao Professor Dr. José Antônio Rocha Gontijo

Por toda confiança e ajuda durante estes 3 anos de trabalho, por cada elogio e empolgação ao ver os resultados que me fizeram acreditar que certamente todo trabalho vale a pena.

A Professora Dra. Patricia Aline Boer

Pela ajuda no delineamento dos experimentos e principalmente pela sabedoria nas palavras que me motivaram na fase final de defesa, creio que sem estas, seria mais difícil...

Ao Professor Dr. Marcelo Cardoso de Lima

Minha eterna gratidão por ter me apresentado a Família do Laboratório de Metabolismo Hidrossalino, por toda dedicação e a ajuda nos experimentos e por ser grande exemplo de pessoa, agradeço de coração...

'Se fosse fácil achar o caminho das pedras, tantas pedras no caminho não seria ruim!'

Humberto Gessinger

RESUMO

Resumo

A programação fetal é um processo fisiológico que assegura, durante o desenvolvimento intrauterino, a adaptação para o mundo exterior. Ou seja, o organismo é “moldável” por estímulos durante sua formação e dependendo do insulto experimentado, existe a possibilidade de mudanças estruturais e funcionais que podem predispor o indivíduo a doenças na vida adulta. O modelo de restrição proteica, assim como outros modelos, leva a um “stress” gestacional e, segundo a hipótese de Barker, programa a prole ao desenvolvimento de doenças na vida adulta, dentre elas a hipertensão arterial. Os rins são órgãos fundamentais na manutenção do equilíbrio hemodinâmico. Mudanças morfológicas e neuroendócrinas nos rins levam a alterações hidroeletrolíticas frequentemente associadas à patogênese da hipertensão arterial. A gênese desta doença ainda não está bem descrita, por envolver alterações multifatoriais, dentre elas, modificações da atividade neural tanto central quanto periférica, que podem ser um indicativo da elevação pressórica em nosso modelo. A atividade simpática renal é um importante modulador da excreção dos eletrólitos e, quando alterada, promove maior ou menor retenção de sais, principalmente o sódio, podendo contribuir para a elevação da volemia e consequentemente da hipertensão arterial. Diversos neuropeptídeos estão envolvidos na atividade simpática renal e os níveis destes são um importante marcador na gênese da hipertensão. Dentre esses peptídeos estão a Substância P (SP), seu receptor NK1R e o Peptídeo Relacionado ao Gene da Calcitonina (CGRP). Nossos resultados mostraram redução na imunorreatividade de SP, CGRP e aumento do receptor 1 para neurocinina nos gânglios da raiz dorsal da prole de ratas submetidas à restrição proteica gestacional. Identificamos também a elevação dos níveis de CGRP na parede pélvica renal. Assim, acreditamos que haja alterações na neuromodulação da atividade aferente renal, o que pode ser um fator contribuinte para a manutenção do estado hipertensivo neste modelo experimental.

Palavras chave: Desnutrição fetal, Hipertensão, Gânglio da raiz dorsal

xix

ABSTRACT

Abstract

A fetal programming is a physiologic process that ensures an adaptation for external world during the intra uterine development. In this period, the organism is “moldable” by stimulus that happens during its formation, which ensures adequate phenotypes formation for different environments. Kidneys are the most important organs when it has to do with maintaining the organism hemodynamic balance and also morphological and neuroendocrine alterations, which leads to fluid and eletrolytes changes, frequently associated to arterial hypertension pathogenesis. The genesis of this disease is not well described yet. It involves multifactorial changes like the neural activity in both central as peripheral, which, in our model, may be an indicative of increased pressure. The renal sympathetic activity is an important excretion modulator of electrolytes and when amended, promotes greater or lesser retention of salts, mainly sodium, contributing to the increase in blood volume and consequently hypertension. Several neuropeptides are involved in renal sympathetic activity, and these levels are an important marker in the genesis of hypertension. Among these peptides we find substance P (SP) and its receptor NK1R, and Related Peptide Calcitonin Gene (CGRP). Our research showed reduced immunoreactivity of SP, CGRP and increased neurokinin 1 receptor in dorsal root ganglia among the offspring of rats subjected to gestational protein restriction. According to this result, we believe that there are changes in afferent renal activity neuromodulation which may be a contributing factor for maintenance of hypertension in this experimental model.

Keywords: fetal programming, Hypertension, Dorsal root ganglion

LISTA DE SIGLAS E ABREVIATURAS

µl	Microlitro
µm	Micrômetro
µmol	MicroMol
11β-HSD2	11-β-hidroxi-esteróide desidrogenase
ARNA	Atividade nervosa simpática renal Aferente<br>*Afferent renal nerve activity*
CCr	Clearance de Creatinina
CGRP	Peptídeo relacionado ao gene da calcitonina<br>*Calcitonin gene-related peptide*
DBH	Dopamina beta-hidroxilase
DNX	Animais que sofreram denervação renal bilateral
DPM	Desvio padrão da média
ERSNA	Atividade nervosa simpática renal Eferente<br>*Efferent renal simpathetic nerve activity*
FEK	Fração de excreção de Potássio
FENa	Fração de Excreção de Sódio
FEPNa	Fração de excreção proximal de sódio
FEPPNa	Fração de excreção pós-proximal de sódio
GRD	Gânglio da raiz dorsal
i.p.	Via intraperitoneal
Kg	Kilograma
LiCl	Cloreto de lítio
LP	Grupo hipoproteico (6% caseína)

mm	Milímetro
mmHg	Milímetros de mercúrio
MRs	Mecanorreceptores
NGF	Fator de crescimento do nervo
NK1R	receptor 1 para neurocinina
NP	Grupo Normoproteico (17% caseína)
NPY	Neuropeptideo Y
NTS	Núcleo do trato solitário
PBS	Tampão fosfato
QRs	Quimiorreceptores
RCIU	Restrição do crescimento intrauterino
SHAM	Animais que sofreram simulação cirurgica
SHR	Ratos espontaneamente hipertensos<br>*Spontaneously hypertensive rats*
SP	Substância P
VU	Volume urinário
WKY	Ratos Wistar-Kyoto

LISTA DE FIGURAS E TABELAS **PAG.**

Figura 1 A barreira placentária e a exposição fetal aos glicocorticóides.___32

Figura 2 Diagrama representando a distribuição dos nervos renais aferentes e eferentes.___ 36

Figura 3 Aumento da excreção urinária de sódio ipsilateral produzido pela denervação renal unilateral sendo acompanhado por uma redução da excreção urinária de sódio contralateral, devido a um aumento na ERSNA contralateral.___41

Figura 4 Esquema representativo da resposta reflexa renorenal ao estímulo de mecanoreceptores por elevação da pressão ureteral.___42

Figura 5 Esquema mostrando interação recíproca entre ERSNA e ARNA. 43

Figura 6 Delineamento do experimento 1. ___51

Figura 7 Delineamento do experimento 2.___54

Figura 8 Esquema da divisão de grupos.___55

Figura 9 Caixa de aquecimento para aferição da pressão arterial. ___57

Figura 10 Aparelho de pletismografia, animal posicionado no contentor de acrílico para aferição da pressão arterial.___57

Figura 11 Gráfico gerado pelo software do aparelho de pletismografia de cauda.___58

Figura 12 Resumo do protocolo de clearance de creatinina e de Lítio.___61

Figura 13 Imagens do protocolo do clearance de creatinina e de lítio._____61

Figura 14 Análise das imagens digitalizadas por microscopia confocal através do software Image J. Coleta de 5 imagens aleatórias dentro do GRD._____65

Figura 15 Análise das imagens digitalizadas por microscopia confocal através do software Image J. Setas indicando a presença de núcleo.____66

Figura 16 Análise das imagens digitalizadas por microscopia confocal através do software Image J. Separação das subpopulações de neurônios. Pequenos (P), Médios (M) e Grandes (G)._____66

Figura 17 Análise das imagens digitalizadas por microscopia confocal através do software Image J. Seleção de todo neurônio, média da intensidade dos pixels dentro do neurônio, incluindo núcleo, histograma da intensidade dos pixels dentro da escala (0-255).__67

Figura 18 Análise das imagens digitalizadas por microscopia confocal através do software Image J. Seleção do núcleo do neurônio, média da intensidade dos pixels deste núcleo, histograma da intensidade dos pixels dentro da escala (0-255)._____67

Figura 19 Cálculo dos pixels dentro da área selecionada para a separação da intensidade de luminescência entre o núcleo e o citoplasma.____68

Figura 20 Análise das imagens digitalizadas por microscopia confocal através do software Image J. Coleta de 5 imagens aleatórias da parede pélvica renal._____69

Figura 21 Análise das imagens digitalizadas por microscopia confocal através

do software Image J. Campo da Pelve renal de rato com 16 semanas, média da intensidade dos pixels dentro da escala (0-255) dividida pela área selecionada.____________________________69

Figura 22 Corte histológico do GRD T13 direita corado com DAPI, imagem retirada de microscópio de fluorescência no aumento de 20X. Setas indicam exemplos de núcleo de neurônios.__________________70

Figura 23 Ganho de massa (g) ao longo de três semanas de gestação nas ratas Wistar-HanUnib submetidas à dieta normoprotéica (17% - NP) e hipoproteica (6% - LP).____________________________________75

Figura 24 Massa corporal em gramas da prole (machos) de ratas Wistar-HanUnib. 1 dia pós-natal.____________________________________76

Figura 25 Massa corporal em gramas da prole de ratas Wistar- HanUnib. 21 dia pós-natal (desmame).____________________________________77

Figura 26 Efeito da nutrição materna sobre a pressão arterial sistólica da prole ao longo das semanas de vida.____________________________78

Figura 27 Efeito da nutrição materna e da denervação renal bilateral sobre a pressão arterial sistólica da prole ao longo das semanas de vida. NP SHAM X LP SHAM.____________________________________79

Figura 28 Efeito da nutrição materna sobre a pressão arterial sistólica da prole ao longo das semanas de vida. NP SHAM X LP DNX.__________79

Figura 29 Efeito da nutrição materna e da denervação renal bilateral sobre a

pressão arterial sistólica da prole ao longo das semanas de vida. LP SHAM X LP DNX.______________________________80

Figura 30 Efeito da nutrição materna e da denervação renal bilateral sobre a pressão arterial sistólica da prole ao longo das semanas de vida. NP DNX X LP DNX.______________________________80

Figura 31 Efeito da nutrição materna e da denervação renal bilateral sobre a pressão arterial sistólica da prole ao longo das semanas de vida. NP SHAM; LP SHAM; NP DNX; LP DNX.__________________81

Figura 32 Função renal na 10ª semana de vida. Experimento 1.__________82

Figura 33 Função renal na 16ª semana de vida. Experimento 1.__________83

Figura 34 Função renal na 10ª semana de vida. Experimento 2.__________85

Figura 35 Função renal na 16ª semana de vida. Experimento 2.__________86

Figura 36 Efeito da desnutrição proteica na prole de ratos sobre as subpopulações de neurônios do gânglio da raiz dorsal. Área das diferentes populações de neurônios obtidos de 3 ratos com 16 semanas de vida.______________________________87

Figura 37 Efeito da desnutrição proteica sobre as subpopulações de neurônios do gânglio da raiz dorsal. Número de neurônios obtidos de 3 ratos Np vs. LP.______________________________88

Figura 38 Análise estereológica do gânglio da raiz dorsal T13 direito. Efeito da desnutrição proteica sobre as populações de neurônios. _______88

Figura 39 Imunofluorescência de CGRP entre as populações de neurônios. Imagens obtidas por microscopia confocal e analisadas no software Image J.____ 90

Figura 40 Leitura da imunorreatividade de CGRP entre as populações de neurônios.____ 90

Figura 41 Leitura da imunorreatividade de CGRP no citoplasma das populações de neurônios. ____ 91

Figura 42 Leitura da imunorreatividade de CGRP no núcleo das populações de neurônios. ____ 91

Figura 43 Imunofluorescência de SP entre as populações de neurônios. Imagens obtidas por microscopia confocal e analisadas no software Image J.____ 92

Figura 44 Leitura da imunorreatividade de SP entre as populações de neurônios.____ 92

Figura 45 Leitura da imunorreatividade de SP no citoplasma das populações de neurônios.____ 93

Figura 46 Leitura da imunorreatividade de SP no núcleo das populações de neurônios.____ 93

Figura 47 Imunofluorescência de NK1R entre as populações de neurônios. Imagens obtidas por microscopia confocal e analisadas no software Image J.____ 94

Figura 48 Leitura da imunorreatividade de NK1R entre as populações de

neurônios. 94

Figura 49 Leitura da imunorreatividade de NK1R no citoplasma das populações de nneurônios. 95

Figura 50 Leitura da imunorreatividade de NK1R no núcleo das populações de neurônios. 95

Figura 51 Imunofluorescência de CGRP na pelve renal de ratos com 16 semanas de vida. Imagens obtidas por microscopia confocal e analisadas no software Image J. 96

Figura 52 Leitura da imunorreatividade de CGRP na pelve renal de ratos. Imagens obtidas por microscopia confocal e analisadas no software Image J. 97

Figura 53 Imunofluorescência de SP na pelve renal de ratos com 16 semanas de vida. Imagens obtidas por microscopia confocal e analisadas no software Image J. 97

Figura 54 Leitura da imunorreatividade de SP na pelve renal de ratos. Imagens obtidas por microscopia confocal e analisadas no software Image J. 98

TABELAS:

Tabela 1 Ingredientes da Ração com 17% de Proteínas (NP). 52

Tabela 2 Ingredientes da ração com 6% de proteína (LP). 53

Sumário

Resumo ix
Abstract xxi
1 INTRODUÇÃO 35
1.1 Modelos Experimentais de programação 38
1.2 Mecanismos da programação e o Papel dos glicocorticoides 39
1.3 Rins na hipertensão 44
1.4 Reflexo renorenal 50
2 JUSTIFICATIVA E OBJETIVOS 57
3 MATERIAIS E MÉTODOS 61
3.1 Animais 62
3.2 Massa corporal total 65
3.3 Aferição da Pressão Arterial Caudal (PAC) 66
3.4.1 Protocolo anestésico 69
3.4.2 Protocolo cirúrgico 69
3.5 Estudo da Função Renal: 70
3.5.1 Resumo do protocolo de Clearance de creatinina e lítio 71
3.5.2 Fórmulas para Cálculo do Clearance e de Fração de Excreção 72
3.6 Determinação das Concentrações de Sódio, Potássio, Lítio e Creatinina Urinária e Plasmática 73
3.7 Perfusão do Material 73
3.8 Imunofluorescência 74
3.9 Leitura das Imagens Digitalizadas 75
3.9.1 Coleta das imagens da pelve renal 75
3.9.2 Coleta das imagens da parede pélvica renal 78
3.10 Analise estereológica dos Gânglios da Raiz Dorsal 80
3.11 Análise estatística dos resultados 81
4 RESULTADOS 85
4.1- Controle da massa corporal das mães durante a gestação. 85
4.2- Controle da massa corporal da prole de machos nascidos de mães submetidas a dieta NP e LP durante a gestação. 86
4.3- Resultados da aferição da pressão arterial caudal (P.A.C.) 87
4.4- Estudo da função renal 91

4.5- Avaliação morfométrica e estereológica dos neurônios do gânglio da raiz dorsal. 97
4.6- Estudo por microscopia confocal a laser de SP, CGRP e NK1R nos neurônios do GRD. 99
4.7- Estudo por microscopia confocal a laser de SP e CGRP na parede pélvica renal de ratos com 16 semanas de vida. 106
5 DISCUSSÃO 111
6 CONCLUSÃO: 125
7 REFERÊNCIAS BIBLIOGRÁFICAS 128

1 INTRODUÇÃO

O desenvolvimento de algumas doenças é tradicionalmente relacionado ao estilo de vida como sedentarismo, má nutrição e tabagismo, dentre outros. Entretanto, embora estes fatores sejam preponderantes, diversos estudos em modelos animais e humanos têm demonstrado que fatores ambientais como desequilíbrio na nutrição materna durante a gestação podem ter efeitos adversos sobre o metabolismo dos descendentes, predispondo-os a doenças como diabetes tipo II, obesidade, doenças cardiovasculares e hipertensão arterial.

Diversos estudos têm sustentado a ideia de que alterações no ambiente intrauterino, ocorridas durante períodos específicos do desenvolvimento embrionário ou crescimento fetal, podem acarretar alterações “adaptativas” de longo prazo na fisiologia e/ou morfologia de determinados órgãos e sistemas. (ASHTON, 2000). Grande parte das informações que temos em humanos a respeito dos efeitos em longo prazo na prole de mães submetidas a perturbações nutricionais e emocionais durante a gravidez, foram obtidas a partir de estudos retrospectivos.

As primeiras evidências de “programação” foram relatadas por Rose em 1964, quando este observou que havia maior prevalência de isquemia cardíaca em indivíduos cujos irmãos foram abortados ou morreram na infância. Posteriormente em 1967, Forsdahl, usando a mesma população, correlacionou a mortalidade por doença arteroscleróticas e cerebrovasculares em pessoas de meia idade, (1960) com as altas taxas de mortalidade infantil (1896-1925), sugerindo que um ambiente pobre durante os períodos iniciais da vida poderia pré-dispor o individuo ao desenvolvimento de doenças graves futuras e que a melhora nas condições de vida pós-natal, aumentaria ainda mais este fator de risco. A teoria de que o meio intrauterino adverso pode ter efeito de longo prazo na saúde do adulto surgiu através de observações feitas por Barker e colaboradores no final da década de 1980, onde neonatos com baixo peso foram mais propensos ao desenvolvimento de doenças cardiovasculares quando adultos, sustentando a visão de que eventos ocorridos no período pré-natal, principalmente envolvendo a

desnutrição, programa o feto a desenvolver uma série de doenças crônicas. Em 1991, Alan Lucas correlacionou a importância da nutrição balanceada nos períodos gestacionais à saúde dos descendentes, sendo o primeiro a utilizar o termo “programação fetal”, que também é conhecido como “Hipótese de Barker”. Sendo assim, estes estudos epidemiológicos têm mostrado que filhos expostos a dieta pobre *in uteri* têm maior risco de desenvolver doenças cardiovasculares e diabetes tipo 2. (HALES, 1991; GLUCKMAN, 2004)

Em resposta a essas descobertas, tanto em estudos com humanos quanto com animais, foi criada uma sociedade científica, denominada DOHaD (*Developmental Origins of Health and Disease* – Origens do Desenvolvimento da Saúde e Doença), que investiga a hipótese de que o feto pode adaptar seu próprio desenvolvimento em resposta direta ao ambiente uterino, mediado por fatores como nutrição e fisiologia materna (GLUCKMAN e HANSON, 2004; McMILEEN e ROBINSON, 2005; HANSON e GLUCKMAN, 2008).

As alterações no ambiente materno são sinalizadas para o feto principalmente pela via hormonal e diversos tecidos específicos acabam mudando sua trajetória de desenvolvimento. Isso promove alterações na sua organização e no seu funcionamento, uma vez que diferentes células e tecidos são sensíveis a vários fatores em diversos momentos. Assim, os efeitos da adversidade sobre a biologia de um tecido animal depende tanto do tempo quanto do estímulo experimentado. Portanto, seja pela desnutrição, estresse ou exposição a glicocorticóides, principal mediador hormonal de estresse durante o período embriofetal, há efeitos permanentes sobre o metabolismo cardiovascular, neuroendócrino e cognitivo da prole (MEANEY *et al*., 2007).

Atualmente, o conceito de programação no início da vida está mais claro e acredita-se que as alterações apresentem vantagens adaptativas darwinianas, uma vez que a mãe pode transmitir à prole em desenvolvimento sinais de que o ambiente em que ela vive naquele momento é adverso, seja pela redução da disponibilidade de alimentos, ou por aumento do estresse causado por predação, guerras, etc. Essas mudanças aparentemente podem assegurar a sobrevivência até a fase de reprodução, garantindo que esta ocorra. Entretanto, no longo prazo

essas alterações acabam se tornando desvantajosas no período pós-reprodutivo. (GLUCKMAN E HANSON, 2004). Também se acredita que os animais expostos a um ambiente pós-natal diferente daquele pelo qual foi programado, por exemplo, nos modelos de subnutrição que acabam gerando um “fenótipo poupador” e em seguida exposição a um ambiente com alimento em abundância, ocorre discordância entre a ingestão de nutrientes e os processos homeostáticos que regulam o consumo de energia, aumentando a predisposição para doença cardiovascular e metabólica no animal adulto. (MCMILLEN & ROBINSON, 2005 ; HANSON & GLUCKMAN, 2008).

A associação entre o peso reduzido ao nascer e o risco de desenvolvimento de doença cardiovascular na idade adulta já foi relatada em populações de vários países. Atualmente, o baixo peso ao nascer é correlacionado ao risco aumentado de doença arterial coronariana e hipertensão. No entanto, nem sempre houve aceitação total desses dados, sendo a principal crítica à hipótese de Barker, variáveis como pressão arterial materna, tabagismo e nível sócio-econômico da época que não foram adequadamente controlados para as análises. Os dados que deveriam ser explicados por Barker, descrevem o efeito da privação de alimentos na gravidez de humanos em Amsterdã, na Holanda, durante a segunda grande guerra entre os períodos de 1944-1945. Durante esta fome severa, a ingestão de calorias das mães estava abaixo de 1500 cal/dia, sendo a ingestão normal em torno de 2.500 cal/dia. Mesmo assim, a diminuição da média do peso ao nascer foi de apenas 300g, o que equivale a apenas 0,5 do desvio padrão da população, gerando dúvidas sobre a hipótese da má nutrição e o desenvolvimento fetal. (PANETH, 1996). Ficou claro que uma maneira de resolver esse debate era olhar para modelos animais de programação por quais as variáveis são controladas, facilitando a observação dos mecanismos fisiológicos e consequências.

1.1 Modelos Experimentais de programação

Se por um lado ainda há algumas controvésias a respeito dos estudos epidemiológicos que originaram a hipótese de Barker, por outro há um conjunto crescente de trabalhos envolvendo modelos experimentais que apoiam o conceito de programação fetal. Observou-se que o principal fator que pode levar à programação fetal durante a gestação é o estresse, seja ele causado por alterações no ambiente, restrição do sono, manipulação, ruídos, exposição a possíveis predadores, alteração da dieta, fatores que fogem à rotina do animal de laboratório. O emprego destes modelos animais permitiu o estudo do desenvolvimento fetal e pós-natal. No caso da manipulação da dieta materna, temos uma resposta precisa e controlada pois, juntamente com constituintes alimentares, a utilização de modelos de roedores permite o direcionamento da desnutrição materna em diferentes períodos do desenvolvimento embrionário e/ou fetal. (LANGLEY-EVANS et al. 1996a; 1996b)

Vários modelos de subnutrição materno/fetal foram desenvolvidos, nos quais o baixo peso da prole ao nascer era um importante marcador associado também à elevação pressórica na idade adulta (PERSSON e JANSSON, 1992). A desnutrição pré-natal é um modelo muito utilizado para se estudar os efeitos da programação fetal, sendo uma das ferramentas para entender a fisiopatologia de doenças metabólicas e cardiovasculares (LANGLEY-EVANS, 1998; EDWARDS *et al.,* 2001; MCMILLEN e ROBINSON 2005).

O modelo de restrição proteica, no qual há manipulação dos teores de proteína na dieta da mãe durante a gestação, é muito utilizado para indução da programação. Langley-Evans classificou os teores de proteína na dieta materna como: restrição leve (12% de caseína), restrição moderada (9%) e restrição severa (6%). Uma dieta com baixo teor de proteínas durante a prenhez resultou filhotes com baixo peso ao nascimento e alterações no tamanho da placenta, além do crescimento desproporcional de órgãos, embora o cérebro não fosse afetado. Esses ratos apresentaram elevações da pressão arterial de 20-30 mmHg a partir da 4ª semana de vida, o que persistiu até a idade adulta. Langley-Evans

afirma que desnutrição materna antes da prenhez não produziu efeitos sobre os descendentes e que, uma semana de restrição independente da fase gestacional, já causaria alterações na prole. Entretanto, estudos recentes têm mostrado que o estresse nutricional pré-implantação já pode sinalizar como será o desenvolvimento do embrião, sendo que alterações neste ambiente poderiam ser determinantes para a vida pós-natal. (WATKINS et al. 2008)

1.2 Mecanismos da programação e o Papel dos glicocorticoides

Diversos estudos ainda devem ser feitos para compreendermos os mecanismos exatos que levam à programação. Atualmente, acredita-se em duas hipóteses, uma envolvendo os efeitos nutricionais diretos relacionados a biodisponibilidade de substrato e outra tendo como foco a exposição a elevados níveis de glicocorticóides maternos, que podem ter efeito direto e indireto sobre o desenvolvimento embriofetal. (BARKER et al, 1993a.; EDWARDS *et al.,* 1993 , MATTHEWS, 2000 e MEANEY et al. de 2007) Entretanto, essas duas teorias envolvem processos que não ocorrem isoladamente uma vez que o estresse pode promover redução da ingestão de alimentos, que por sua vez, também pode elevar os níveis de glicocorticóides maternos. Ou seja, parte desses resultados se deve à mediação hormonal, que altera o desenvolvimento de tecidos específicos em períodos críticos do desenvolvimento e levam a mudanças permanentes na secreção e sensibilidade desses tecidos aos hormônios (SECKL *et al*. 1997). Os glicocorticoides são os principais hormônios relacionados à homeostase em condições de estresse físico, psicológico ou nutricional. Muitas características da exposição excessiva a glicocorticoides sugerem seu possível papel na gênese da programação, que acarreta em desordens metabólicas e cardiovasculares. (REYNOLDS, 2013)

Na gestação normal, os níveis de glicocorticóides, tanto maternos quanto fetais, normalmente aumentam no período próximo do parto, o que está associado à ativação do eixo Hipotálamo-Pituitária-Adrenal (HPA) fetal. Para a prole, este aumento nos níveis de glicocorticoides num curto período de tempo, é importante para a maturação dos pulmões, principalmente relacionado a produção de

surfactantes, preparando o feto para a vida extrauterina (CHALLIS et al. 2001; SANDMAN, 2011). Entretanto, a exposição precoce e excessiva aos glicocorticóides pode promover a cessação da proliferação das células fetais resultando em indivíduos prematuros e com baixo peso (REYNOLDS, 2013). Um exemplo é o tratamento de ratas prenhe com baixas doses de dexametasona, glicocorticoide não metabolizado pela placenta, que reduz o peso ao nascer e eleva a pressão arterial na prole adulta (BENEDIKTSSON *et al.,* 1993).

Embora os esteróides lipofílicos atravessem facilmente a placenta, os níveis de glicocorticóides fetal são mais baixos do que o nível materno. Durante o desenvolvimento fetal, a enzima 11-Beta-hidroxiesteróide-desidrogenase do tipo 2 (11β-HSD2) é altamente expressa em todos os tecidos, tanto fetais quanto placentários, funcionando como uma barreira de proteção. (EDWARDS *et al*., 1993; SECKL, 1997) Os níveis de glicocorticóides maternos são aproximadamente de cinco a dez vezes maiores quando comparados aos níveis embriofetais, e isto é atribuído em grande parte à atividade placentária da enzima 11β-HSD2, sendo esta responsável pela metabolização de 80-90% dos glicocorticoides maternos que chegam à placenta (BENEDIKTSSON et al. 1993). A maior parte do cortisol circulante no feto humano é sintetizado pela glândula supra-renal fetal, ao passo que a grande parte da cortisona circulante fetal é de origem materna. Isto ocorre devido à função biológica da 11β-HSD2 em converter o cortisol materno em sua forma inativa, a cortisona, e corticosterona em 11-desidrocorticosterona no caso dos animais (BENEDIKTSSON *et al.,* 1997)

A 11β-HSD2 é amplamente expressa em diversos tecidos embriofetais, incluindo rins, pulmões, intestino e cérebro. Esta enzima é abundante no tecido neural até o terço final da gestação quando há uma rápida redução coincidindo com a fase terminal da neurogênese em humanos. Estes níveis de 11β-HSD2 sugerem que a enzima proteja o cérebro nas fases iniciais reduzindo a ativação de receptores de glicocorticóides (GRs) e mantendo o processo mitótico cerebral ativo. Posteriormente no final da gestação, há redução da expressão do gene para 11β-HSD2 permitindo a ativação dos receptores de glicocorticóides e

mineralocorticoides que contribuem para maturação neuronal e das células da glia. (DIAZ et al . 1997)

Nos modelos de programação fetal, incluindo o de desnutrição proteica, ocorre deficiência de 11β-HSD2 placentária permitindo a exposição do feto a elevados níveis de glicocorticoides materno (figura 1), o que promove retardo no crescimento da prole geralmente relacionado à manifestação de doenças futuras (SECKL, 1997; EDWARDS *et al.,* 2001). Associação similar entre peso ao nascer e concentrações de 11β-HSD2 placentária foi confirmada em humanos, além disso, marcadores bioquímicos de exposição fetal a glicocorticoides estão relacionados com a função da 11β-HSD2 placentária próximo ao nascimento (BENEDIKTSSON *et al.,* 1997). De forma intrigante, a restrição proteica durante a gestação atenua seletivamente a 11β-HSD2, porém não há relação com outras enzimas da placenta (LANGLEY-EVANS *et al.,* 1996).

Atualmente, existem relatos de alterações epigenéticas na expressão gênica após mudanças nutricionais no período pré-natal. Os mecanismos envolvem metilação e acetilação do DNA promovendo tanto silenciamento quanto ativação de genes específicos. Por exemplo, foi relatado que há alterações na expressão dos receptores da angiotensina II (AT2) devido a restrição proteica durante a gestação (MCMULLEN *et al* . 2,004), e os processos que levam a esta alteração e as suas consequências para o controle da pressão arterial e da função renal ainda estão sendo exploradas. Não está claro se as mudanças na expressão gênica são relacionadas ao remodelamento tecidual, efeitos dos glicocorticóides ou até mesmo silenciamento epigenético. No caso dos receptores angiotensina II, sabe-se que a sua expressão é controlada por (MCMULLEN e LANGLEY-EVANS, 2005) e os níveis desta expressão determinam o desenvolvimento de estruturas especializadas do rim. A situação é extremamente complexa e estudos detalhados são necessários que considerar fatores como o momento do alteração nutricional e do estágio ontológico em que são recolhidos os tecidos para a avaliação da expressão.

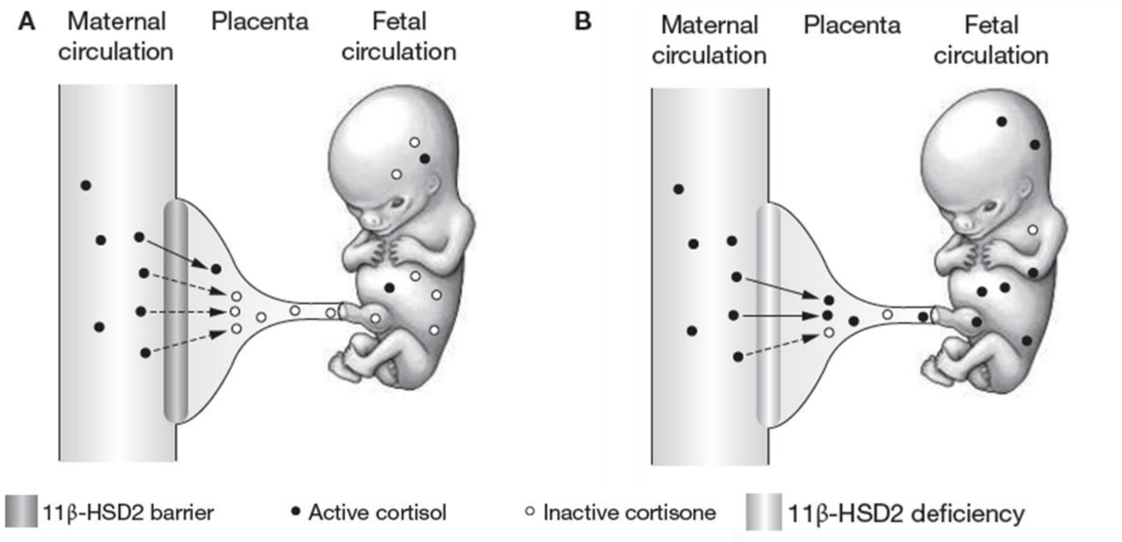

Figura 1. A barreira placentária e exposição fetal aos glicocorticóides. (A) Situação em uma placenta normal, a 11β-HSD2 age como uma barreira, pois rapidamente inativa glicocorticóides fisiológicos ativos (cortisol) para sua forma inerte (cortisona). (B) Deficiência de 11β-HSD2 permitindo a exposição do feto a elevados níveis de glicocorticoides maternos. Abreviatura: 11β-HSD2, 11β-hidroxiesteróide desidrogenase tipo 2. (ADAPTADO SECKL 2007)

O cérebro é extremamente sensível à programação pré-natal e diversos agentes como fatores de crescimento, de transcrição e nutrientes podem afetar permanentemente o desenvolvimento neural (Welberg e Seckl, 2001). Elevados níveis de glicocorticóides tem alto impacto sobre a estrutura do cérebro durante o desenvolvimento, o que pode promover alterações na atividade dos neurotransmissores e na plasticidade sináptica, resultando em mudanças que variam de sutis à drásticas, repercutindo em funções como o comportamento e cognição, bem como a susceptibilidade à doença na vida adulta (Weinstock 2008).

Em ratos, existem evidências de que a função pós-natal do eixo HPA pode ser modificada por eventos pré-natais. Tais alterações podem ocasionar, no adulto, exposições elevadas a glicocorticóides (GC) bem como respostas exacerbadas a estímulos estressantes. Sabe-se que a ativação do eixo HPA e as catecolaminas simpáticas mobilizam substratos energéticos durante períodos específicos de demanda metabólica e aumentam o fluxo sanguíneo para órgãos

vitais. Estas alterações são importantes na função do SNC, pois aumentam o processamento cognitivo e de memória a estímulos estressores, contribuindo para a capacidade do animal à respostas de fuga. As mudanças do eixo HPA frente ao estresse parecem ser vantajosas, entretanto há custos óbvios para isto, hiperglicemia, hiperlipidemia, aumento da pressão arterial, armazenamento de gordura, etc. (MEANEY et al. de 2007)

Os efeitos da programação sobre no eixo HPA envolvem alterações na expressão dos receptores de glicocorticóides (GRs) e mineralocorticoides (MRs) em regiões do cérebro, especialmente no hipocampo, responsável pela regulação da expressão do hormônio liberador de corticotrofina (CRF) pelo hipotálamo através de *feedback* negativo. No rato, a exposição a glicocorticóides durante a vida embriofetal leva a redução de GRs e MRs no hipocampo, o que a longo prazo, eleva os níveis basais de glicocorticóides, ACTH, e catecolaminas devido a falha no feedback negativo além de redução da degradação do cortisol/corticosterona circulantes em respostas ao estresse. (LANGLEY-EVANS, 1996; WEINSTOCK et al. de 1998).

Além do SNC, a formação dos rins também é afetada pela dieta materna durante a gravidez. Em ratos a restrição moderada de proteínas durante a gestação está associada a redução do número de glomérulos na prole e consequentemente ocorre diminuição da área de filtração do glomerular resultando em menor excreção de sódio e aumento da pressão arterial sistêmica, além de hipertensão e esclerose glomerulares, que podem comprometer mais nefros (LANGLEY-EVANS *et al*. 1999; WOODS *et al*. 2004).

Em estudos recentes, nosso grupo mostrou que a prole de machos cujas mães foram submetidas à restrição proteica severa ou calórica gestacional apresentou redução na expressão renal de receptores de angiotensina II desde o 12º dia de vida, período correspondente ao final da nefrogênese em ratos. Foi observado redução no número de nefros (28,4%) e aumento do volume dos tufos glomerulares (24%), quando comparados com a prole de ratas que receberam dieta normal durante a gestação. (MESQUITA *et al.,* 2010; VACCARI *et al.,* 2010). Esta redução no número de nefros corrobora os achados de outros grupos em

modelos experimentais (ROSTAND, 2003; LUYCKX e BRENNER, 2005; HOPPE *et al.,* 2007). Os nefros se desenvolvem por um balanço delicado e contínuo entre mitose e diferenciação de suas células progenitoras (NISHINAKAMURA *et al.,*2011). Entretanto, a maturação ocorre em detrimento da proliferação celular. Desta forma é plausível supor que a exposição prematura aos glicocorticoides maternos poderia reduzir o número de células tronco, na fase inicial da nefrogênese, acarretando na redução expressiva de nefros como observado em nosso modelo de restrição proteica gestacional.

Além da redução da nefrogênese, a restrição proteica materna também influencia a atividade do sistema renina-angiotensina fetal e neonatal. Na prole masculina de mães que receberam restrição de proteínas durante a gestação, houve supressão do sistema renina-angiotensina (SRA) fetal / neonatal, prejudicando o desenvolvimento renal e levando a alterações permanentes na sua estrutura e função, incluindo número reduzido de nefros (WOODS *et al*. 2001), além de alterações de receptores AT1 e AT2 que podem contribuir para a hipertensão na vida adulta (MESQUITA *et al.,* 2010b; VACCARI *et al.,* 2010).

1.3 Rins na hipertensão

Embora estudos coloquem em dúvidas a hegemonia renal no processo hipertensivo e atribuam papel mais relevante às células vasculares (CROWLEY *et al.,* 2005; MENDELSOHN, 2005) ou ao sistema nervoso central (DIBONA, 1997), diversos trabalhos tem estabelecido o papel fundamental dos rins na patogênese da hipertensão arterial sendo ampliados com o conceito de que esta hipertensão na idade adulta, bem como a disfunção renal estejam relacionadas a eventos ocorridos durante o período pré-natal (LANGLEY-EVANS *et al*. 1999; WOODS *et al*. 2001).

Esta relação entre a programação fetal e doenças renais envolve mecanismos através dos quais o prejuízo na nefrogênese pode predispor à hipertensão na vida adulta (MESQUITA *et al.,* 2010). Os rins são órgãos diretamente relacionados ao desenvolvimento de hipertensão arterial devido a sua

função sobre a homeostase hidroeletrolítica. Sua relação com a gênese da hipertensão foi demonstrada em modelos de transplantes renais cruzados onde, se o doador era hipertenso o receptor também se tornava hipertenso pós-transplante (RETTIG *et al.,* 1990; GUIDI *et al.,* 1996). Sendo assim, alterações no desenvolvimento renal podem levar a defeitos no ajuste fino da excreção de eletrólitos, papel fundamental da inervação simpática renal. Atualmente, sabemos que a inervação renal é um dos principais elos entre os rins e o sistema nervoso central, tendo ação fundamental sobre todos os aspectos funcionais renais.

O sistema nervoso simpático (SNS) tem grande participação sobre a capacidade renal em regular a pressão arterial e vice-versa, sendo que o rim tem efeito importante sobre o tônus simpático global. Em resposta a múltiplos estímulos centrais e periféricos, a atividade nervosa simpática renal é alterada no sentido de converter informações, via inervações, aos principais componentes estruturais e funcionais dos rins, incluindo as células tubulares renais, o aparelho justaglomerular e vasculatura renal (BARAJAS e POWERS 1990). Este aumento da atividade nervosa simpática renal provoca ativação das bombas Na/K/ATPases, resultando na retenção de sódio e água, secreção de renina pelo aparelho justaglomerular e vasoconstrição das arteríolas renais. (WYSS *et al.,* 1999) Além disso, a liberação de renina estimula a produção de angiotensina II e mineralocorticoides, que levam a vasoconstrição e retenção de sódio e água, respectivamente. Assim, o SNS renal promove elevação da pressão arterial por um efeito direto dependente da retenção de sal e água pelos rins. Nestes processos, parece haver também uma resposta dependente da frequência do sinal simpático assim, nos estímulos de baixa frequência há alteração da secreção da renina, enquanto frequências mais elevadas promovem modificações nos padrões de reabsorção de sódio pelos túbulos e do tônus vascular renal (KOEPKE E DiBONA, 1985).

O SNS renal controla a pressão sanguínea por duas vias, uma que parte do sistema nervoso central para os rins através de uma rica rede de fibras simpáticas eferente, exclusivamente noradrenérgica localizada na adventícia das artérias renais e outra que retorna os sinais para o sistema nervoso central através de fibras simpáticas aferentes do mesmo modo, localizado na adventícia da artéria renal.

Em 1993, LIU e BARAJAS estabeleceram a distribuição da inervação renal utilizando dopamina beta-hidroxilase (DBH) e neuropeptideo Y (NPY), como marcadores de nervos simpáticos eferentes, e peptídeo relacionado ao gene da calcitonina (CGRP) junto a substância P (SP), como marcadores de fibras sensoriais aferentes (Figura 2). Com isso foi possível observar que os nervos simpáticos renais eferentes estão localizados na árvore vascular, nos túbulos renais e nas células justaglomerulares. Já os nervos aferentes tiveram sua marcação localizada predominantemente na pelve renal, porém também foi observado no tecido conjuntivo corticomedular e, em menor número, na vasculatura renal.

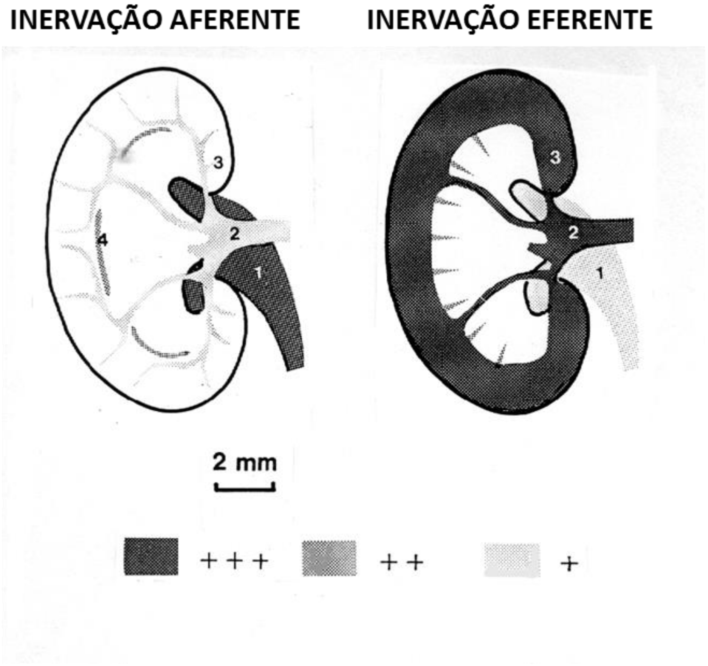

Figura 2 - Diagrama representando a distribuição dos nervos renais aferentes e eferentes. 1, Pelve renal; 2, artéria renal e suas ramificações; 3, região peri-hilar; 4, tecido conjuntivo corticomedular (LIU e BARAJAS, 1993).

Em contraste com a localização bastante distinta dos nervos sensoriais renais na parede pélvica renal, os nervos simpáticos inervam estruturas vasculares e tubulares por todo o rim, exceto na medula interna. Todas as regiões da vasculatura renal são inervadas, entretanto a maior densidade ocorre ao longo das arteríolas aferentes. Quanto às estruturas tubulares renais, a maior densidade de nervos simpáticos foi encontrada ao longo do ramo ascendente espesso da alça de Henle, túbulos contorcidos distais seguidos pelos dutos coletores e túbulos proximais. (BARAJAS e POWERS, 1990)

Fibras nervosas simpáticas eferentes também foram encontradas na parede pélvica renal, embora em menor quantidade que os nervos aferentes sensoriais, é importante ressaltar que as fibras nervosas eferentes da parede pélvica estavam em contato próximo com os nervos sensoriais. Da mesma forma, ambos foram identificados no mesmo feixe nervoso junto da parede da artéria renal. Esta estreita relação anatômica entre os nervos simpáticos e sensoriaisl está associada a interação funcional entre a atividade nervosa simpática renal eferente (ERSNA - *efferent renal sympathetic nerve activity*) e a atividade nervosa renal aferente (ARNA - afferent renal nerve activity) onde a ARNA tem papel modulador sobre a ERSNA via ativação do reflexo renorenal (KOPP *et al.,* 1984). A interação recíproca entre ERSNA e ARNA serve como mecanismo regulador importante durante condições fisiológicas para manter o equilíbrio de sódio. No entanto, em condições patológicas, pode ocorrer comprometimento do controle inibitório do reflexo renorenal e resposta inadequada de ERSNA contribuindo para a retenção de sódio (DIBONA *et al.,* 1997).

Através de estudos utilizando traçadores retrogrados axonais, verificou-se que os nervos renais aferentes são projetados a partir de gânglios da raiz dorsal (GRD) ipsilaterais, localizando-se entre as vértebras T_6 a L_2 No rim direito, a maior concentração de neurônios marcados foi observada em GRDs das vértebras T_{10} e T_{11} e no rim esquerdo em GRDs das vértebras T_{12} e T_{13} (DONOVAN *et al.,* 1983; WEISS *et al.,* 1998). Nos GRDs estão localizados neurônios pequenos cujos diâmetro variam de 10µm a 25µm, médios de 25µm – 37,5µm e grandes de 37,5µm – 60µm (BOER *et al.,* 2006).

Kopp e colaboradores em 1998, fizeram uma análise detalhada das vias nervosas renais e demonstraram que a substância P e o peptídeo relacionado ao gene da calcitonina (CGRP) estão contidos dentro dos neurônios sensoriais aferentes renais. Estes são liberados em resposta ao aumento na tensão da parede pélvica ou alterações na pressão osmótica (GONTIJO e KOPP, 1999). Os nervos aferentes renais realizam uma conversa cruzada entre os dois rins através de um reflexo renorenal modulador, que contribui para o mecanismo de controle de eletrólitos mantendo a homeostase dos fluidos corporais (KOPP *et al.,* 1985). Estes estudos experimentais formam a base para o conceito de que a atividade nervosa aferente renal tem importante papel na regulação hidroeletrolítica renal. Eles também embasam a hipótese de que em rins doentes ou lesados, sua ativação reduz a atividade reflexa do nervo simpático renal eferente e, consequentemente, a pressão sanguínea.

A substância P (SP) é um peptídeo neuroativo, membro da família das taquicininas e está presente nas porções centrais e periféricas de neurônios sensoriais. Este neuropeptídeo é sintetizado nos corpos celulares de neurônios presentes nos gânglios da raiz dorsal e distribuído, via axônios, nas terminações nervosas da rede periférica. A função neurotransmissora sensorial deste neuropeptídeo foi sugerida através de evidências demonstrando concentrações maiores de SP no corno dorsal da medula espinhal, quando comparada às raízes ventrais (KREULEN e PETERS, 1986).

O corno dorsal é a primeira estação de retransmissão dos sinais aferentes primários onde a informação é integrada ao sistema nervoso central. Alguns autores sugerem que a SP tenha papel modulador das transmissões sinápticas, podendo potencializar entradas tanto excitatórias quanto inibitórias no corno dorsal da medula espinhal. Outros estudos mostraram que a concentração de SP no gânglio cervical superior é regulada pela atividade simpática(DEVANE, 2001; LARSSON e SUN, 1992), e que a presença de SP no arco aórtico, seio carotídeo, vago, gânglio nodoso e no núcleo do trato solitário enfatiza sua função neurotransmissora no reflexo químioreceptor e pressoreceptor.

No sistema nervoso, a substância P é expressa seletivamente em sistemas sensoriais e se liga preferencialmente ao receptor 1 para Neurocinina (*NK1R*), que apresenta-se amplamente distribuído no cérebro e na medula espinhal (NAKAYA *et al.,* 1994). A sensibilização, e subsequentes respostas celulares mediadas por NK_1 após exposições repetidas à SP, podem ser rapidamente perdidas, porém a ressensibilização do receptor ao neuropeptídio volta a ocorrer gradualmente. Este mecanismo de dessensibilização e ressensibilização previne o estímulo descontrolado das células. Supõe-se que tal mecanismo envolva fosforilação do receptor, levando ao desacoplamento da proteína G, subsequente endocitose e reciclagem do receptor.

Em 2006 Boer e colaboradores mostraram a presença intranuclear de NK1R colocalizado com SP e CGRP em modelos de hipertensão (SHR) sugerindo que para os receptores NK1 nucleares tenham relevância funcional é necessária uma fonte ligante que pode ser proveniente da captação de SP extracelular e/ou produção deste neuropeptídeo pelas células neurais que deveriam, neste caso, agir intracelularmente como mediadores “intácrinos”. Estes autores sugerem que alterações na expressão e localização de neuropeptídeos e NK1R, podem estar relacionados à atenuada ARNA, com conseqüente elevação da ERSNA, retenção renal de sódio e hipertensão arterial em SHR.

O Peptídeos relacionado ao gene da calcitonina (CGRP) é um neuropeptídeo amplamente distribuído no sistema nervoso central e periférico sendo colocalizado frequentemente com outros neurotransmissores clássicos. É produto do *splicing* alternativo de transcritos primários do gene da calcitonina sendo composto por 33 aminoácidos. Geralmente, CGRP está colocalizado com SP em neurônios sensoriais aferentes primários (GIBSON *et al.,* 1984; SKOFITSCH e JACOBOWITZ, 1985) e com acetilcolina em neurônios motores (TAKAMI *et al.,* 1985). As ações de CGRP são aparentemente mediadas por receptores altamente específicos ligados à adenilato ciclase. Através de estudos metabólicos verificou-se que CGRP inibe a endopeptidase de SP, ou seja, aparentemente o CGRP reduz a degradação de SP dentro e fora das terminações (LE GREVES *et al.,* 1985). Resultados demonstraram que o CGRP promove

potencialização da resposta neural aferente renal desencadeada pelo aumento da pressão ureteral ou injeção concomitante de SP + CGRP, porém este aumento na resposta é abolido pela perfusão pélvica concomitante com thiorphan (um inibidor de peptidases), sugerindo que esta resposta seja mediada pela diminuição na degradação enzimática de SP (GONTIJO *et al.,* 1999). Outro estudo sugere que a resposta reflexa renorenal à elevação da pressão pélvica seja mediada por receptores para SP e/ou CGRP (KOPP e SMITH, 1991). Entretanto, estudos utilizando bloqueadores específicos para receptores de SP (CP-96345) ou CGRP (h-$CGRP_{8\text{-}37}$), demonstraram que a resposta reflexa renorenal promovida pela ativação de mecanoreceptores é mediada apenas pela SP, uma vez que a resposta aferente neural à elevação pressórica pélvica é bloqueada por antagonistas de SP, mas não de CGRP (GONTIJO e KOPP, 1994; GONTIJO *et al.,* 1999).

1.4 Reflexo renorenal

As observações de que um reflexo originado de um rim possa afetar a função renal contralateral foram demonstrados pela primeira vez no início de 1980. Nestes trabalhos foram observados os efeitos da denervação renal unilateral (nervos eferentes mais aferentes) sobre a função renal ipsilateral e contralateral de ratos normotensos, onde houve aumento na excreção urinária de sódio ipsilateral e concomitante diminuição da excreção urinária de sódio contralateral, resultando numa excreção urinária total de sódio inalterada (ipsilateral + contralateral) (Figura 3). A queda na excreção urinária de sódio contralateral foi devido ao aumento na ERSNA contralateral, ou seja, resposta reflexa renorenal (COLINDRES, 1980; DIBONA, 1980). Estes estudos sugerem que os nervos aferentes renais exercem efeito inibidor sobre o tônus da ERSNA.

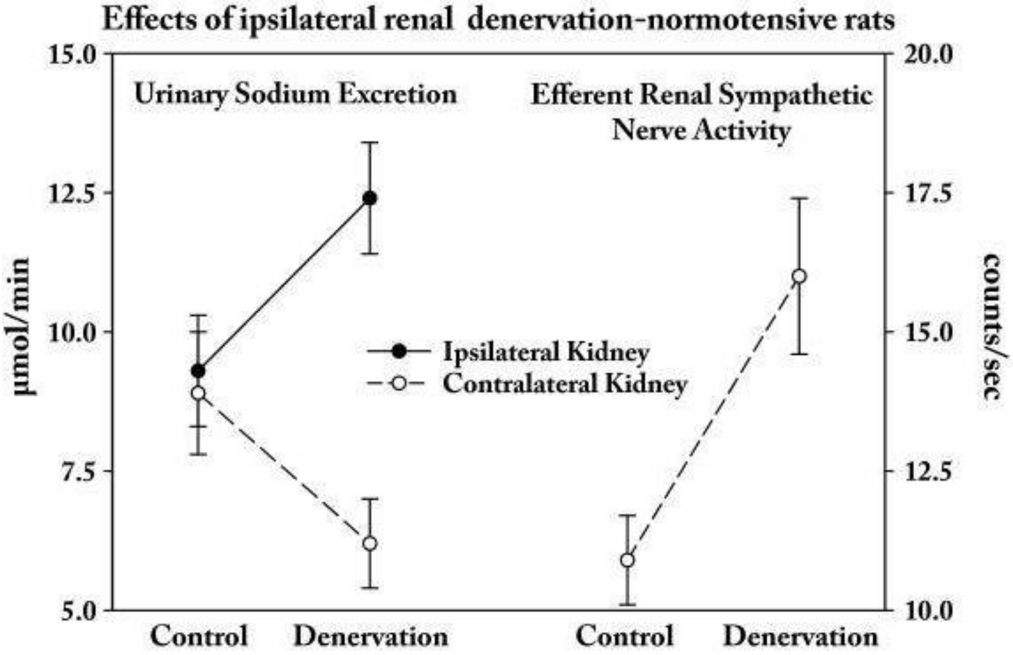

Figura 3:Aumento da excreção urinária de sódio ipsilateral (linhas contínuas) produzido pela denervação renal unilateral sendo acompanhado por uma redução da excreção urinária de sódio contralateral, devido a um aumento na ERSNA contralateral (linhas pontilhadas); mostrando o reflexo renorenal natriurético inibitório (DiBona e Kopp 1997).

Em 1984, Kopp e colaboradores definiram o reflexo renorenal como a resposta mediada por neurônios periféricos e centrais, promovida por alterações no mesmo rim (ipsilateral) ou no rim oposto (contralateral), mediada por mecanismos neuro-humorais. Este controle sobre os rins é mediado por duas classes de receptores sensoriais renais: Os mecanoreceptores (MR) que respondem ao aumento na pressão intrarenal (elevação da pressão pélvico-ureteral ou da pressão venosa) e os e quimioreceptores (CR) que respondem a isquemia e/ou a perfusão pélvica renal com soluções hipertônicas de NaCl (RECORDATI *et al.,* 1978; 1980).

Em resumo, a obstrução do fluxo urinário por exemplo, promove aumento da pressão intrapélvica renal ativando os MRs além de alterar o conteúdo de solutos medulo-papilar sensibilizando os CRs, assim há aumento ipsilateral da ARNA e este induz à diminuição da ERSNA contralateral elevando a diurese e natriurese contralateral. (Figura 4) (KOPP et. al. 1984 KOPP e SMITH, 1991a; 1991b).

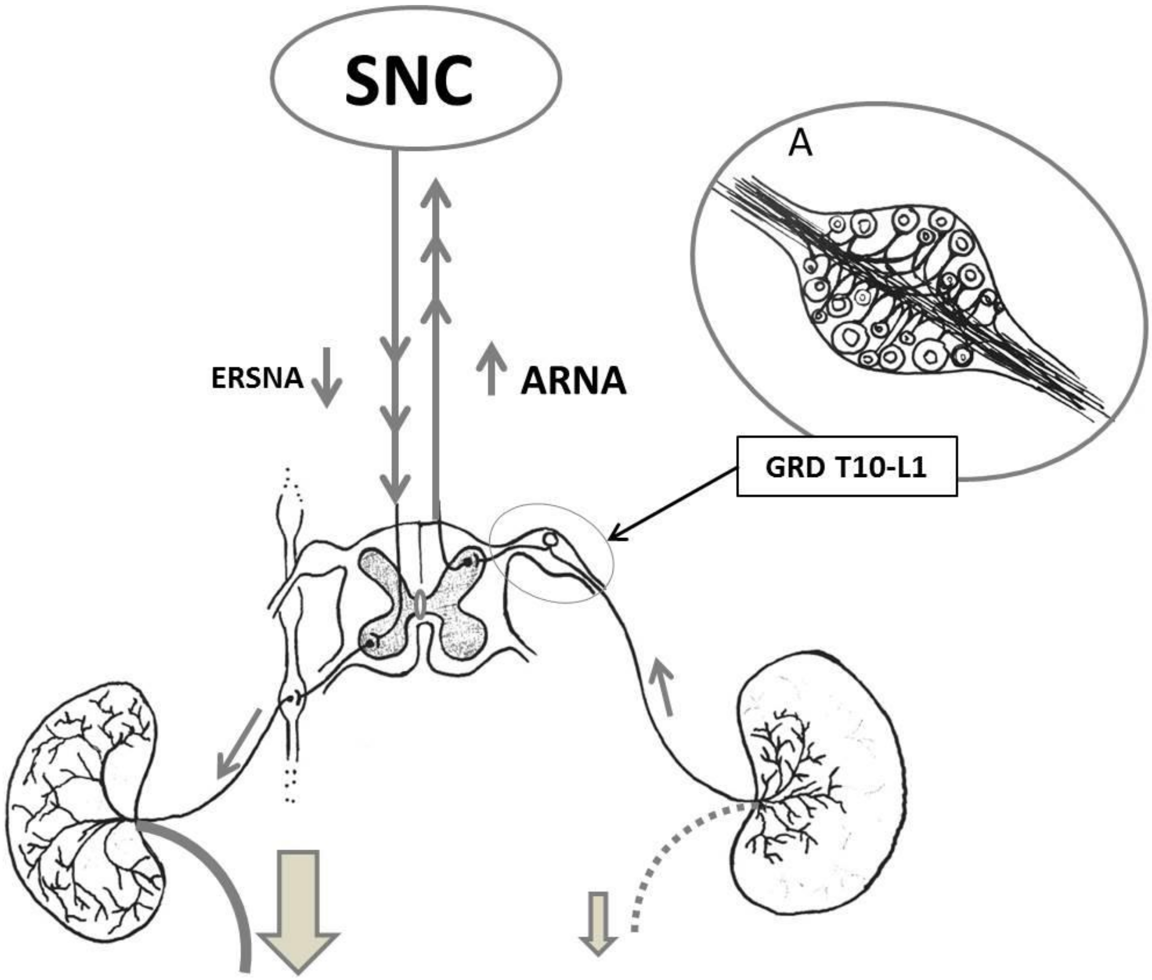

Figura 4 - Esquema representativo da resposta reflexa renorenal ao estímulo de mecanoreceptores por elevação da pressão ureteral (obstrução ureteral por exemplo),este esquema também se aplica ao estímulo de quimioreceptores pélvicos renais após perfusão com NaCl 0.9M em ratos anestesiados. A maioria dos nervos renais aferentes projeta-se do gânglio da raiz dorsal (GRD) ipsilateral para regiões entre T_{10}-L_1. SNC: sistema nervoso central; ERSNA: atividade nervosa simpática renal eferente; ARNA: atividade nervosa simpatica renal aferente A: ilustração de um corte longitudinal do GRD mostrando as subpopulações de neurônios.(ADAPTADO, DiBONA e KOPP, 1997)

Recentemente, através microscopia confocal com alta resolução, observou-se que os nervos simpáticos e os nervos sensoriais são fibras nervosas separadas, porém estão interligadas (KOPP *et al.,* 2011a). Estes estudos fornecem suporte para a interação funcional entre os nervos eferentes e aferentes renais. De fato, como discutido acima, em ratos normotensos, a ativação da ARNA leva a diminuição da ERSNA com consequente aumento da natriurese, através da resposta reflexa renorenal inibitória. Porém, o aumento da ERSNA, pode levar ao aumento da ARNA, na tentativa de diminuir ERSNA pelo mecanismo de feedback negativo, mantendo esta em menor atividade (Figura 5) (KOPP *et al.,* 2007; KOPP *et al.,* 2011a;KOPP *et al.,* 2011b).

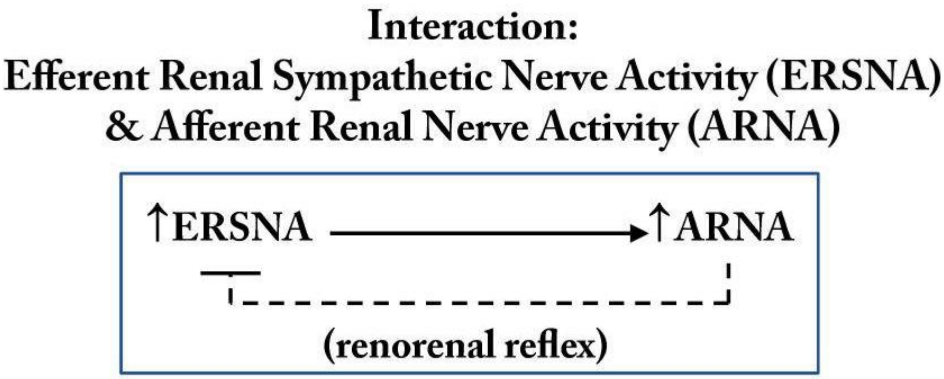

Figura 5: Esquema mostrando a interação recíproca entre ERSNA e ARNA onde o aumento de ERSNA promove aumento de ARNA, que por sua vez, tenta diminuir ERSNA via ativação do reflexo renorenal, isso ocorre como feedback negativo afim de manter baixa a ERSNA. (KOPP 2011)

Este equilíbrio entre ARNA e ERSNA ocorre frequentemente em situações fisiológicas, porém em menor grau, ele representa um mecanismo onde cada rim inibe tonicamente a atividade eferente do rim contralateral, interferindo na manipulação hidroeletrolítica renal. Assim, anormalidades neste mecanismo reflexo poderiam promover aumento na ERSNA e/ou a manutenção deste em estado elevado, tendo como consequência, maior retenção renal de sódio e água. Hipoteticamente, isto poderia ocorrer em uma situação na qual houvesse disfunção da atividade aferente sensorial renal.

Estudos em ratos SHR demonstraram que o aumento da pressão pélvica renal não produziu aumento na ARNA e o consequente reflexo renorenal contralateral (KOPP *et al.,* 1987). Posteriormente, observou-se que há alterações nos neuromoduladores sensoriais (SP, CGRP) e no receptor NK1, gerando a hipótese de que esta perda do reflexo renorenal em SHR seja, em parte, proveniente de um defeito sensorial periférico. (BOER *et. al* 2005)

Embora a redução do número de nefros seja um fator preponderante na gênese da hipertensão em animais programados, é plausível supor que assim como em SHR, o controle neural da função renal possa estar alterado. Desta forma, estudos se fazem necessários para a compreensão deste mecanismo regulador nos diversos modelos experimentais afim de elucidarmos a contribuição dos rins no processo hipertensivo.

JUSTIFICATIVA E OBJETIVOS

2 JUSTIFICATIVA E OBJETIVOS

As implicações de eventos gestacionais programando doenças metabólicas são de grande interesse para a saúde publica tanto em países desenvolvidos quanto naqueles em desenvolvimento. A adaptação às alterações ocorridas durante períodos críticos do desenvolvimento embriofetal vem se tornando um conjunto crescente de evidências sustentando a idéia de que tais alterações podem programar o indivíduo para o desenvolvimento de mudanças na morfologia e na função de determinados órgãos. Tais fatos indicaram que a deficiência nutricional durante a gestação, em geral associada à condição socioeconômica desfavorável, está associada à programação de diversas doenças bem como as doenças cardíacas e hipertensão arterial. Estes fatos têm gerado uma série de pesquisas que objetivam elucidar os mecanismos biológicos envolvidos na gênese de tal programação. Sabendo-se que em SHR, um modelo de hipertensão extensamente estudado, há um defeito na resposta renorenal que contribui para seu estado hipertensivo na vida adulta, a seguinte hipótese surgiu para nosso trabalho:

Distúrbios na neuromodulação renal, em modelos de programação fetal por restrição proteica gestacional, podem estar envolvidos na gênese e/ou manutenção da hipertensão arterial.

Assim, tivemos como objetivos específicos avaliar em prole de ratas submetidas à restrição proteica gestacional comparativamente ao controle:

1- A função renal e a pressão arterial em ratos submetidos ou não a denervação renal bilateral.
2- A imunorreatividade de Substância P (SP), do receptor 1 para neurocinina (NK1R) e do peptídeo relacionado ao gene da calcitonina (CGRP) no gânglio da raiz dorsal e na parede pélvica renal.

MATERIAIS E MÉTODOS

3 MATERIAIS E MÉTODOS

Experimento 1

Avaliação do número e tamanho dos corpos neuronais no gânglio da raiz dorsal na prole de ratas submetidas a restrição proteica gestacional;

Avaliação dos níveis de substância P (SP), Receptor 1 para neurocinina (NK1R) e do Peptídeo relacionado ao gene da calcitonina (CGRP) no Gânglio da raiz dorsal e pelve renal na prole de ratas submetidas a restrição proteica gestacional.

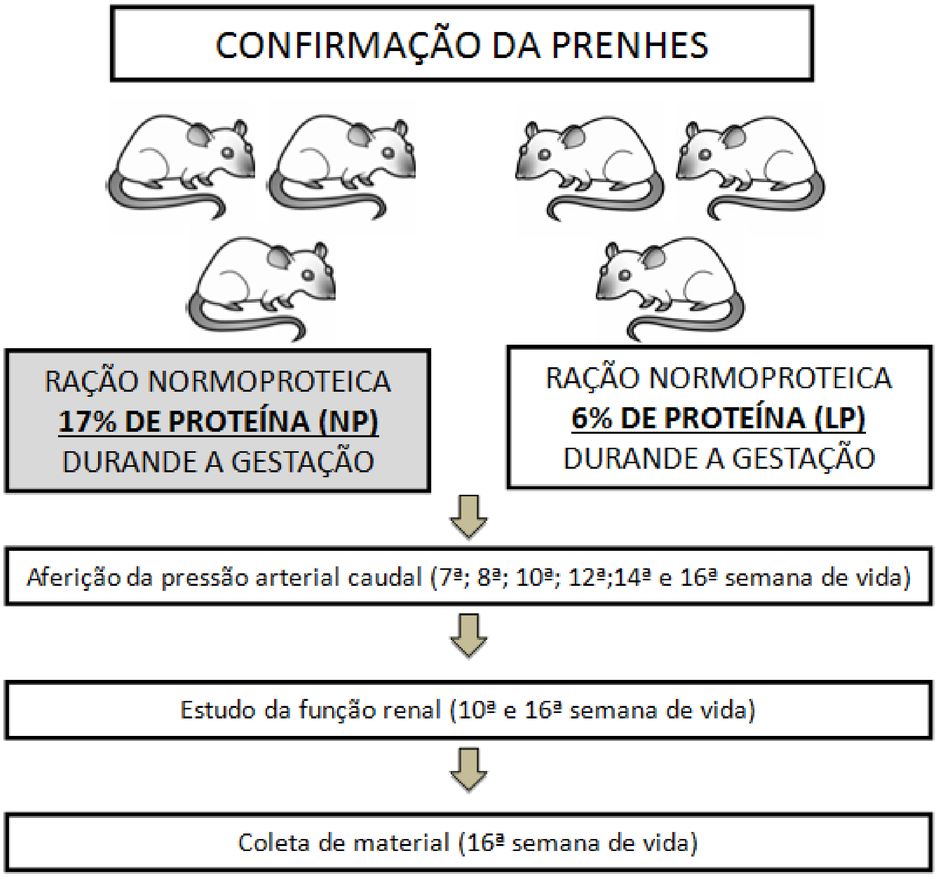

Figura 6 - Delineamento experimental do experimento 1.

3.1 Animais

Os estudos foram realizados em proles machos de ratas Wistar- HanUnib, fornecidas pelo Centro de Bioterismo da Universidade Estadual de Campinas, Campinas, SP. Durante todo experimento, os animais foram mantidos em sala com temperatura e umidade controladas com sistema de luz 12hs/12hs. Ao atingirem 12 semanas de vida, as fêmeas foram acasaladas em sistema de harem, 3:1 por 12 horas, em ambiente escuro, sendo separadas no início da manhã. A presença de espermatozóides no lavado vaginal foi utilizada como indicativo de prenhez. Após constatação da prenhez, as fêmeas passaram a ser alimentadas com dieta caseira isocalóricas e normossódicas (0,20%). As dietas foram formuladas pelo laboratório de Pâncreas Endócrino e Metabolismo (Prof. Dr. Antonio Carlos Bosqueiro) do Departamento de Fisiologia e Biofisica da Unicamp.

a) Grupo controle de acasalamento: 10 ratas receberam durante o período gestacional a dieta normoproteica (NP), 17% de proteína com as devidas especificações abaixo:

**Dieta 17% de Proteínas (NP)**					
	**INGREDIENTES**	**1kg**	**5kg**	**10kg**	**15kg**
1	Amido de milho	397	1985	3970	5955
2	Caseína (84%)	202	1010	2020	3030
3	Dextrina (90-94%)	130,5	652,5	1305	1957,5
4	Sacarose	100	500	1000	1500
5	Óleo de soja	70	350	700	1050
6	Fibra	50	250	500	750
7	Mistura de sais (AIN 93%)	35	175	350	525
8	Mistura de vitaminas (AIN 9	10	50	100	150
9	L-cistina	3	15	30	45
10	Bitartarato de colina	2,5	12,5	25	37,5

Tabela 1 - Ingredientes da Ração com 17% de Proteínas (NP)

b) Grupo experimental de acasalamento: 10 ratas receberam durante o período gestacional a dieta Hipoproteica (LP), 6% de proteínas com as devidas especificações abaixo:

**Dieta 6% de Proteína (LP)**					
	**INGREDIENTES**	**1kg**	**5kg**	**10kg**	**15kg**
1	Amido de milho	480	2400	4800	7200
2	Caseína (84%)	71,5	357,5	715	1072,5
3	Dextrina (90-94%)	159	795	1590	2385
4	Sacarose	121	605	1210	1815
5	Óleo de soja	70	350	700	1050
6	Fibra	50	250	500	750
7	Mistura de sais (AIN 93%)	35	175	350	525
8	Mistura de vitaminas (AIN 93)	10	50	100	150
9	L-cistina	1	5	10	15
10	Bitartarato de colina	2,5	12,5	25	37,5

Tabela 2 - Ingredientes da ração com 6% de proteína (LP).

Experimento 2

Estudo do efeito da denervação renal bilateral sobre a excreção urinária de sódio e a pressão arterial na prole de ratas submetidas a restrição proteica gestacional.

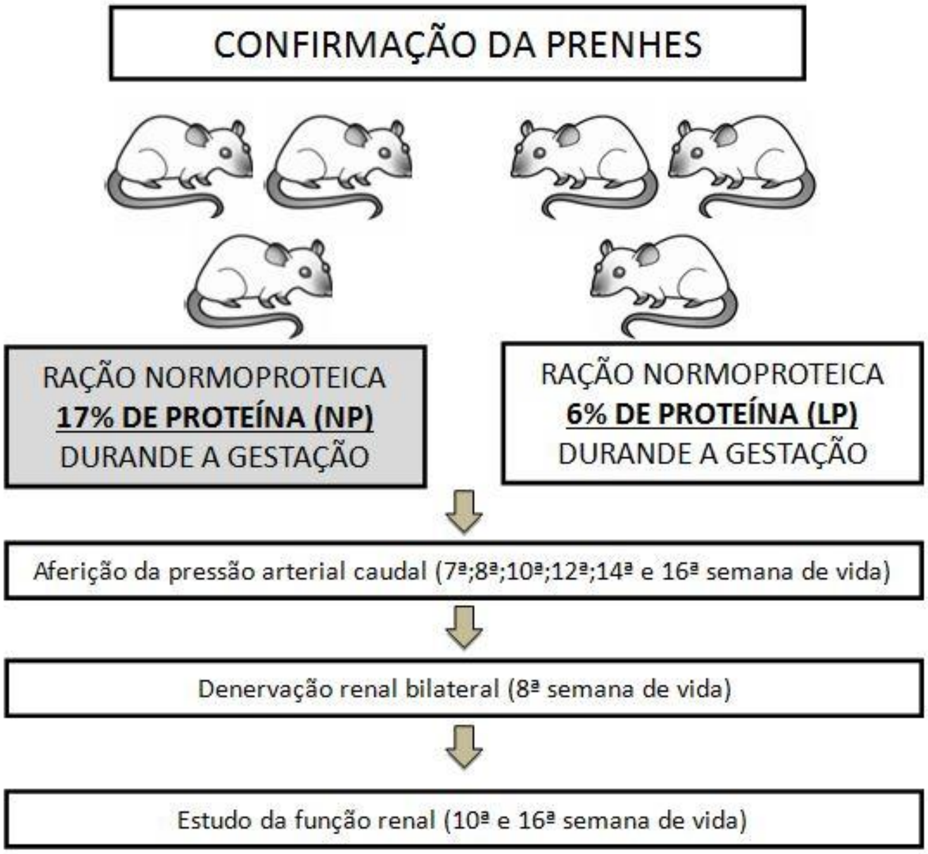

Figura 7 delineamento experimental do experimento 2.

Após o parto, durante o período lactacional, as ratas (mães) foram submetidas à alimentação de rotina, ração comercial padrão (WOODS *et al.,* 2004).

A prole macho com idade superior a 8 semanas foi subdividida em 4 grupos com 8 animais em cada:

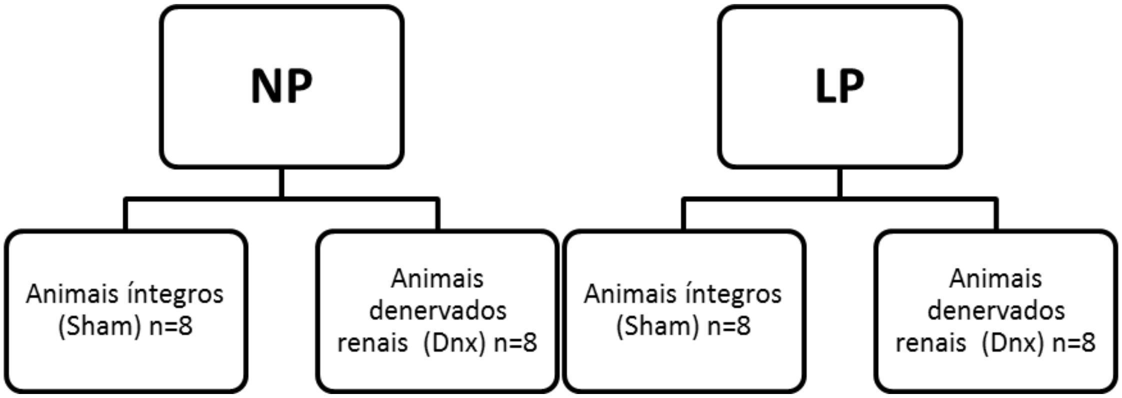

Figura 8: Esquema da divisão dos grupos.

Todos os experimentos seguiram as normas do Comitê de Ética da Universidade Estadual de Campinas, protocolo n° 2575-1. Os experimentos foram realizados no Laboratório de Metabolismo Hidrossalino do Núcleo de Medicina e Cirurgia Experimental da Universidade Estadual de Campinas.

3.2 Massa corporal total

Todos os animais (mães e prole de machos) foram pesados para acompanhamento da evolução da massa corporal ao longo do experimento. Para a aferição da massa corporal das mães, o procedimento de pesagem foi realizado durante todo o período gestacional. Já para a prole de machos, foi aferida a massa corporal total no dia do nascimento e aos 21 dias de vida, correspondente ao desmame.

3.3 Aferição da Pressão Arterial Caudal (PAC)

A pressão arterial sistêmica dos animais foi aferida de forma indireta quinzenalmente (a partir da 7ª semana). Os ratos machos foram submetidos à aferição de pressão arterial caudal (PAC), pelo método de pletismografia de cauda a partir da 7ª semana de vida (Automatic Cuff Inflation Pump, ITC Life Science Inc®), descrita previamente por Lovemberg (1987). Sempre antes de cada procedimento, o aparelho foi devidamente calibrado, sendoa a leitura do software a mesma da coluna de mercúrio. Cada animal foi colocado em uma caixa, de aquecimento por 5 minutos a 32ºC. Este procedimento é necessário para a indução da vasodilatação, muito importante para verificação da pressão arterial nesta técnica. Após o tempo de aquecimento o animal foi colocado em um tubo contentor de acrílico e um transdutor inflável (*cuff)* foi ajustado na porção proximal da cauda, este se conecta com a câmara pletismográfica o qual faz a leitura e manda os dados para o computador. A pressão na bainha pneumática, a qual está conectada a um manômetro de mercúrio, foi aumentada acima da pressão sistólica (~250 mmHg) e, então, lentamente liberada. Os resultados da pressão sistólica foram registrados pelo manômetro pletismográfico e convertido em gráficos formatados pelo software. Para cada rato a pressão arterial foi aferida pelo menos 5 vezes e a média calculada.

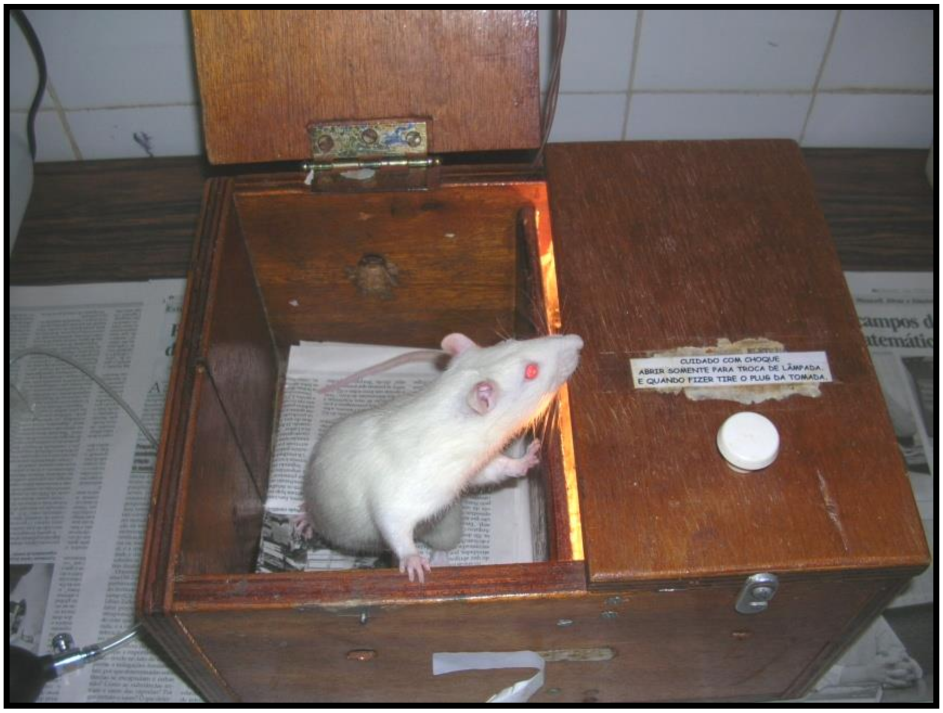

Figura 9 - Caixa de aquecimento para aferição da pressão arterial

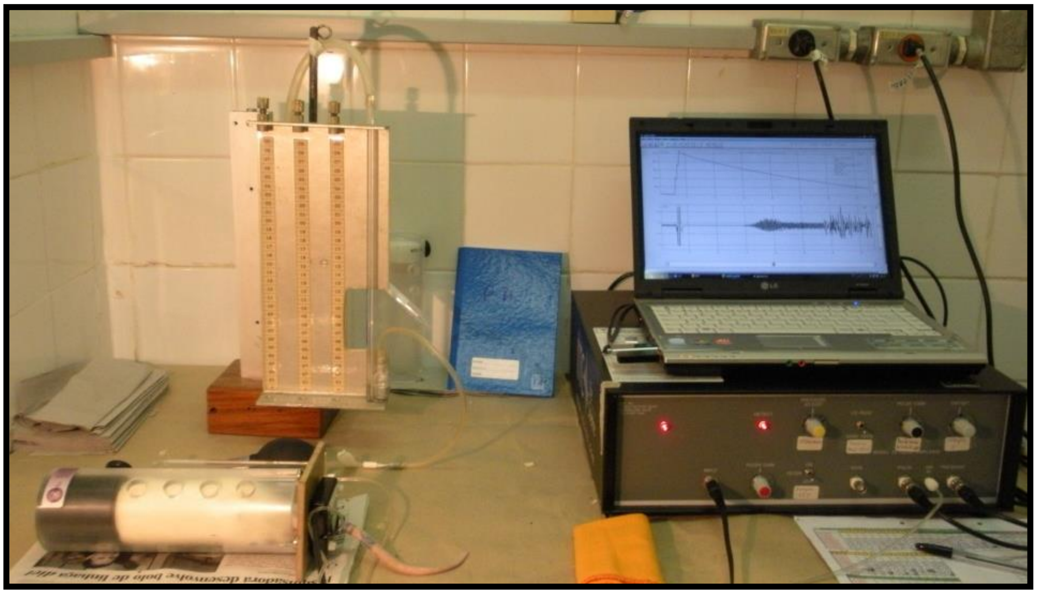

Figura 10 - Aparelho de pletismografia, animal posicionado no contentor de acrílico para aferição da pressão arterial.

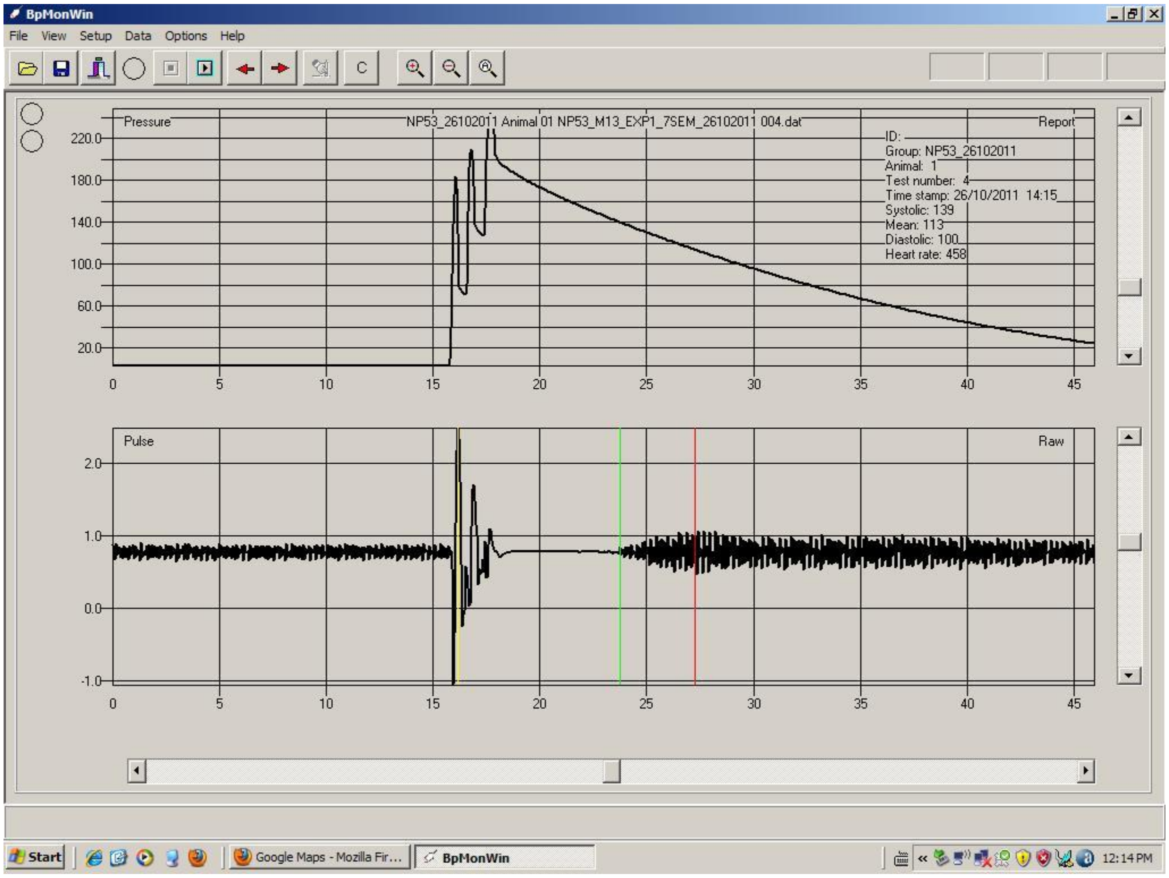

Figura 11 – Gráfico gerado pelo software do aparelho de pletismografia de cauda.

3.4 Denervação renal bilateral:

3.4.1 Protocolo anestésico

Os animais, estavam em jejum sólido pelo menos 6 horas antes do procedimento, estes foram anestesiados pela via intraperitoneal (i.p.) com administração da mistura de fármacos cloridrato de Ketamina na dose 75 mg/Kg + Cloridrato de Xilasina na dose 10 mg/Kg + sulfato de atropina na dose de 0,1 ml/100mg.

3.4.2 Protocolo cirúrgico

Após a redução dos reflexos corneais e ausência de resposta a estímulos dolorosos, foi iniciada o procedimento cirúrgico. O acesso renal foi realizado por abordagem retroperitonial (flanco). Primeiramente, incisão da pele, entre as vértebras T10 e S1 na linha média dorsal, divulsão do subcutâneo na região a ser acessada (rim esquerdo ou direito), incisão na fáscia toracolombar e divulsão dos músculos até exposição das gorduras perirenais, estas foram tracionadas delicadamente com ajuda de uma haste de algodão flexível (Cotonete®), até a exposição do rim, artéria, veia e nervos renais. Com auxílio de um estereomicroscópico Nikon® utilizando-se uma ocular de 2,5x e objetiva com ampliação de 20x, a artéria renal foi identificada e dissecada, da região de hilo renal até o ponto visível mais medial possível. Posteriormente foi realizada a retirada das fibras nervosas adjacentes a artéria renal por processo mecânico (raspagem da adventícia) com uma pinça de dissecação, e posteriormente realizado a denervação química com algodão embebido com fenol alcoólico a 10%, por toda superfície da artéria renal. A sutura foi realizada em planos musculares do tipo contínua e no plano dérmico com sutura simples interrompida. Todo o processo foi repetido para o acesso do rim contralateral. Os animais “SHAM” também tiveram seus rins manipulados, porém sem a denervação mecânica e química. Todos os animais que participaram do procedimento cirúrgico foram colocados em gaiolas individuais para o retorno da anestesia, e após retornarem da sedação, foram colocados nas gaiolas coletivas.

3.5 Estudo da Função Renal:

Para avaliação da função renal foi utilizado o método de clearances de creatinina e de lítio em animais acordados, sempre após a verificação da pressão arterial (1 ou 2 dias) para que o estresse gerado pela coleta não influencie a pressão.

Quinze horas antes do início da coleta de urina, os animais receberam uma solução de Cloreto Lítio (Synth®) na dosagem de [0,06mEq/100g] por gavagem (introdução de uma sonda gástrica), no volume de 1% do peso corporal. Os animais permaneceram em jejum sólido durante o experimento e água *ad libitum*. Uma hora e vinte minutos antes do inicio da coleta, foi administrado por gavagem uma sobrecarga hídrica (água potável) de volume igual a 10% peso corporal. Aos vinte minutos antecedentes do inicio da coleta, a mesma sobrecarga foi administrada por gavagem e os animais colocados em gaiolas metabólicas individuais. Após um intervalo de 20 minutos, iniciou-se a coleta de urina através de funis adaptados sob gaiolas metabólicas em tubos graduados de vidro, por um período de 2 horas exatas. Com o término das horas de coleta, o volume urinário foi anotado, a urina homogeneizada, centrifugada, e o sobrenadante foi congelado (-18°C) para posterior dosagem. Os animais foram anestesidados com Eter e, colocados em uma cama contentora, foram coletadas amostras de sangue (~1,5ml) em microtubos (eppendorf®) heparinizados (0,02 mL). Os microtubos com sangue, devidamente identificados, foram centrifugados a 3500 rpm por 10 minutos e o plasma foi aliquotado e congelado (-18°C) para posteriores dosagens. Após o procedimento de coleta de sangue os animais foram levados novamente ao biotério para a recuperação.

As variáveis da função renal avaliadas foram: Fluxo Urinário, clearance de creatinina e lítio, fraçãode excreção de sódio, fração de excreção proximal e pós-proximal de sódio e fração de excreção de potássio, calculadas segundo fórmulas abaixo definidas

3.5.1 Resumo do protocolo de Clearance de creatinina e lítio

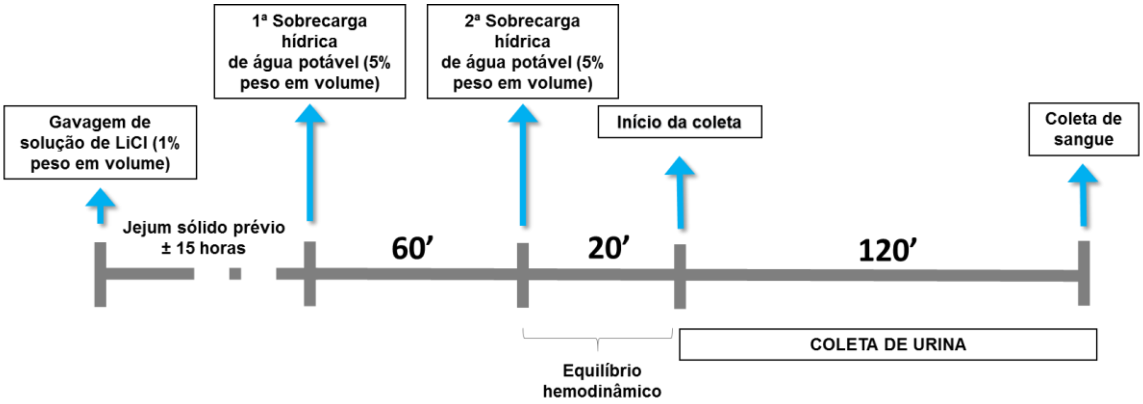

Figura 12 – Esquema do protocolo do clearance de creatinina e de lítio.

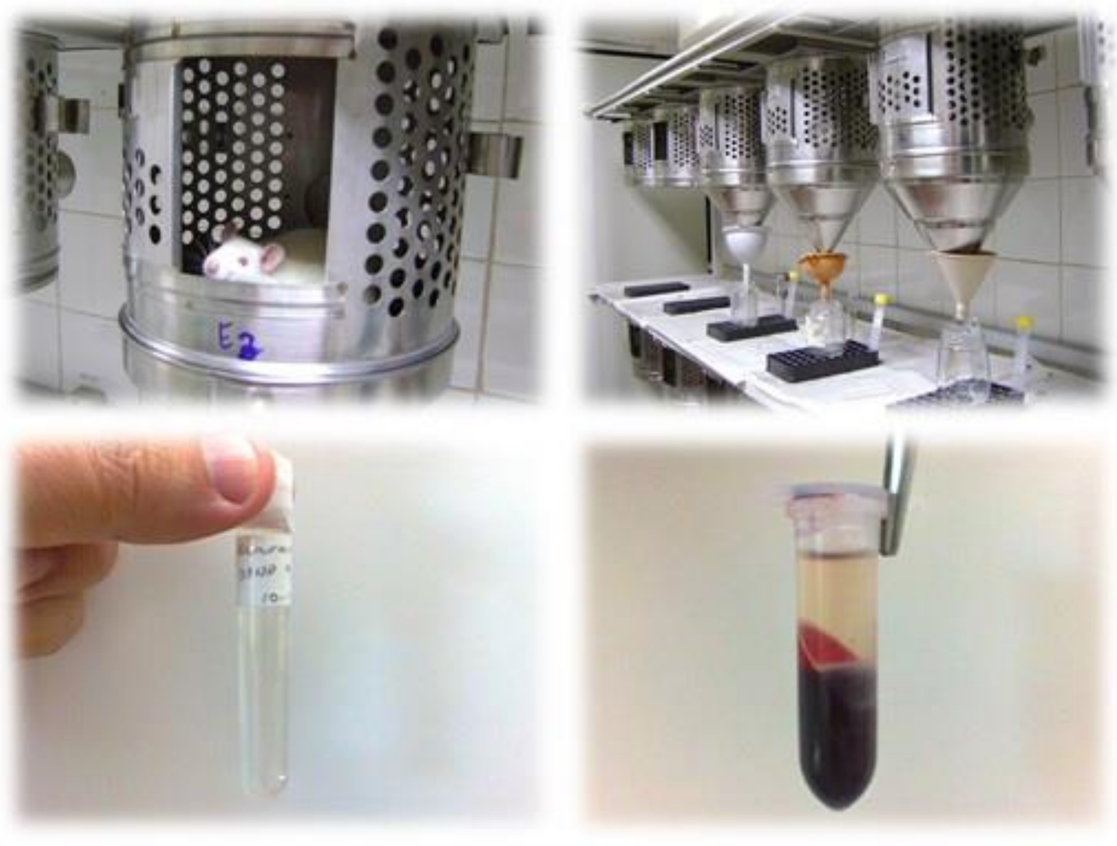

Figura 13 – Imagens do protocolo do clearance de creatinina e de lítio.

3.5.2 Fórmulas para Cálculo do Clearance e de Fração de Excreção

3.5.2.1 Clearance de Creatinina (CCr)

Representa a depuração plasmática da creatinina por unidade de tempo pela totalidade dos glomérulos renais funcionantes, calculada pela fórmula (UxV1/P), sendo U a concentração urinária de creatinina, V1 o fluxo urinário minuto e P a concetração plasmática de creatinina. Os resultados foram expressos em µl/min/100g peso corporal.

3.5.2.2 Fração de Excreção de Sódio (FENa)

Representa a fração de carga filtrada de sódio excretada pela urina num deterimnado período de tempo, calculada pela fórmula (CNa/CCr x 100), sendo CNa o clearance de sódio e o CCr, o clearance de creatinina. Os rsultados foram expressos em porcentagem (%).

3.5.2.3 Fração de excreção de Potássio (FEk)

Representa a fração de carga filtrada de postássio excretada pela urina num determinado período de tempo, calculada pela fórmula (Ck/CCr x 100), sendo Ck o clerance de potássio e o CCr, o clearance de creatinina. Os resultados foram expresso em porcentagem (%).

3.5.2.4 Fração de excreção proximal de sódio (FEPNa)

Representa a fração de carga filtrada de sódio excretada ao longo do túbulo proximal do nefro, calculada pela fórmula (CLi/CCr x 100). Sendo CLi o clearance de lítio e o CCr, o Clearance de creatinina. Os resultados foram expressos em porcentagem (%).

3.5.2.5 Fração de excreção pós-proximal de sódio (FEPPNa)

Representa a fração de carga filtrada de sódio excretada ao longo dos segmentos distais do túbulos proximal do nefro, calculada pela fórmula (CNa/CLi x 100) sendo CNa o clearance de sódio e o CLi, o clearance de lítio. Os resultados foram expressos em porcentagem (%).

3.6 Determinação das Concentrações de Sódio, Potássio, Lítio e Creatinina Urinária e Plasmática

As concentrações de sódio, potássio e lítio, no plasma e na urina, foram realizadas pelo método de fotometria de chama (B262; Micronal®, São Paulo, Brazil), enquanto a concentração de creatinina urinária e plasmática será mensurada pelo método de picrato alcalino, por espectrofotometria.

3.7 Perfusão do Material

Os ratos foram anestesiados com Cloridrato Cetamina (75mg/kg) e Cloridrato de Xilasina por via intraperitoneal, após a perda do reflexo corneal, foi feia a abertura da cavidade abdominal pela linha média ventral, ressecção do m. diafragma na junção com o gradil costal para acesso a cavidade torácica, gradil foi seccionado nas laterais e rebatido cranialmente expondo o coração. Uma agulha ligada a de uma bomba de perfusão foi introduzida no ventrículo esquerdo perfundido uma solução salina contendo heparina (2%) por 15 minutos e posteriormante por 20 minutos com uma solução de paraformaldeído (4%) em tampão fosfáto (PBS) 0,1M, pH 7.4. Após a fixação por perfusão, os Rins e GRD (T10 à L1) foram removidos imediatamente e fixados por imersão 1 hora em paraformaldeído 4%, seguido de 1 hora em PBS contendo 0,1% de glicina a temperatura ambiente e posteriormente numa solução de sacarose 15% dissolvido em PBS *overnight* a 4°C. No dia seguinte os GRD e os Rins foram incluídos em um crioprotetor (Tissue-Tech®) e congelado em CO2 (-79°C). Para a confecção das lâminas, foram feitos cortes de 7µm para os GRD e 14 µm para os rins em um

criostato (Leica®) a -22°C, tais cortes foram ambos montados em lâminas silanizadas e guardados no Biofreezer (-80°C).

3.8 Imunofluorescência

Os cortes histológicos, aderidos em lâminas silanizadas, foram lavados em PBS (0,1M, pH 7,4) por 3 vezes (5 minutos cada), incubados com solução bloqueadora (Soro não imune de Burro - 3% e Albumina bovina - 3% em PBS. Cada corte foi incubado com o anticorpo primário; goat anti -substância P 1:100, (Santa Cruz CA); rabbit anti – CGRP 1:100, (Neuromics®); rabbit anti - NK1R 1:100, (Novus Biologicals®), todos diluído em PBS + TRITON X-100 (0,3%) *overnight* sob refrigeração (4°C). No dia seguinte, foi feita lavagem com PBS (4 vezes com intervalos de 5 minutos) e os cortes foram incubados com anticorpo secundário específico, Dylight® 594 anti – rabbit para CGRP e NK1R e Cy™ 3 anti-goat para SP, diluídos em tampão PBS + TRITON X-100 (0,3%) por 2 horas à temperatura ambiente. Após lavagens sucessivas com PBS, os cortes foram montados com lamínula, utilizando-se o meio de montagem para fluorescência Vectashield® com Dapi (Vector Laboratories, Inc. Burlingame), e a fluorescência no foi detectada por microscopia de fluorescência e confocal à laser (Microscópio ZEISS® LSM780 do Instituno Nacional de Ciências e Tecnologia de Fotônica Aplicada à Biologia Celular – (INFABIC). Através das imagens digitalizadas, a imunorreatividade das amostras foram analisadas através do software científico Image J®.

3.9 Leitura das Imagens Digitalizadas

3.9.1 Coleta das imagens da pelve renal

Após a imunofluorescência com o anticorpo de interesse, no microscópio confocal, foram coletados 5 imagens aleatórias do gânglio da raiz dorsal (T13) esquerda visando o maior número de neurônios possíves dentro do campo como mostrado na figura 14. Após as coletas das imagens digitalizadas, estas foram analisadas no software Image J, onde apenas os neurônios que possuíam núcleo evidente foram analisados.(Figura 15). Os neurônios foram separados de acordo com os seus diâmetros em pequenos que variam de 10µm a 25µm, médios de 25µm – 37,5µm e grandes de 37,5µm – 60µm (Figura 16) segundo ALVARES et al. 1991; ALINE BOER 2005. Através do software, foi feita a seleção da área do neurônio (Figura 17), e obtemos um gráfico contendo a área selecionada a média da intensidade dos píxels presentes nesta área. Lembrando que em uma imagem 8 bits, cada píxel obedece uma escala de luminescência que varia de 0 a 255. Geramos também um histograma da imunorreatividade no neurônio e uma lista de cada pixel lido, metodologia importante para separarmos núcleo do citoplasma. O mesmo procedimento foi realizado para a selação do núcleo do neurônio, o qual também teve uma lista gerada (figura 18).

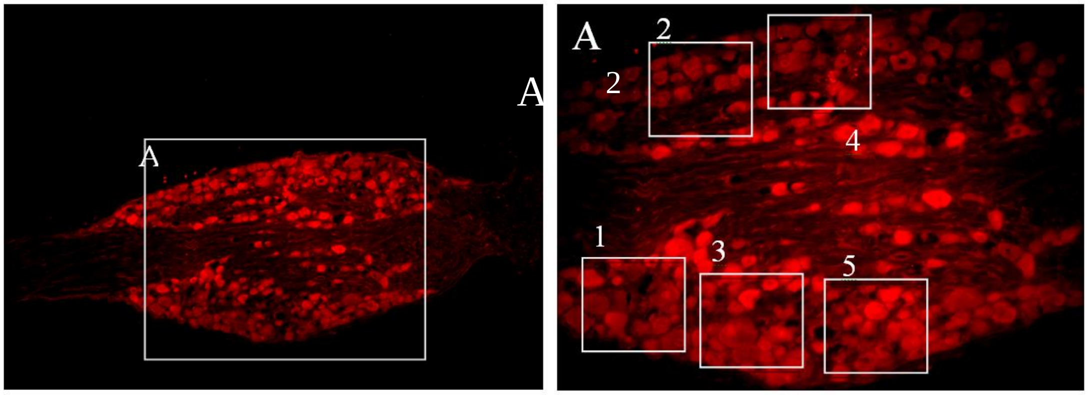

Figura 14 - Análise das imagens digitalizadas por microscopia confocal através do software Image J. Coleta de 5 imagens aleatórias dentro do GRD.

Para a separação da imunomarcação entre o citoplasma e o núcleo, foi feito a subtração da leitura do núcleo da leitura total, exemplificado na figura 14 que posteriormente por cálculos feitos no Microsoft Excel® determinamos as médias da intensidade dos píxels presentes citoplasma.

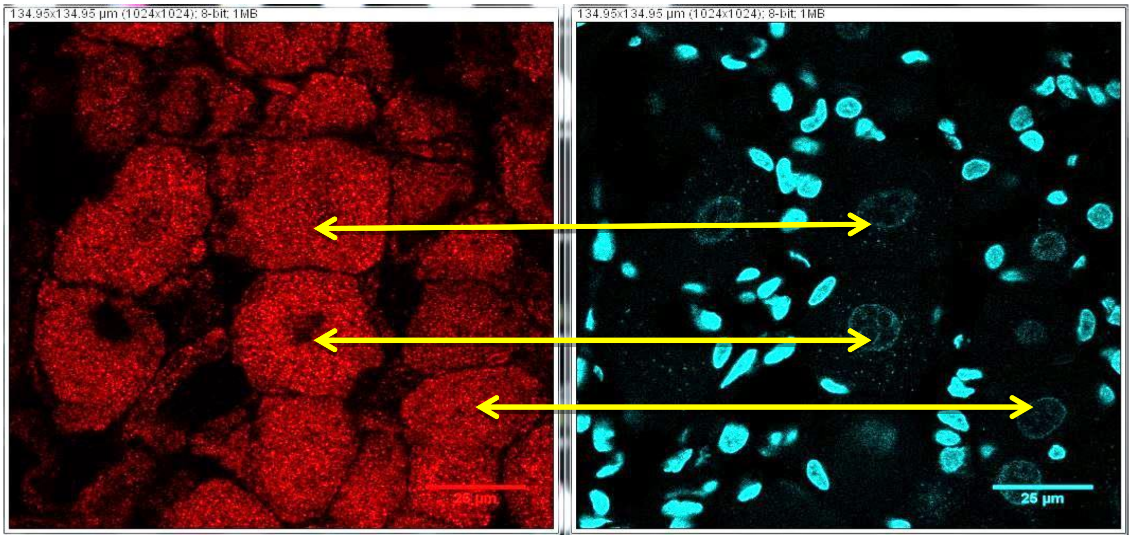

Figura 15 - Análise das imagens digitalizadas por microscopia confocal através do software Image J. Setas indicando a presença de núcleo.

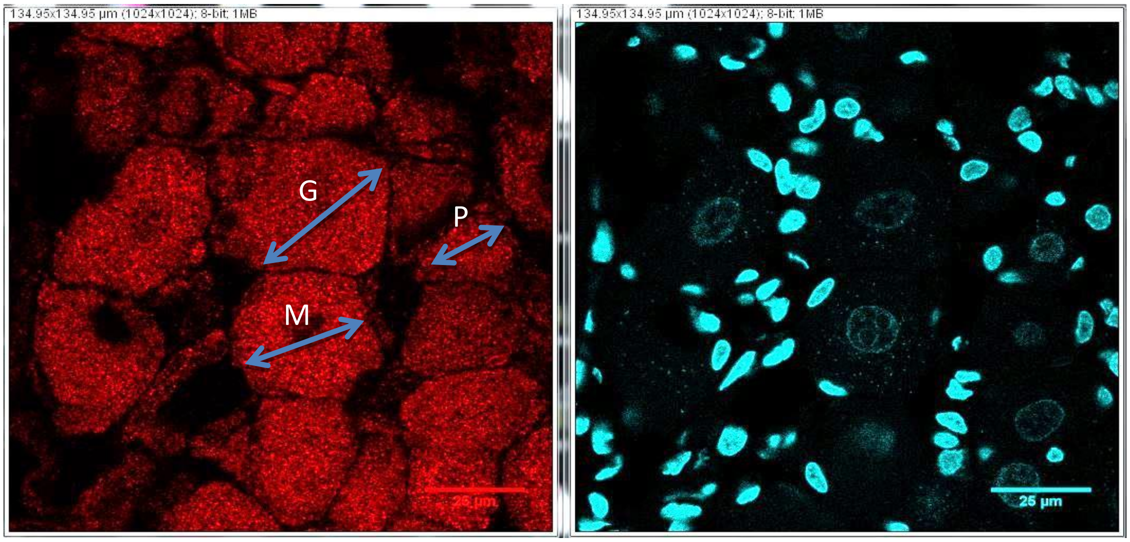

Figura 16 - Análise das imagens digitalizadas por microscopia confocal através do software Image J. Separação das subpopulações de neurônios. Pequenos (P), Médios (M) e Grandes (G).

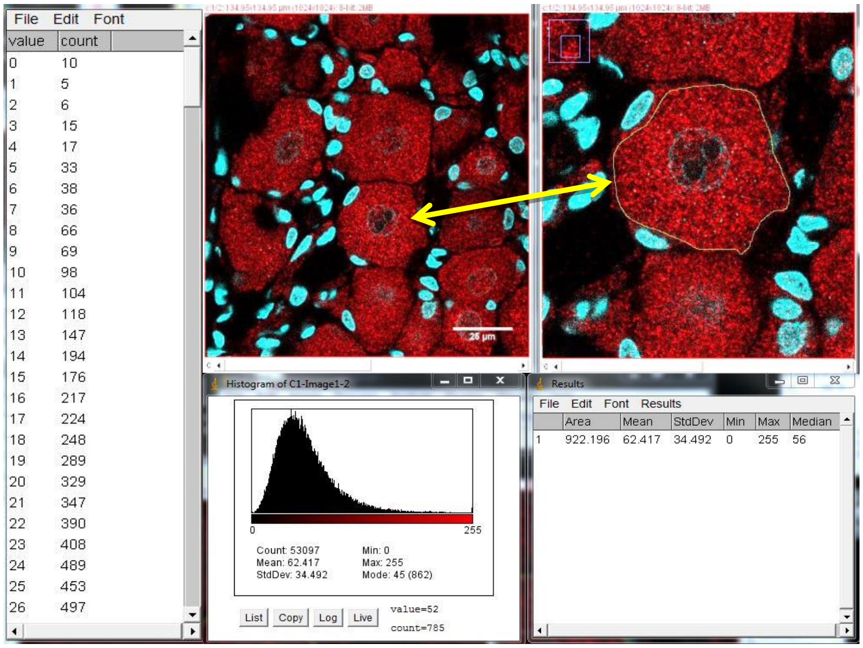

Figura 17 - Análise das imagens digitalizadas por microscopia confocal através do software Image J. Seleção de todo neurônio, média da intensidade dos pixels dentro do neurônio, incluindo núcleo, histograma da intensidade dos pixels dentro da escala (0-255).

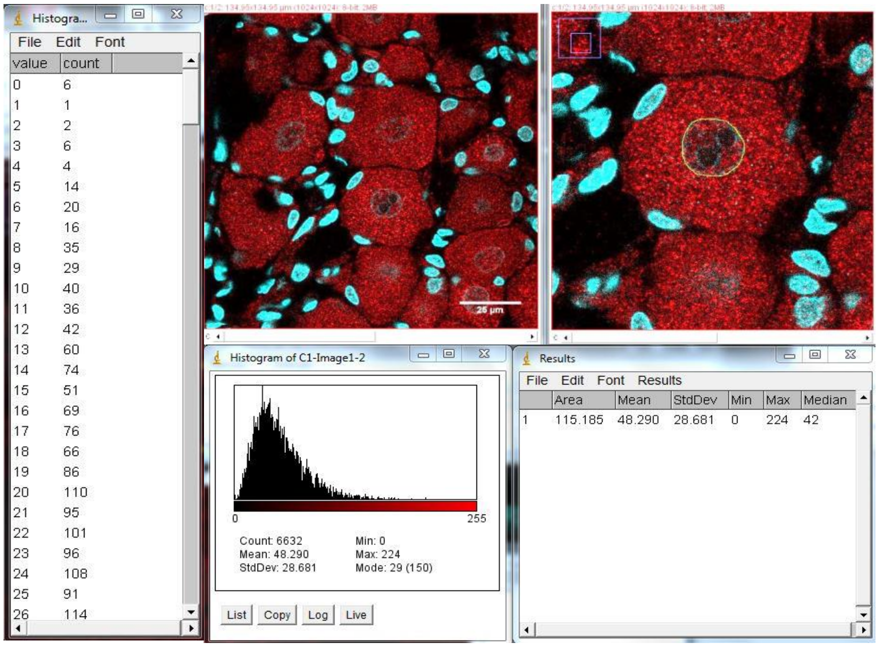

Figura 18 - Análise das imagens digitalizadas por microscopia confocal através do software Image J. Seleção do núcleo do neurônio, média da intensidade dos pixels deste núcleo, histograma da intensidade dos pixels dentro da escala (0-255).

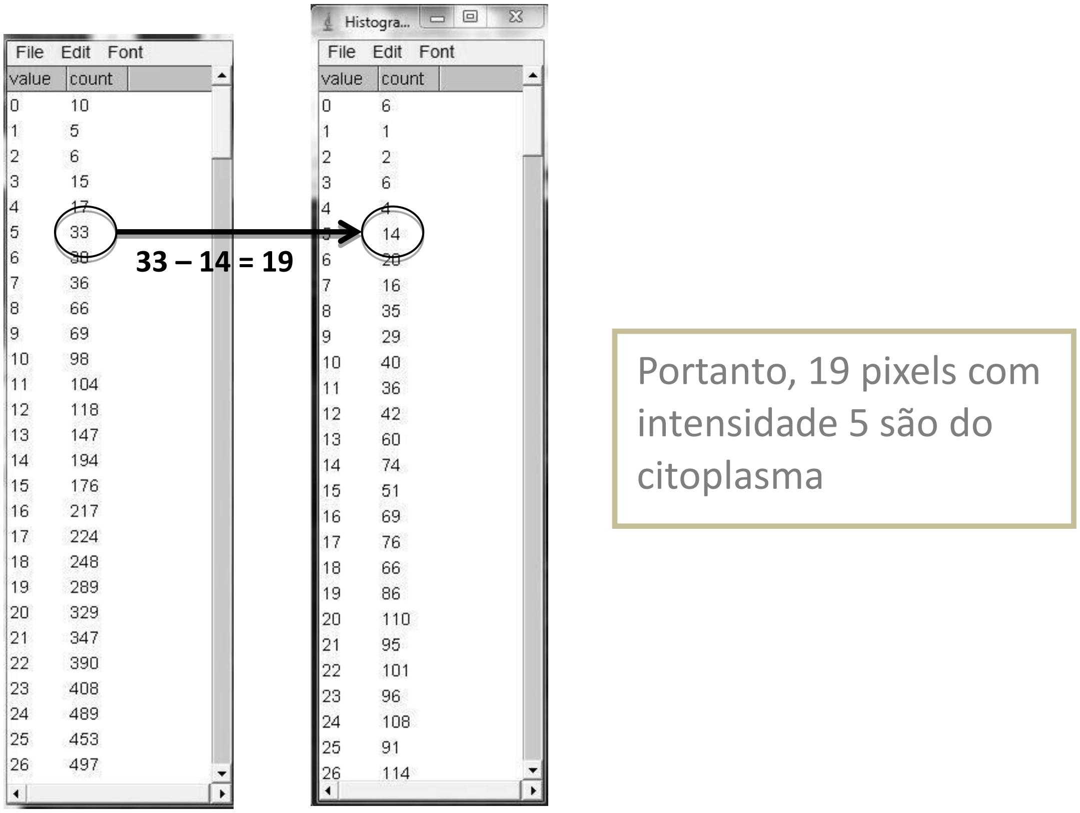

Figura 19 - Cálculo dos pixels dentro da área selecionada para a separação da intensidade de luminescência entre o núcleo e o citoplasma. A coluna de "*value*" representa o valor do pixel e a coluna "*count*" indica quantos pixes há com determinado valor.

3.9.2 Coleta das imagens da parede pélvica renal

Após a imunofluorescência com o anticorpo de interesse SP e CGRP, no microscópio confocal foram coletadas 5 campos ao longo da parede pélvica renal como exemplificado na figura 20. Após as coletas das imagens digitalizadas, estas foram analisadas no software Image J® (figura 21)

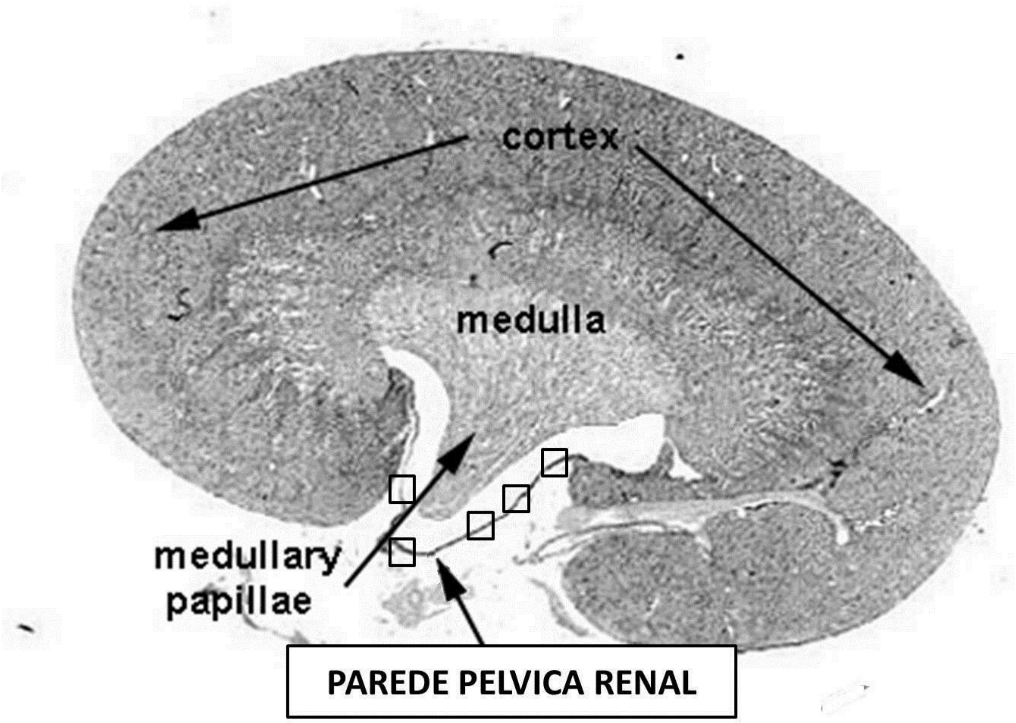

Figura 20 - Análise das imagens digitalizadas por microscopia confocal através do software Image J. Coleta de 5 imagens aleatórias da parede pélvica renal.

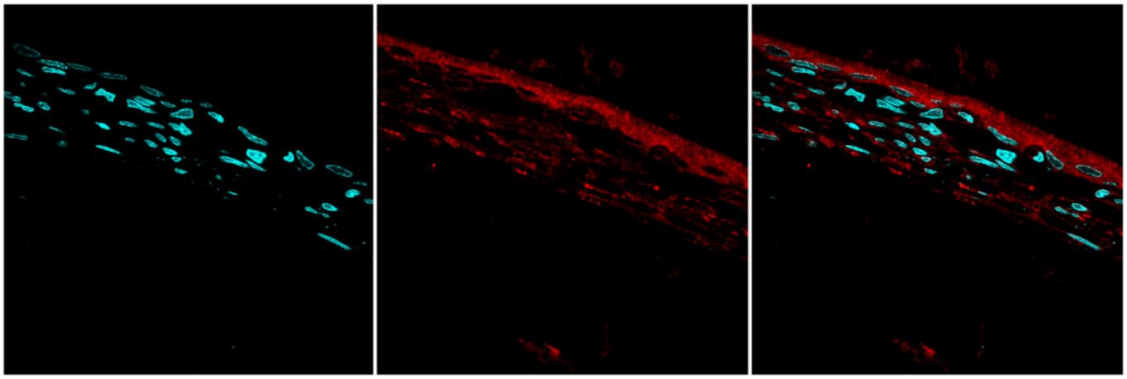

Figura 21- Análise das imagens digitalizadas por microscopia confocal através do software Image J. Campo da Pelve renal de rato com 16 semanas, média da intensidade dos pixels dentro da escala (0-255).

3.10 Analise estereológica dos Gânglios da Raiz Dorsal

Em Criostato, o GRD da vértebra T13 direita foi completamente seccionado sendo confeccionadas lâminas com cortes histológicos de 7µm de espessura com intervalos de 14 µm entre os cortes. Amostras de ambos os grupos foram coletadas NP, n=3 e LP, n=3. Posteriormente os cortes foram hidratados, corados com DAPI e a contagem dos núcleos dos neurônios foi feita em microscópio de fluorescência no aumento de 20X (Figura 22). Após a contagem total de neurônios, as amostras foram normalizadas pelos pesos dos respectivos animais. Os dados foram analisados no Software estatístico GraphPad Prism 5® (trial).

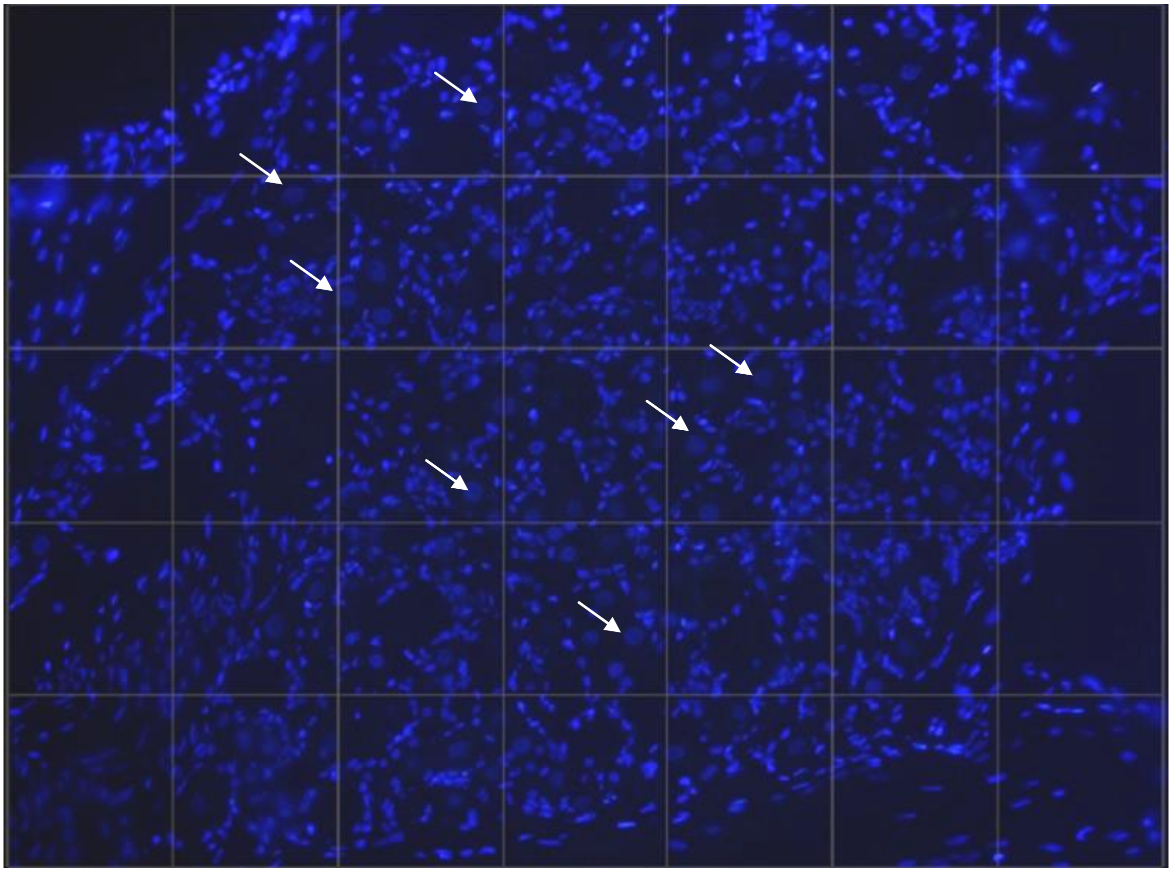

Figura 22 - Corte histológico do GRD T13 direita corado com DAPI, imagem retirada de microscópio de fluorescência no aumento de 20X. Setas indicam exemplos de núcleo de neurônios.

3.11 Análise estatística dos resultados

A análise estatística empregada foi o Test t-student para comparação de uma variável entre 2 grupos. Para as análises da pressão arterial ao longo das semanas de vida, foi utilizada a Análise de Variância (*two-way* ANOVA) com Post-hoc de Bonferroni, já para a comparação da função renal entre 4 grupos foi utilizada Análise de Variância (*one-way* ANOVA) com Post-hoc de Bonferroni. Os resultados foram expressos como media ± Desvio Padrão da Media (DPM). Em todos os cálculos foi fixado um nível critico de 5% ($p<0,05$). O software utilizado em todos os testes estatísticos foi e GraphPad Prism® 5, Copyright 1992-2007 GraphPad Software Inc.

RESULTADOS

4 RESULTADOS

4.1- Controle da massa corporal das mães durante a gestação.

Após a confirmação da prenhez foi realizado o controle da massa corporal das mães, a cada sete dias, durante o período gestacional. Os valores das médias e dos desvios obtidos em fêmeas submetidas à dieta normoproteica (NP, n=10) e hipoproteica (LP, n=10) estão apresentados na figura 23. Os resultados demonstraram que o consumo de dieta hipoproteica durante a gestação, não modificou a massa corporal das mães.

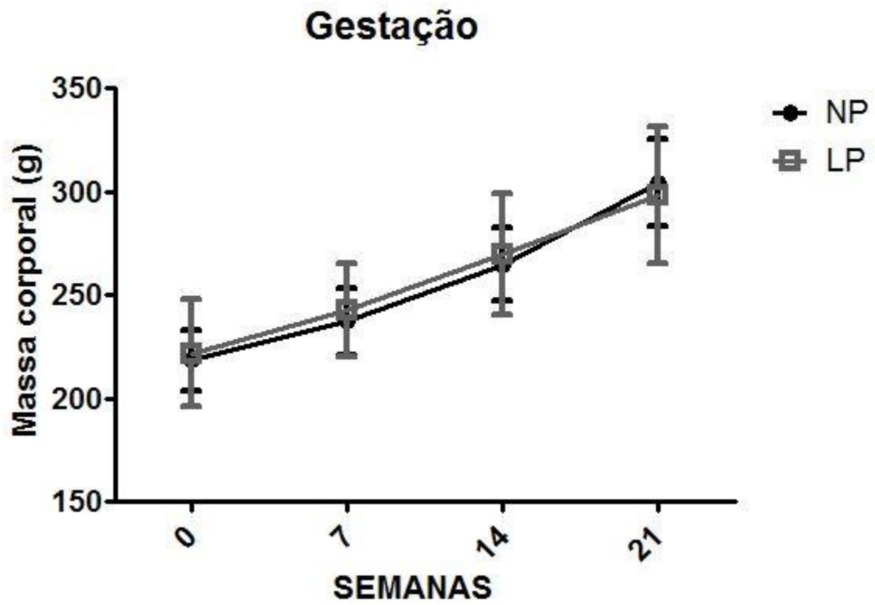

Figura 23 - Ganho de massa (g) ao longo de três semanas de gestação nas ratas b submetidas à dieta NP e LP (NP, n=10 e LP, n=10). Os valores representam média ± DPM.

4.2- Controle da massa corporal da prole de machos nascidos de mães submetidas a dieta NP e LP durante a gestação.

A massa corporal da prole de machos foi aferida no 1° dia pós-natal e aos 21 dias correspondentes ao dia do desmame. A prole de machos derivados de ratas que receberam dieta LP durante a gestação apresentou expressiva redução de massa corporal ao nascer (6.059g ± 0.07524g) quando comparados ao grupo NP (7.442g ± 0.1039g) como podemos observar na figura 24. Foi observado também que a diferença entre os grupos permaneceu até o desmame aos 21 dias de vida (NP: 72.10g ± 1.424 vs LP: 67.91 ± 0.9919) (figura 25).

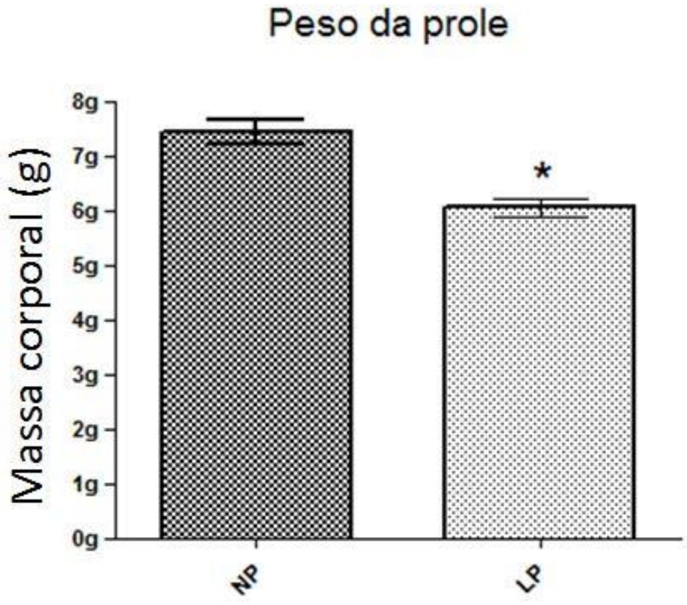

Figura 24 - Massa corporal dos machos da prole de ratas no primeiro dia pós-natal (NP, n=21; LP, n=26). Os valores representam média ± DPM. $^{*}p < 0,0001$.

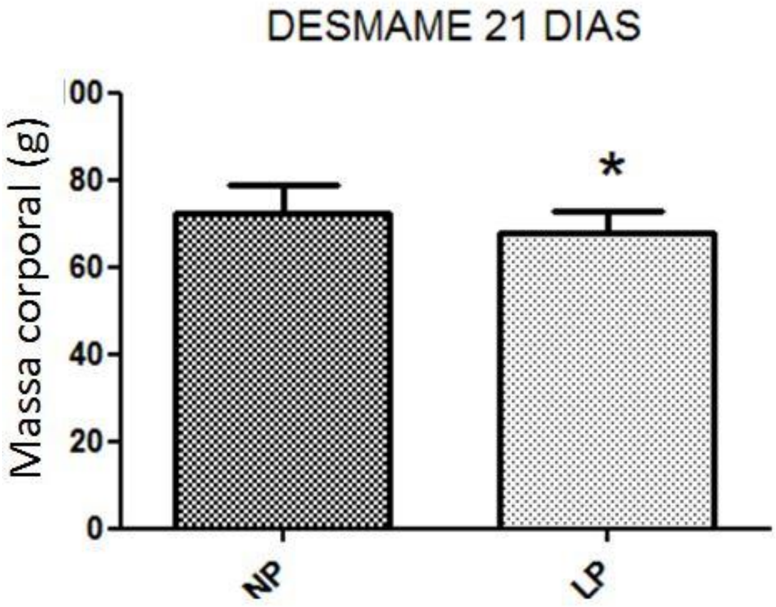

Figura 25 -Massa corporal dos machos da prole de ratas no 21º dia pós-natal (NP, n=20; LP, n=17). Os valores representam média ± DPM. $^{*}p < 0,05$.

4.3- Resultados da aferição da pressão arterial caudal (P.A.C.)

Experimento 1.

Os resultados obtidos pela aferição da pressão arterial sistólica demonstraram, no 1° experimento, que as médias das pressões dos animais do grupo LP foram maiores que as obtidas em animais controle, a partir da 8ª semana de vida até a 16ª, porém, foram observadas diferenças significativas somente na 8ª (NP: 126±5,8 vs LP: 138±9,3), na 14ª (NP: 131±5,4 vs LP: 141±7,0) e na 16ª (NP: 131±4,2 vs LP=147±8,7) semana de vida, como ilustrado na figura 26.

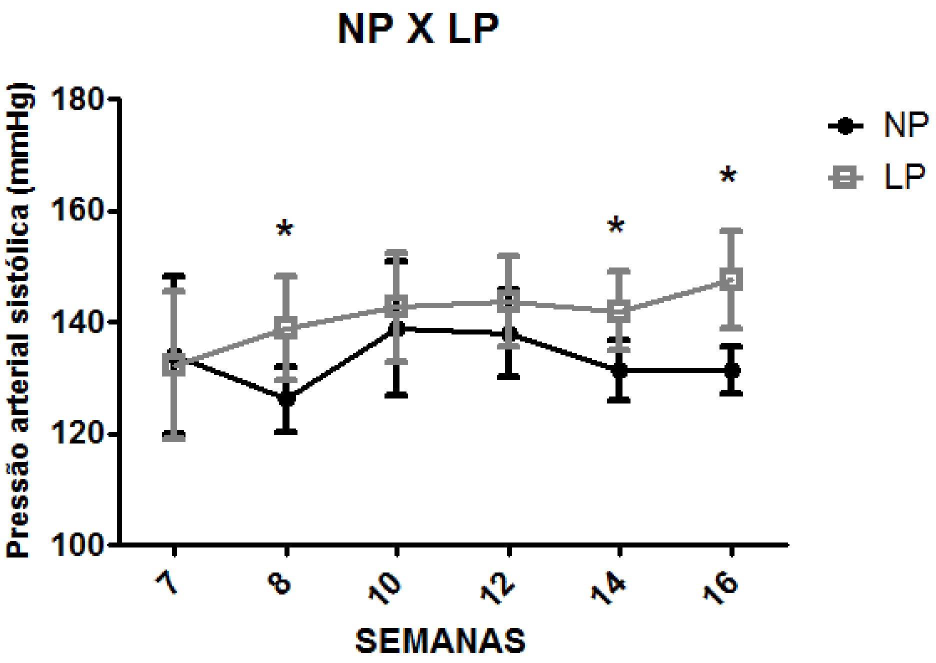

Figura 26 - Efeito da nutrição materna sobre a pressão arterial sistólica da prole ao longo das semanas de vida (NP, n=15; LP, n=15). Os valores representam a média ± DPM. * $p<0{,}05$ (*two-way* ANOVA).

Experimento 2

As aferições da pressão arterial sistólica dos animais do 2° experimento mostraram que as médias das pressões dos animais do grupo experimental LP-SHAM também permaneceram maiores que as obtidas nos animais do grupo controle (NP-SHAM), sendo estas estatisticamente significativas da 7ª até a 16ª semana de vida. (Figura 27). Após a denervação renal bilateral, na 8ª semana de vida, os animais do grupo LP-DNX apresentaram redução significativa da pressão arterial sistólica, a partir da 10ª semana de vida, quando comparado ao seu controle LP-SHAM (figura 28). Assim, como observado na figura 29, os animais do grupo LP-DNX apresentaram valores pressóricos equiparáveis aos obtidos nos animais do grupo controle (NP-SHAM).

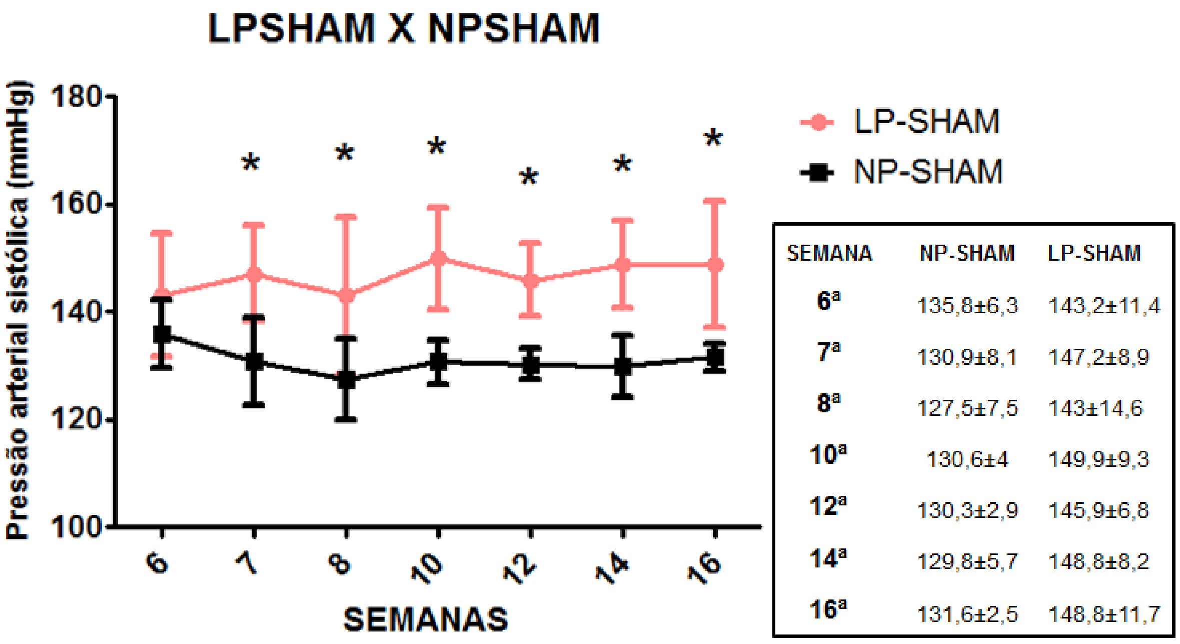

SEMANA	NP-SHAM	LP-SHAM
6ª	135,8±6,3	143,2±11,4
7ª	130,9±8,1	147,2±8,9
8ª	127,5±7,5	143±14,6
10ª	130,6±4	149,9±9,3
12ª	130,3±2,9	145,9±6,8
14ª	129,8±5,7	148,8±8,2
16ª	131,6±2,5	148,8±11,7

Figura 27 - Efeito da nutrição materna e da denervação renal bilateral sobre a pressão arterial sistólica da prole ao longo das semanas de vida (NP-SHAM, n=8; LP-SHAM, n=8). Os valores representam a média ± DPM. * *p*<0,05 (*two-way* ANOVA).

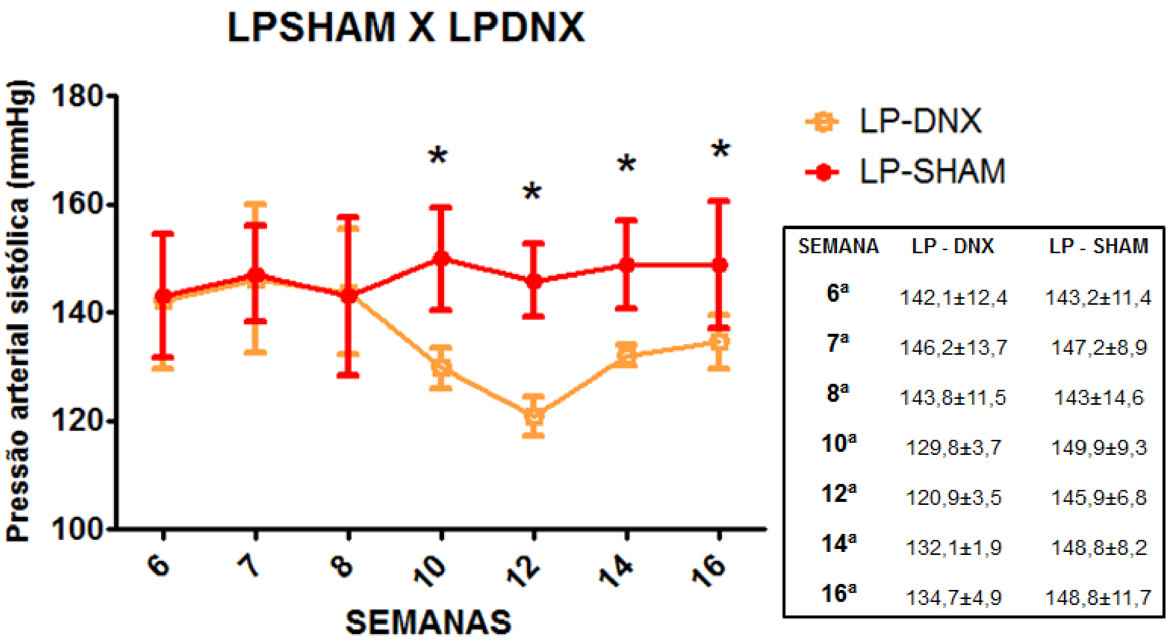

SEMANA	LP - DNX	LP - SHAM
6ª	142,1±12,4	143,2±11,4
7ª	146,2±13,7	147,2±8,9
8ª	143,8±11,5	143±14,6
10ª	129,8±3,7	149,9±9,3
12ª	120,9±3,5	145,9±6,8
14ª	132,1±1,9	148,8±8,2
16ª	134,7±4,9	148,8±11,7

Figura 28 - Efeito da nutrição materna e da denervação renal bilateral sobre a pressão arterial sistólica da prole ao longo das semanas de vida (LP-SHAM, n=8; LP-DNX, n=8). Os valores representam a média ± DPM. * *p*<0,05 (*two-way* ANOVA).

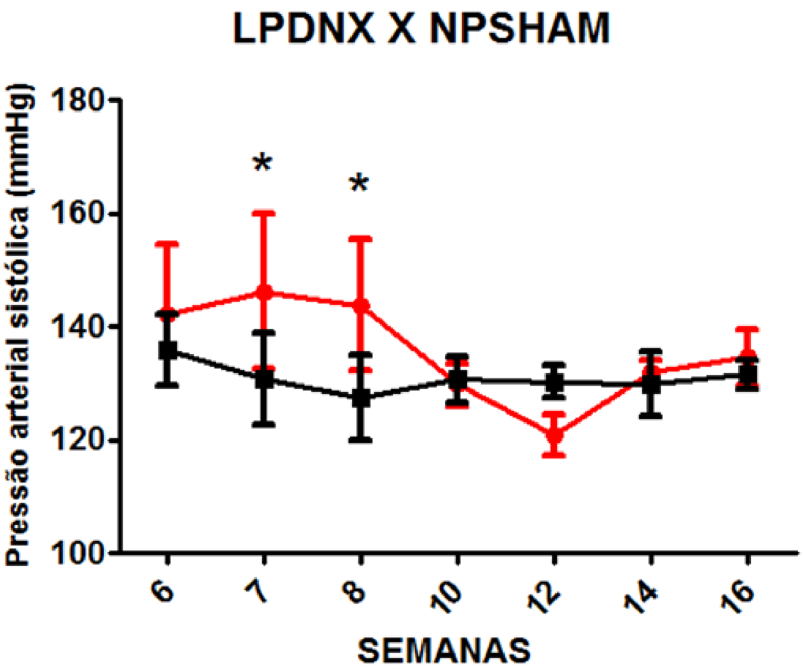

SEMANA	LP - DNX	NP - SHAM
6ª	142,1±12,4	135,8±6,3
7ª	146,2±13,7	130,9±8,1
8ª	143,8±11,5	127,5±7,5
10ª	129,8±3,7	130,6±4
12ª	120,9±3,5	130,3±2,9
14ª	132,1±1,9	129,8±5,7
16ª	134,7±4,9	131,6±2,5

Figura 29- Efeito da nutrição materna sobre a pressão arterial sistólica da prole ao longo das semanas de vida (NP-SHAM, n=8; LP-DNX, n=8). Os valores representam a média ± DPM. * *p*<0,05 (*two-way* ANOVA).

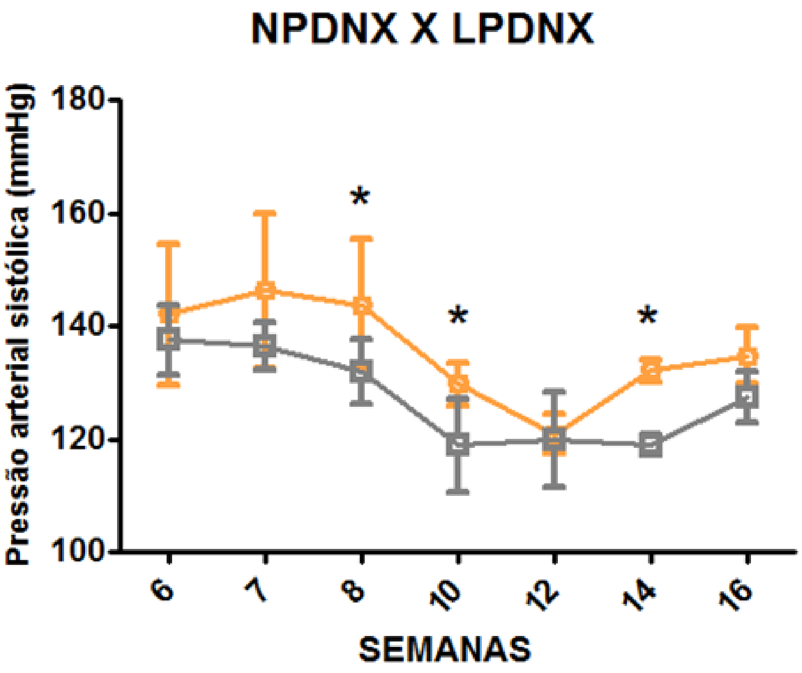

SEMANA	NP - DNX	LP - DNX
6ª	137,5±6,1	142,1±12,4
7ª	136,5±4,3	146,2±13,7
8ª	131,9±5,7	143,8±11,5
10ª	118,9±8,4	129,8±3,7
12ª	119,8±8,3	120,9±3,5
14ª	119,1±1,8	132,1±1,9
16ª	127,4±4,4	134,7±4,9

Figura 30 - Efeito da nutrição materna e da denervação renal bilateral sobre a pressão arterial sistólica da prole ao longo das semanas de vida (NP-DNX, n=8; LP-DNX, n=8). Os valores representam a média ± DPM. * *p*<0,05 (*two-way* ANOVA).

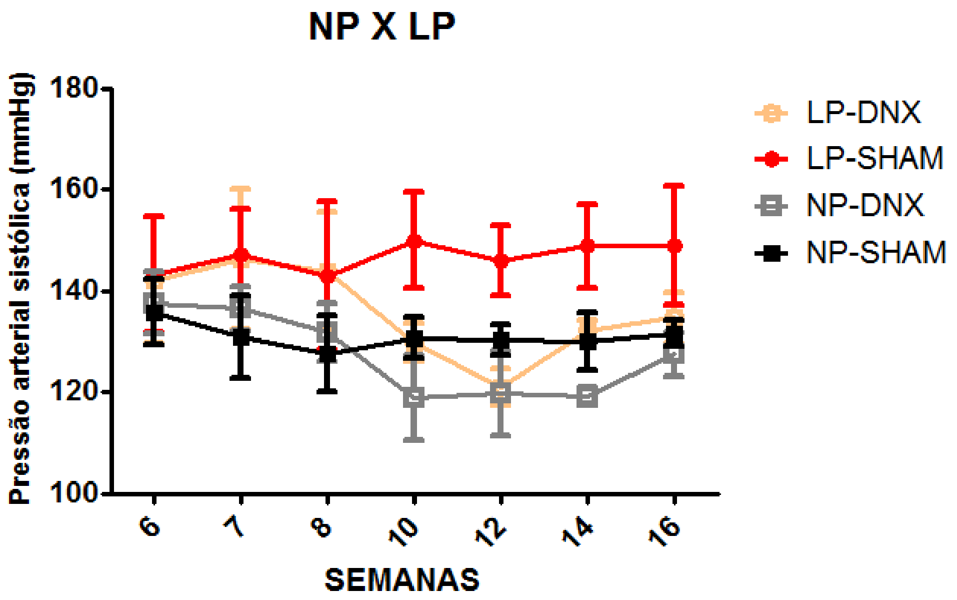

Figura 31 - Efeito da nutrição materna e da denervação renal bilateral sobre a pressão arterial sistólica da prole ao longo das semanas de vida (NP-SHAM, n=8; LP-SHAM, n=8; NP-DNX, n=8; LP-DNX, n=8). Os valores representam a média ± DPM (*two-way* ANOVA).

4.4- Estudo da função renal

Experimento 1.

Os resultados relativos ao estudo da função renal dos ratos do experimento 1, na 10ª e 16ª semana de vida, foram apresentados como média ± DPM. Não observamos alterações na função renal da prole de machos de mães submetidas à restrição proteica gestacional em relação aos controles na 10ª semana de vida (figura 32). Na análise da função renal na 16ª semana de vida não foram observadas alterações no volume urinário (VU) e no clearance de creatinina (CCr). Entretanto, quanto às leituras de sódio, houve redução da fração de excreção total de sódio (FE Na); (NP: 0.135 ± 0.015 vs LP: 0.066 ± 0.002) que ocorreu as custas da redução da fração de excreção pós-proximal de sódio (FEPP Na); (NP: 0.55 ± 0.049 vs LP: 0.26 ± 0.022). Quanto a fração de excreção de potássio (FE K%) não houve diferença entre os grupos (figura 33).

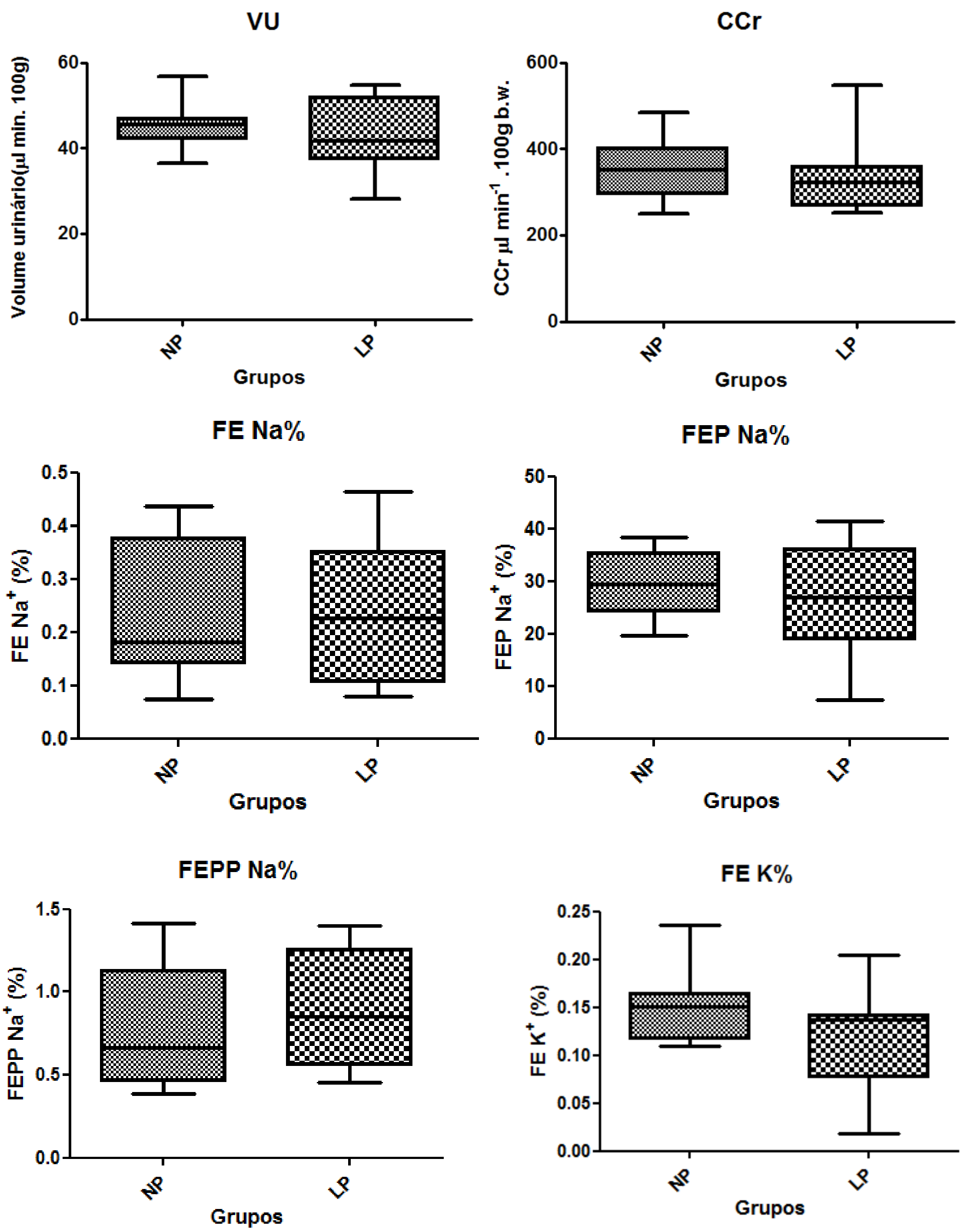

Figura 32 - Função renal na 10ª semana de vida,executada no experimento 1: Clearance de creatinina (CCr) . Fração de excreção total de sódio (FE Na%). Fração de excreção proximal de sódio (FEP Na%). Fração de excreção pós-proximal de sódio (FEPP Na%). Fração de excreção de potássio (FE K%). Os valores representam média ± DPM. * p<0,05 (*teste T student*, NP, n= 7; LP, n= 8).

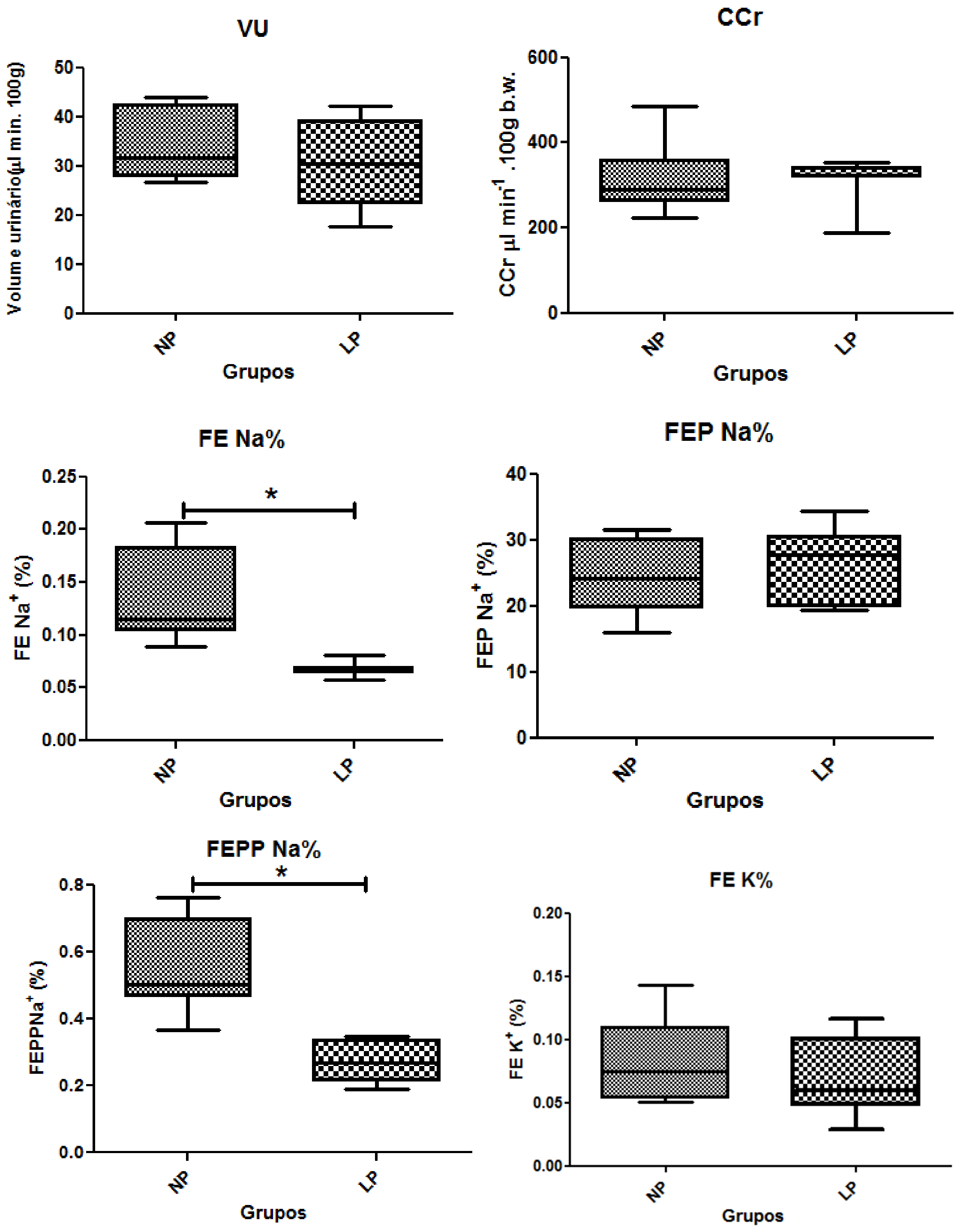

Figura 33 - Função renal na 16ª semana de vida, executada no experimento 1: Clearance de creatinina (CCr) . Fração de excreção total de sódio (FE Na%). Fração de excreção proximal de sódio (FEP Na%). Fração de excreção pós-proximal de sódio (FEPP Na%). Fração de excreção de potássio (FE K%). Os valores representam a média ± DPM. * *p*<0,05 (*teste T student*, NP, n= 7; LP, n= 8).

Experimento 2

Os resultados relativos ao estudo da função renal dos ratos do experimento 2, na 10ª semana de vida, não mostraram alterações no volume urinário e no clearance de creatinina, porém, houve aumento significativo na FEP Na dos animais do grupo LP-DNX, quando comparados aos seu grupo controle LP-SHAM (LP-DNX: 28.15± 3.3 vs LP-SHAM:19.11± 1.27). Este foi acompanhado pelo aumento da FE Na (LP-DNX: 0.25± 0.02 vs LP-SHAM: 0.15± 0.02). A FEPP Na e a FEK não apresentaram alterações quando comparados os valores nesta semana (figura 34).

A análise dos resultados referentes ao estudo da função renal da prole de ratas submetidas à restrição proteica gestacional, na 16ª semana de vida (figura 35) revelou aumento no VU dos animais do grupo NP-DNX em comparação com os animais dos grupos LP-SHAM e LP-DNX (NP-DNX: 38,48±6,21 vs LP-SHAM: 25,36±5,5 e LP-DNX: 22,99±5,4). O clearance de creatinina não foi alterado, porém, a FE Na foi significativamente reduzida em ambos os grupos tratados, LP-SHAM e LP-DNX, quando comparados ao controle NP-SHAM. (NP-SHAM: 0,09±0,03 vs LP-SHAM: 0,04±0,008 e LP-DNX=0,04±0,01). Também houve redução da FEPP Na nos animais LP-SHAM em relação aos animais NP-SHAM (NP-SHAM: 0,58±0,14 vs LP-SHAM: 0,32±0,08). A FEK apresentou-se significativamente reduzida nos animais do grupo LP tanto SHAM quanto DNX, em relação aos dos grupos NP. (NP-SHAM: 0,09±0,01 e NP-DNX: 0,08±0,02 vs LP-SHAM: 0,04±0,01 e LP-DNX=0,04±0,008).

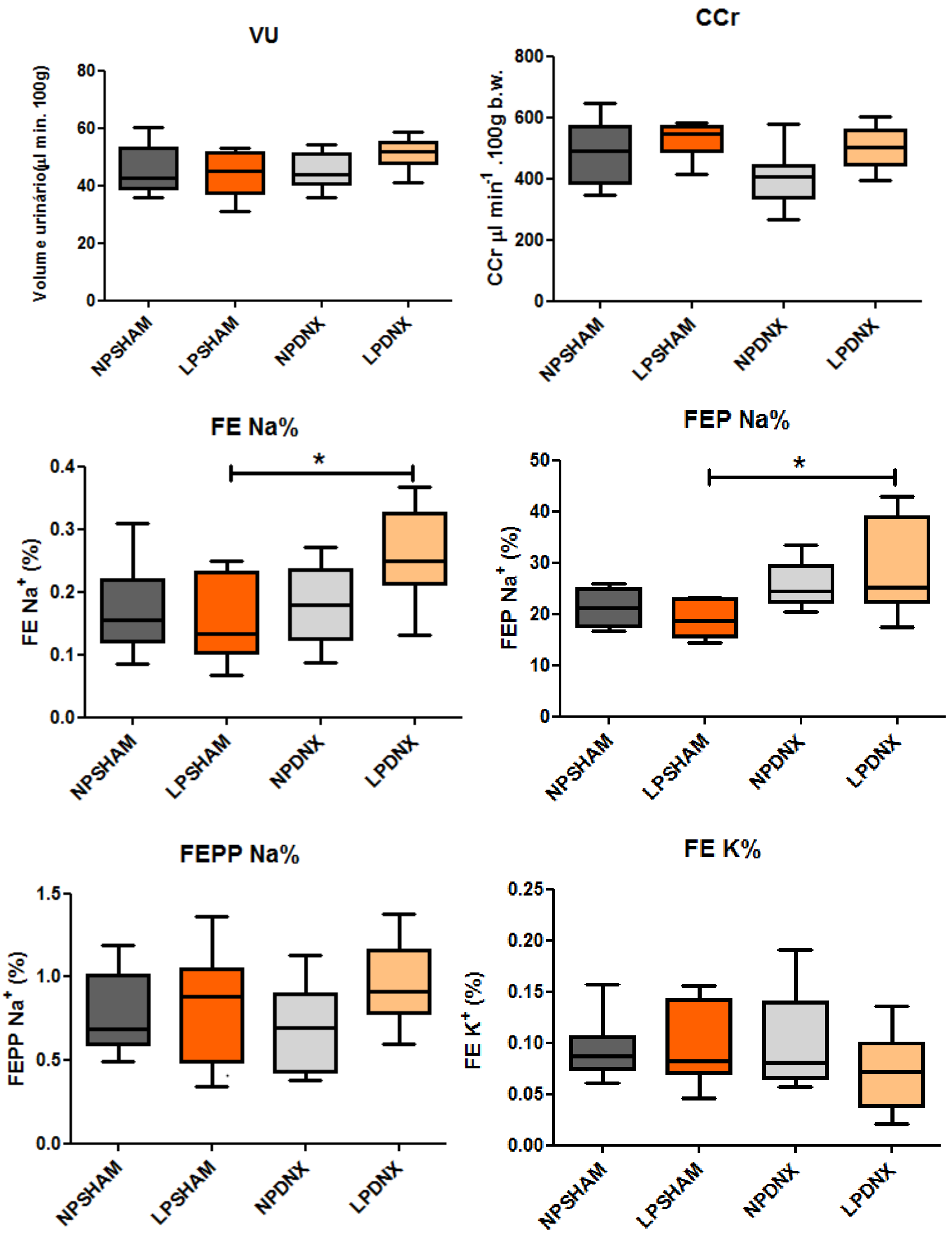

Figura 34 - Função renal na 10ª semana de vida, executada no experimento 2: Clearance de creatinina (CCr) . Fração de excreção total de sódio (FE Na%). Fração de excreção proximal de sódio (FEP Na%). Fração de excreção pós-proximal de sódio (FEPP Na%). Fração de excreção de potássio (FE K%). Os valores representam a média ± DPM. * *p*<0,05 (*one-way ANOVA*, NP-SHAM, n=9; LP-SHAM, n=8; NP-DNX, n=8; LP-DNX, n=8).

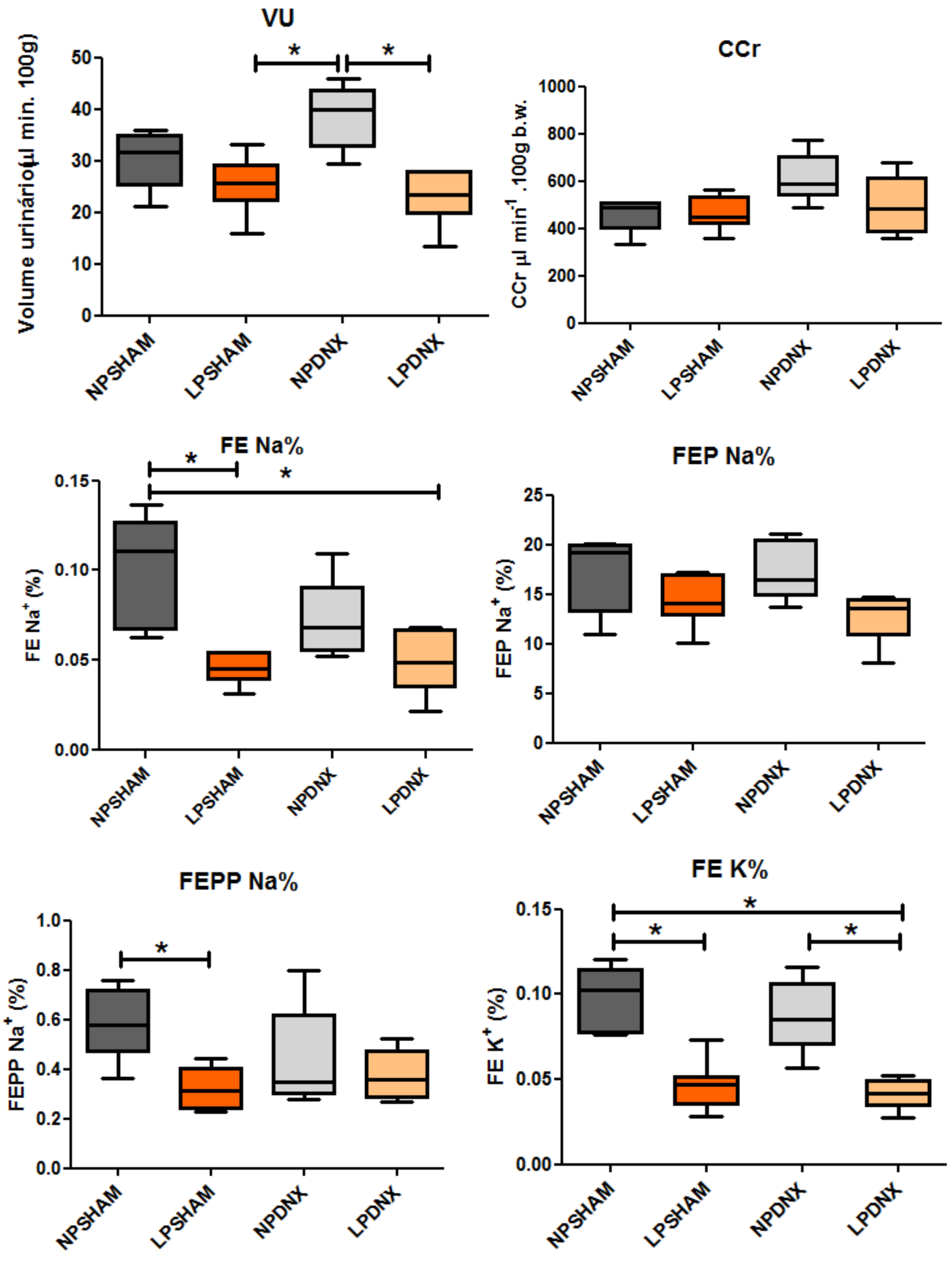

Figura 35 - Função renal na 16ª semana de vida, executada no experimento 2: Clearance de creatinina (CCr) . Fração de excreção total de sódio (FE Na%). Fração de excreção proximal de sódio (FEP Na%). Fração de excreção pós-proximal de sódio (FEPP Na%). Fração de excreção de potássio (FE K%). Os valores representam a média ± DPM. * *p*<0,05 (*one-way ANOVA*, NP-SHAM, n=5; LP-SHAM, n=7; NP-DNX, n=5; LP-DNX, n=6).

4.5- Avaliação morfométrica e estereológica dos neurônios do gânglio da raiz dorsal.

Pela análise das 45 imagens de cada grupo (obtidas por microscopia confocal a Laser e analisadas pelo software Image J®) não observamos diferenças significativas nas áreas ocupadas pelas subpopulações de neurônios do DRG quando comparamos LP e NP como demonstrado na figura 36. Porém, nesta amostragem, observamos redução significativa no número de neurônios pequenos (P) do grupo LP comparativamente ao NP (figura 37).

A análise estereológica dos GRDs, não revelou diferenças significativas no número total de neurônios quando comparamos os resultados obtidos nos grupos NP e LP (Figura 38).

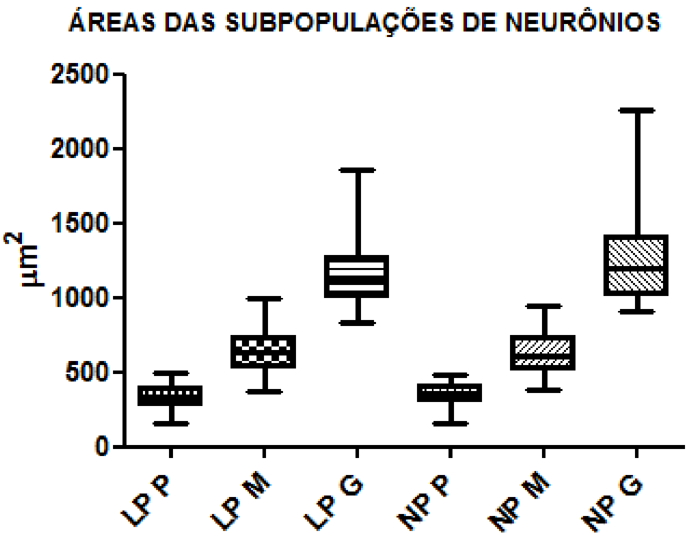

Figura 36 - Efeito da restrição protéica gestacional nas subpopulações de neurônios do gânglio da raiz dorsal da prole. Áreas das diferentes populações de neurônios obtidos de 3 ratos com 16 semanas de vida (NP, n=45; LP, n=45). Os valores representam a média ± DPM. * *p*<0,05 (*teste T student*).

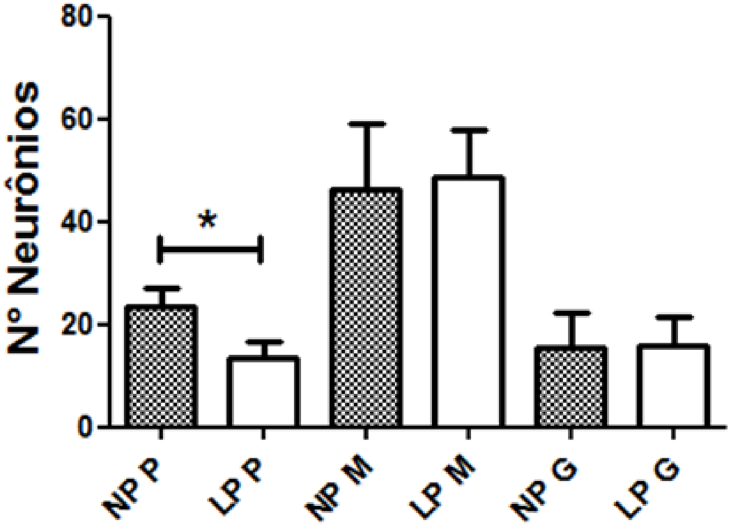

Figura 37 - Efeito da restrição protéica gestacional nas subpopulações de neurônios do gânglio da raiz dorsal da prole. Número de neurônios obtidos de 3 ratos de cada grupo (NP, n=45; LP, n=45). Os valores representam a média ± DPM. * p<0,05 (*teste T student*).

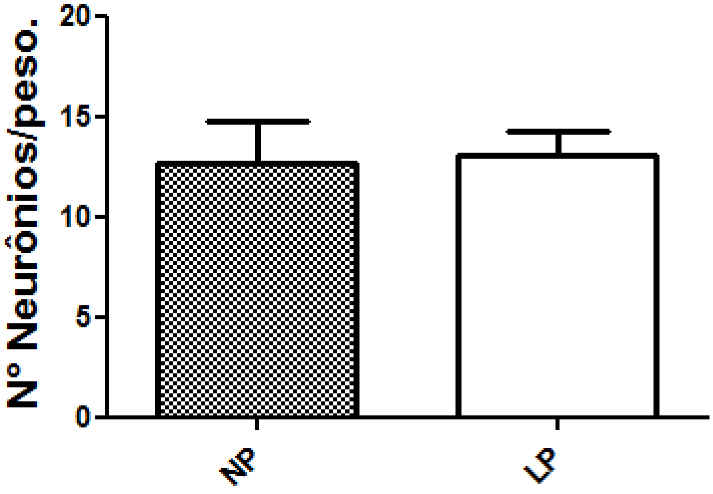

Figura 38 – Análise estereológica do GRD T13 direito. Efeito da desnutrição proteica sobre as populações de neurônios. Número de neurônios totais obtidos de 3 ratos Np vs. LP, dividido pelos seus respectivos pesos. Os valores representam a média ± DPM (*teste T student*).

4.6- Estudo por microscopia confocal a laser de SP, CGRP e NK1R nos neurônios do GRD.

A imunorreatividade de CGRP nos neurônios do gânglio da raiz dorsal (T13) dos ratos do grupo LP apresentou expressiva redução (figura 39) confirmada pela quantificação usando o software Image J® onde, as médias da intensidade de luminescência foram significativamente menores no grupo LP, em todas as subpopulações de neurônios como demonstrado na figura 40. Ao analisarmos separadamente os núcleos (figura 41) e os citoplasmas (figura 42), observamos que em ambos ocorreu redução da imunorreatividade.

As imagens da imunofluorescência para SP também demonstram redução na imunorreatividade nos neurônios dos animais do grupo LP (figura 43) comparativamente àquela obtida em NP. Através do software, foi possível detectar redução na intensidade de fluorescência entre as subpopulações de neurônios pequenos e médios (figura 33). Quando analisamos separadamente os compartimentos nucleares (figura 35) e citoplasmáticos (figura 34) observamos que a redução ocorreu apenas no citoplasma dos neurônios dos animais do grupo LP.

Quanto à imunofluorescência para NK1R observamos aumento sutil nos animais do grupo LP (figura 47). Entretanto, a quantificação pelo software nos neurônios pequenos, médios e grandes revelou aumento significativo de intensidade de lfluorescência em todo neurônio (núcleo + citoplasma) como ilustrado na figura 48. As análises do citoplasma mostraram significativo aumento da imunorreatividade (figura 49), o que também foi observado na leitura do núcleo de todos os neurônios (figura 50).

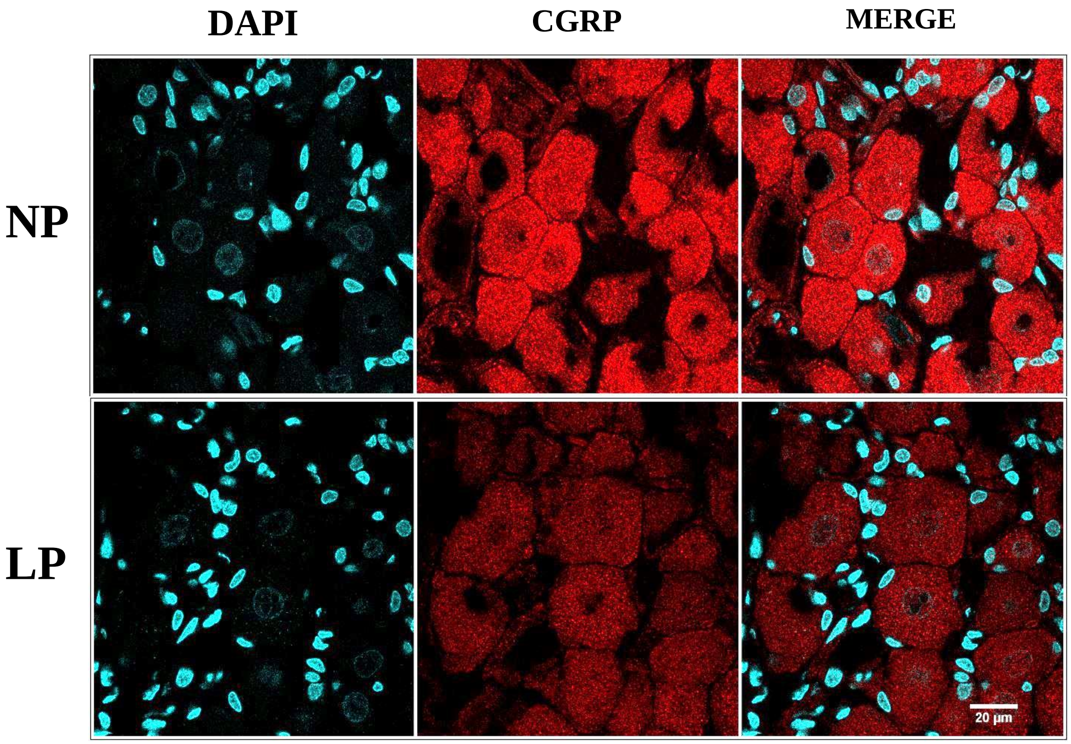

Figura 39- Imunofluorescência para CGRP em GRD T13. Imagens representativas dos resultados obtidos por microscopia confocal a laser. Observe que a redução na imunorreatividade, em todos os neurônios do grupo LP, é patente comparativamente à observada em NP.

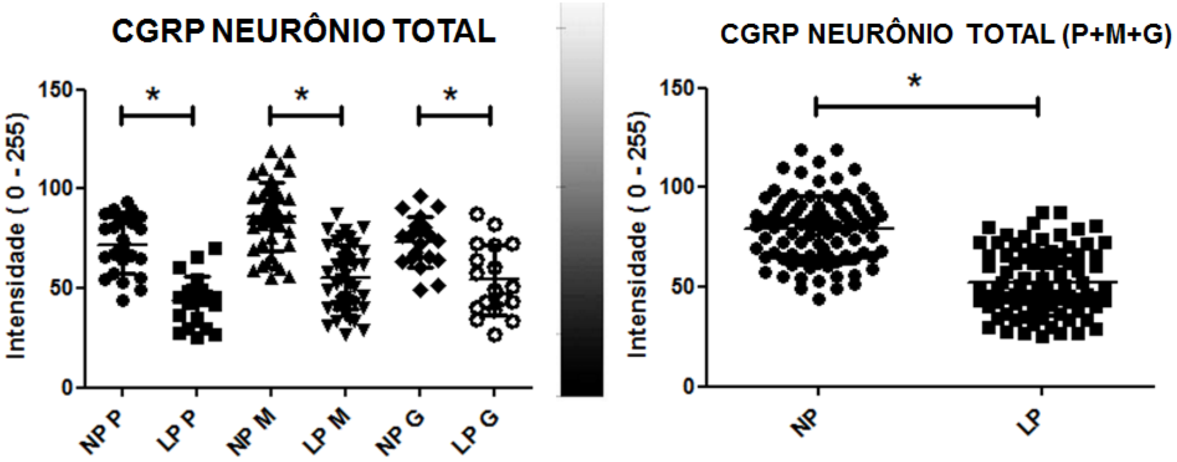

Figura 40 - Quantificação da imunorreatividade de CGRP nas diferentes populações de neurônios e em todos os neurônios (NP, n= 15; LP, n=15). Os valores representam a média ± DPM. * *p*<0,05 (*teste T student*).

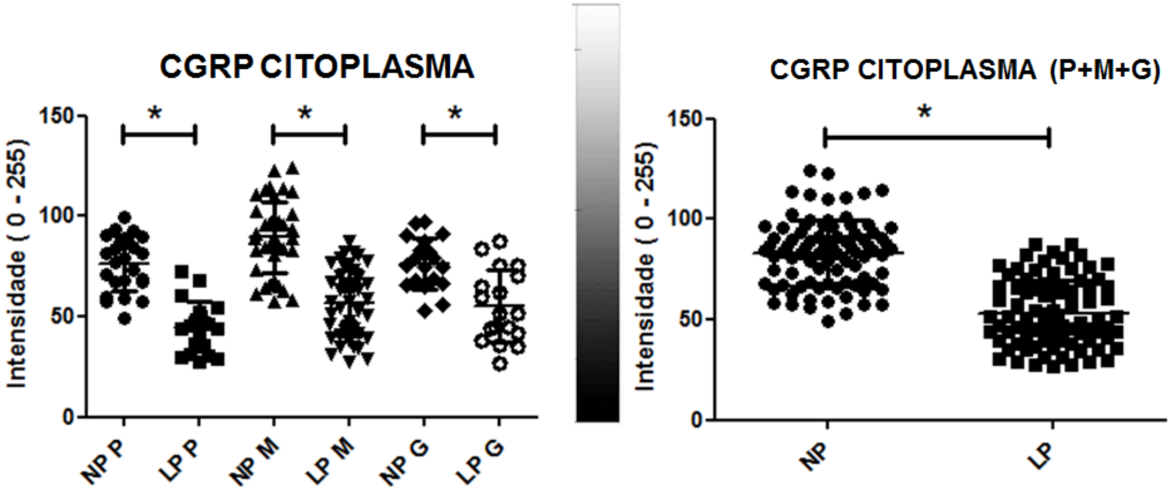

Figura 41 - Quantificação da imunorreatividade de CGRP no citoplasma das diferentes populações de neurônios e em todos os neurônios (NP, n= 15; LP, n=15). Os valores representam a média ± DPM. * *p*<0,05 (*teste T student*).

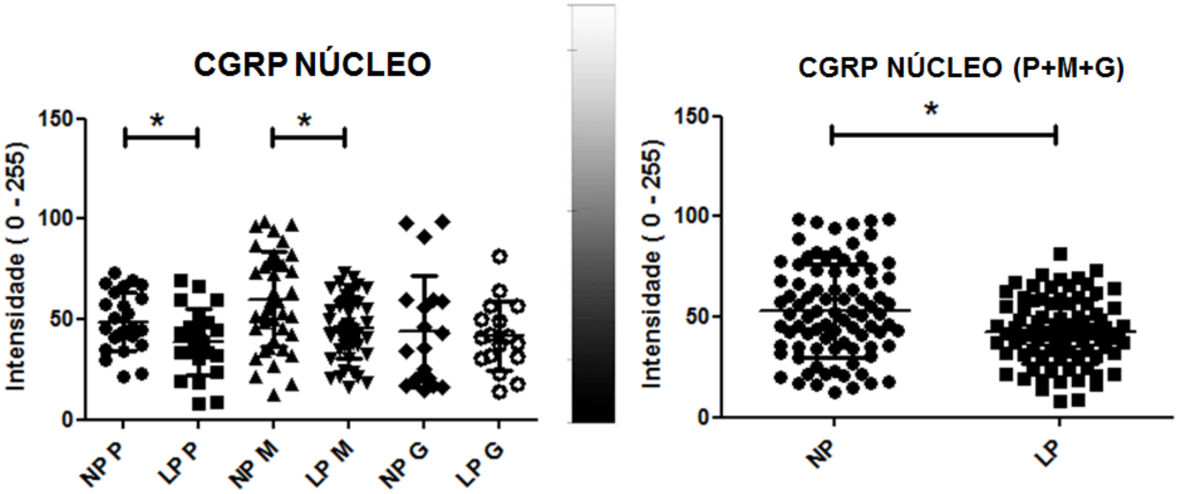

Figura 42 - Quantificação da imunorreatividade de CGRP no núcleo das diferentes populações de neurônios e em todos os neurônios (NP, n= 15; LP, n=15). Os valores representam a média ± DPM. * *p*<0,05 (*teste T student*).

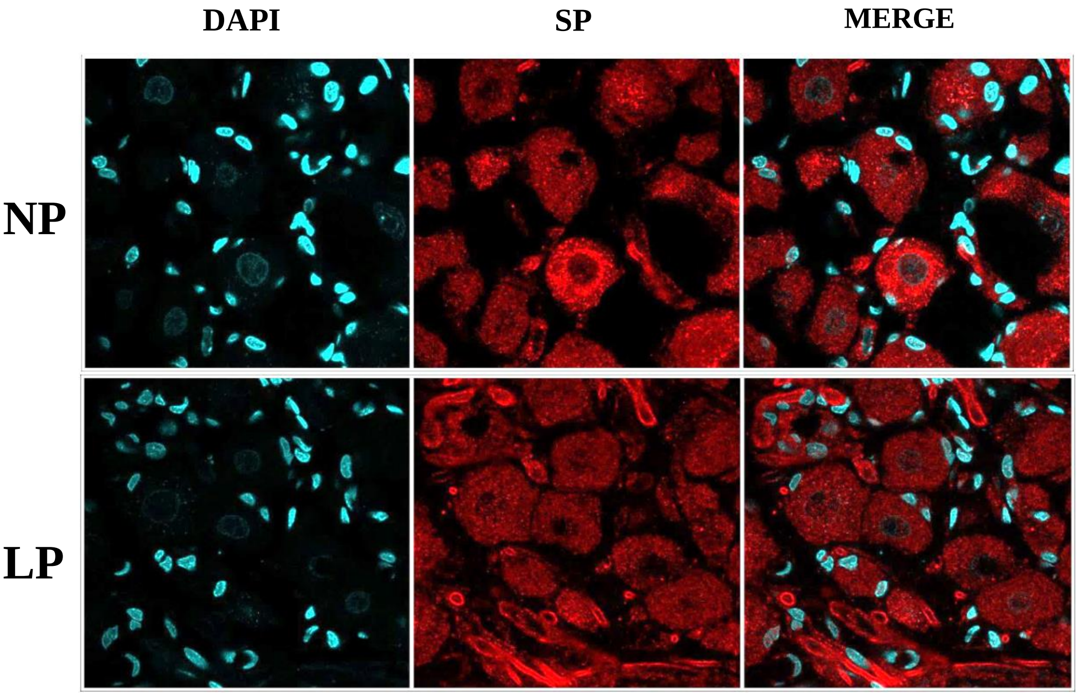

Figura 43 – Imunofluorescência para SP em GRD T13. Imagens representativas dos resultados obtidos por microscopia confocal a laser. Repare a imunorreatividade é menor no grupo LP comparativamente à observada em NP.

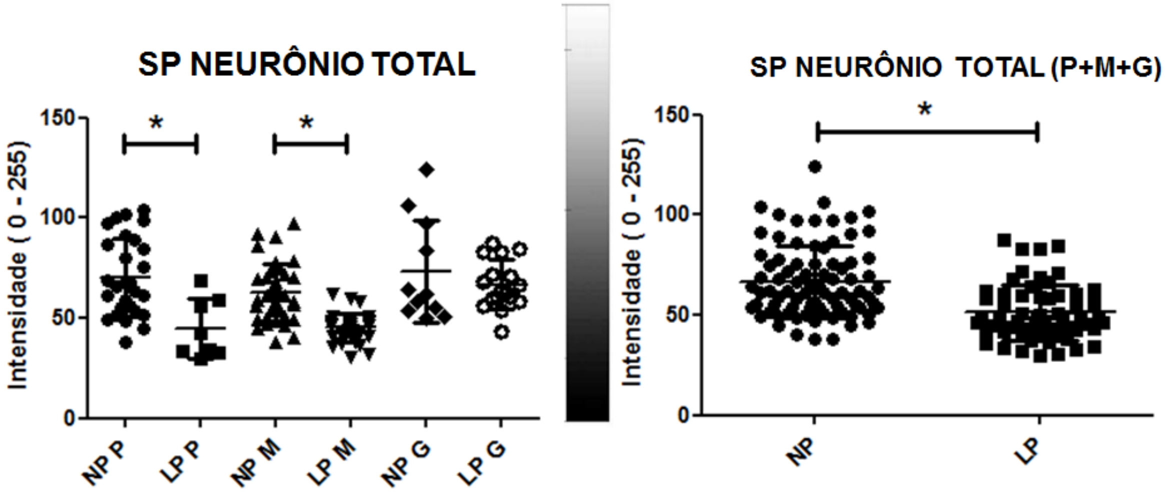

Figura 44 - Quantificação da imunorreatividade de SP nas diferentes populações de neurônios e em todos os neurônios (NP, n= 15; LP, n=15). Os valores representam a média ± DPM. * p<0,05 (*teste T student*).

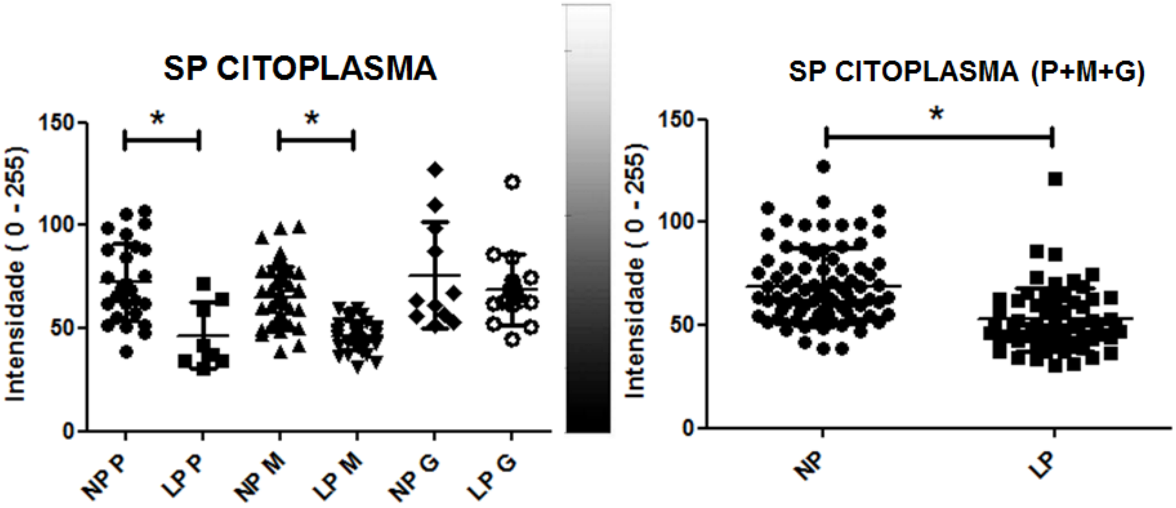

Figura 45 - Quantificação da imunorreatividade de CGRP no citoplasma das diferentes populações de neurônios e em todos os neurônios (NP, n= 15; LP, n=15). Os valores representam a média ± DPM. * *p*<0,05 (*teste T student*).

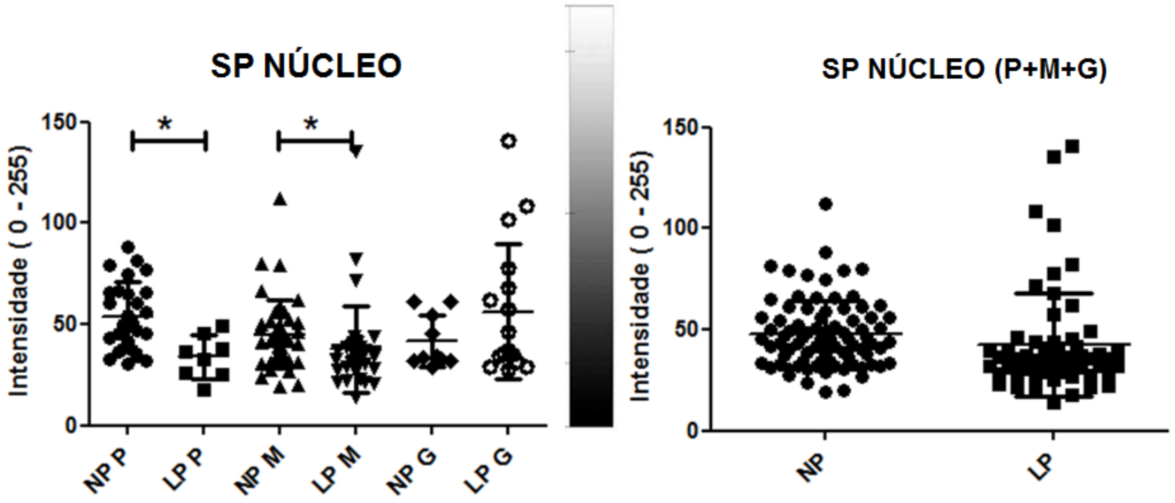

Figura 46 - Quantificação da imunorreatividade de CGRP no núcleo das diferentes populações de neurônios e em todos os neurônios (NP, n= 15; LP, n=15). Os valores representam a média ± DPM. * *p*<0,05 (*teste T student*).

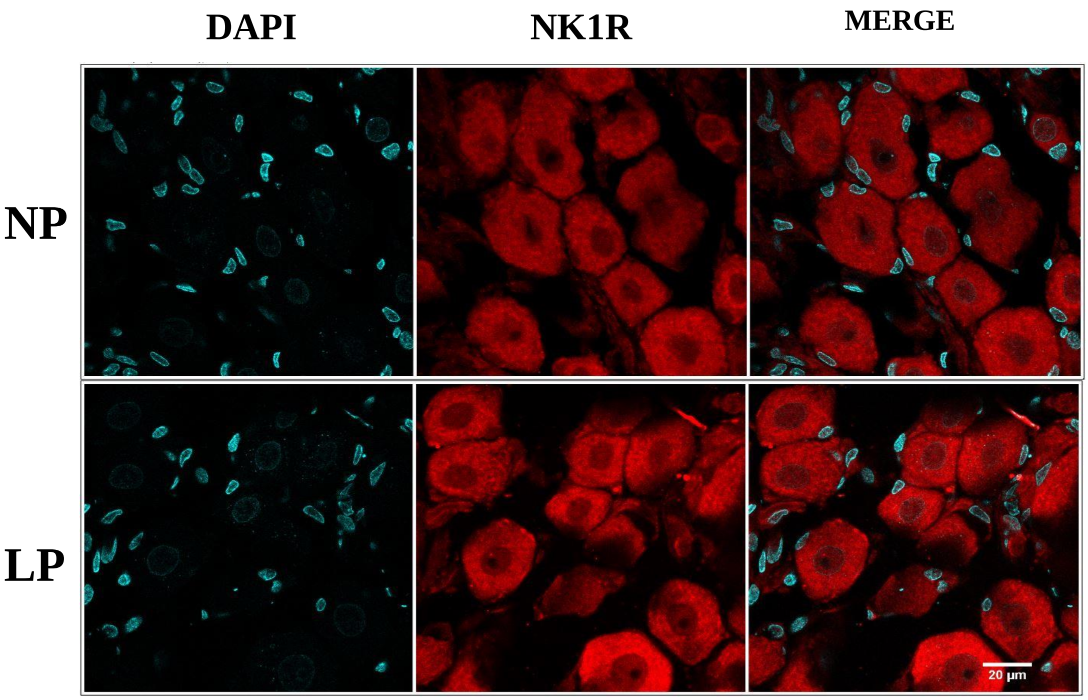

Figura 47 - Imunofluorescência para NK1R em GRD T13. Imagens representativas dos resultados obtidos por microscopia confocal a laser. Repare a imunorreatividade é maior no grupo LP comparativamente à observada em NP.

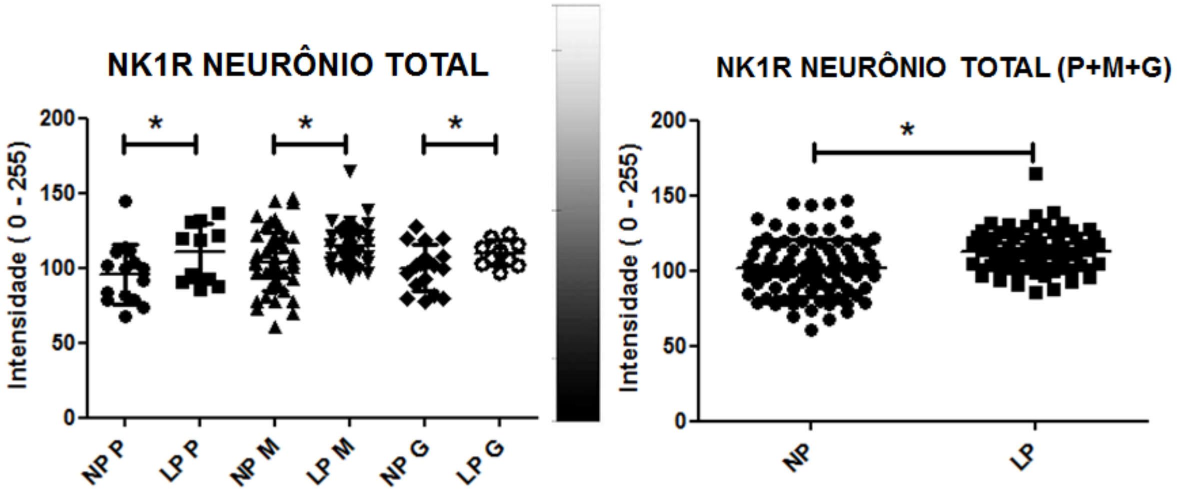

Figura 48 - Quantificação da imunorreatividade de NK1R nas diferentes populações de neurônios e em todos os neurônios (NP, n= 15; LP, n=15). Os valores representam a média ± DPM. * *p*<0,05 (*teste T student*).

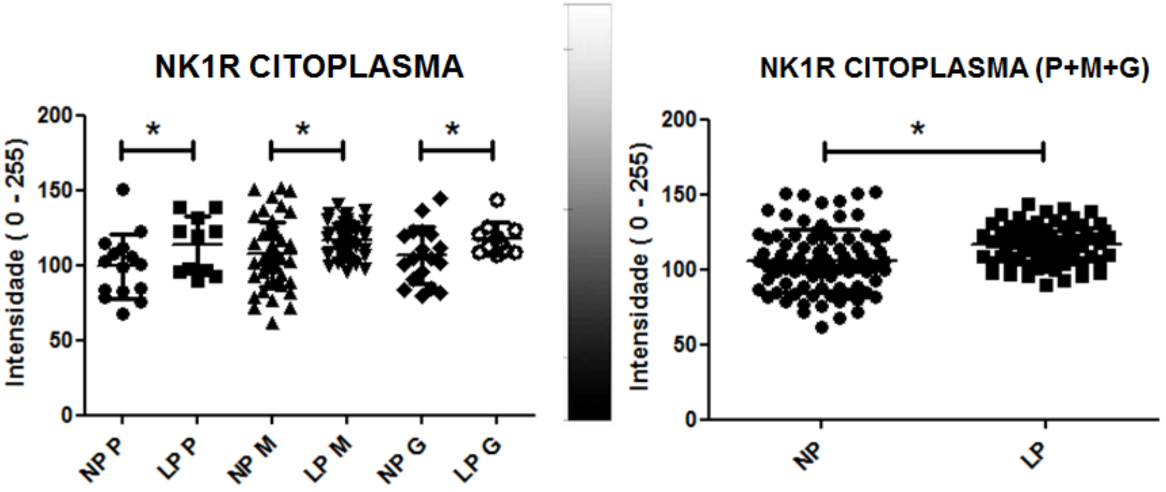

Figura 49 - Quantificação da imunorreatividade de NK1R no citoplasma das diferentes populações de neurônios e em todos os neurônios (NP, n= 15; LP, n=15). Os valores representam a média ± DPM. * *p*<0,05 (*teste T student*).

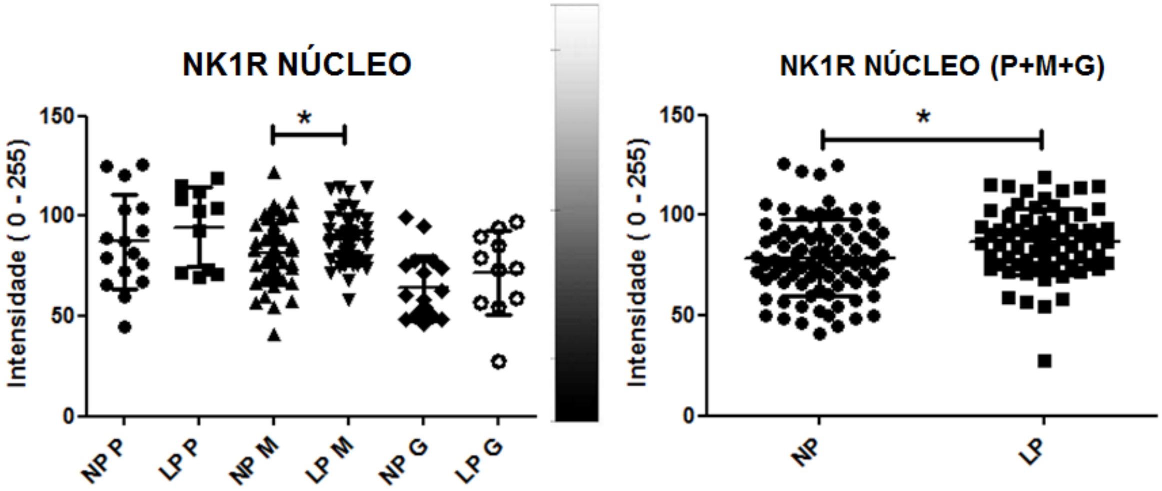

Figura 50 - Quantificação da imunorreatividade de NK1R no núcleo das diferentes populações de neurônios e em todos os neurônios (NP, n= 15; LP, n=15). Os valores representam a média ± DPM. * *p*<0,05 (*teste T student*).

4.7- Estudo por microscopia confocal a laser de SP e CGRP na parede pélvica renal de ratos com 16 semanas de vida.

Ao analisarmos as imagens da imunofluorescência para CGRP observamos que houve aumento na imunorreatividade na parede pélvica renal dos animais do grupo LP (figura 51), confirmado pela quantificação, onde foi possível detectar aumento estatisticamente significativo da intensidade de fluorescência, demonstrado na figura 52.

Quanto à imunofluorescência para SP, nós não observamos alterações na imunorreatividade da parede pélvica renal dos animais do grupo LP, quando comparados ao grupo controle (figura 53). Estas observações foram confirmadas pela quantificação demonstrada na figura 54.

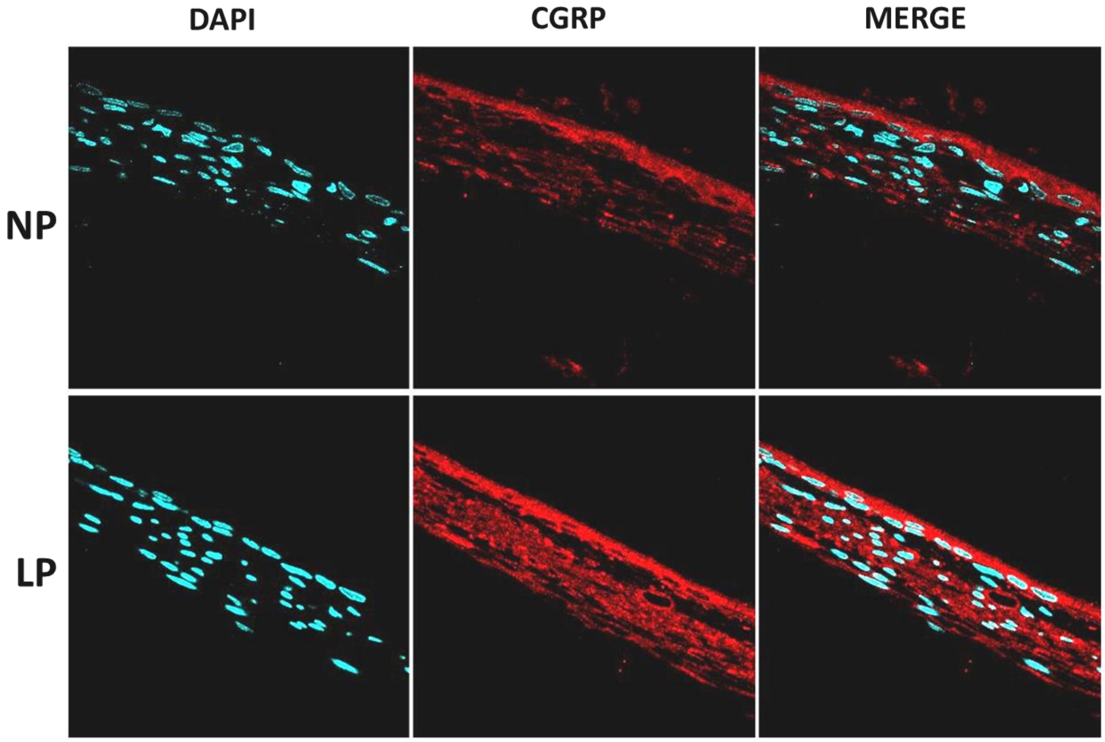

Figura 51 – Imagens representativas da imunofluorescência para CGRP na pelve renal. Repare que a densidade de marcação em LP é maior do que a observada em NP.

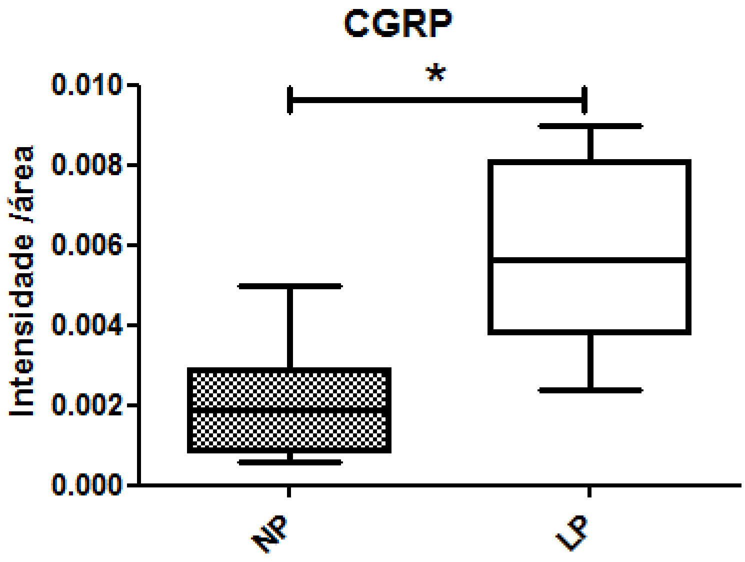

Figura 52 - Quantificação de CGRP na pelve renal de ratos (NP, n= 15; LP, n=15). Os valores representam a média ± DPM. * *p*<0,05 (*teste T student*).

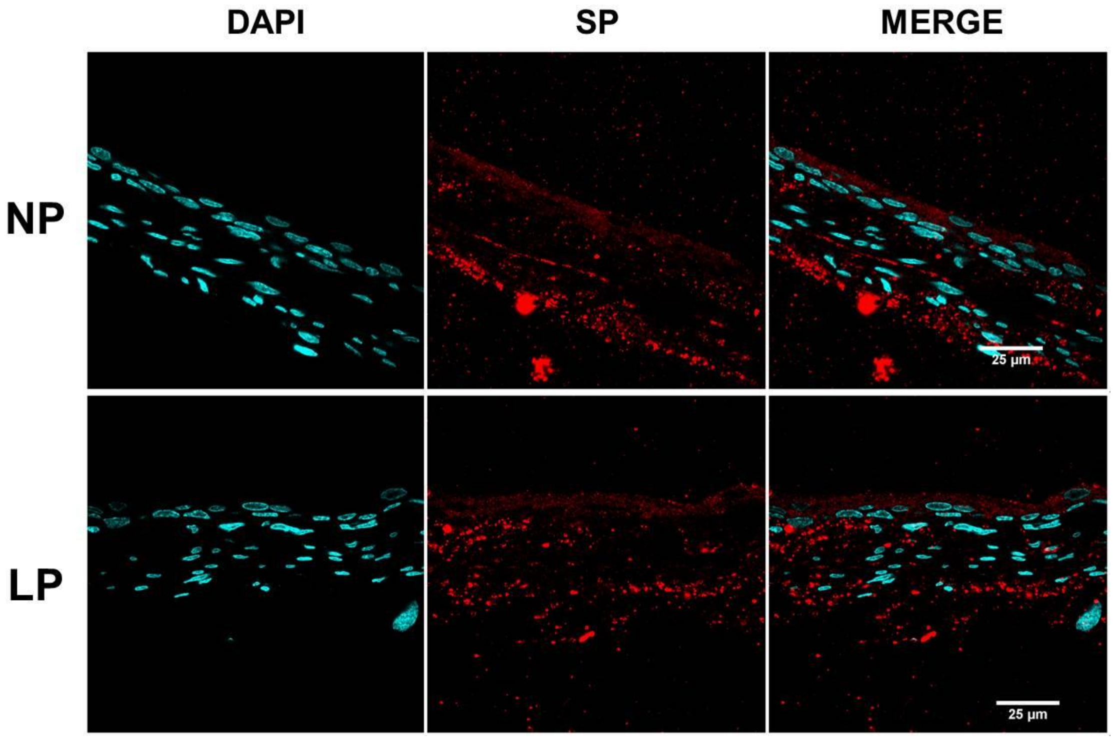

Figura 53 - Imagens representativas da imunofluorescência para SP na pelve renal. Não observamos diferença na marcação em LP comparativamente à observada em NP.

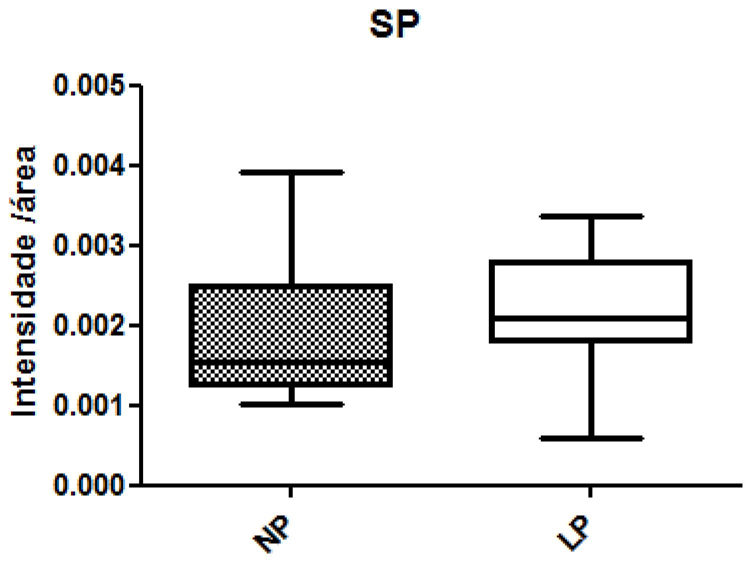

Figura 54 -- Quantificação de SP na pelve renal de ratos (NP, n= 15; LP, n=15). Os valores representam a média ± DPM. * *p*<0,05 (*teste T student*).

DISCUSSÃO

5 DISCUSSÃO

Diversas evidências epidemiológicas e estudos em modelos animais têm demonstrado que, alterações extremas no ambiente intrauterino durante períodos importantes do desenvolvimento podem elevar o risco de doenças cardiovasculares, metabólicas e neuroendócrinas na vida adulta (BARKER et al., 1989;1995; LAW et al., 1991; LANGLEY-EVANS, 1998; ASHTON, 2000; MAO, et al., 2009). A este fenômeno no qual o organismo é moldável durante estes períodos críticos do desenvolvimento, deu-se o nome de programação fetal (HALE e BARKER, 2001; SANDMAN et al.,2011). Em estudos realizados em nosso laboratório, utilizando o modelo de restrição proteica gestacional severa (6% proteína na dieta), foi demonstrado que a prole de ratos machos oriundos destas mães apresenta maior chance de desencadear diversos distúrbios na vida adulta, dentre os quais alterações funcionais renais diretamente relacionadas com o desenvolvimento de hipertensão arterial nestes animais (MESQUITA et. al., 2010).

Assim como outros trabalhos, o presente estudo demonstrou que modificações no ambiente intrauterino causado pela restrição proteica, promove redução na massa corporal da prole ao nascer quando comparados com a prole de mães que receberam a dieta normal com 17% de proteína (LANGLEY-EVANS, 1998; ASHTON, 2000; EDWARDS et. al., 2001; WOODALL et. al., 1996 MESQUITA et. al., 2011; LIMA et. al., 2012,). Este baixo peso ao nascimento é um importante marcador de programação fetal, demonstrando que houve alterações, principalmente no aporte nutricional fetal (LUCAS et. al., 1999). JANSSON et. al. 2006 sugerem que nestes modelos de restrição proteica há uma redução de importantes transportadores placentários de aminoácidos, assim o menor aporte de nutrientes acabam contribuindo para redução do peso ao nascer. Sabe-se também que a exposição fetal a altos níveis de glicocorticoides afetam *in uteri,* o crescimento embriofetal em roedores (BENEDIKTISSON et. al., 1993). A exposição fetal aguda ou crônica a glicocorticoides sintéticos, como a dexametasona, causa além de apoptose de células progenitoras, alterações no desenvolvimento e maturação de diversas células e tecidos. O mecanismo pelo qual ocorre tais alterações estão relacionadas à redução placentária da enzima 11β-hidroxiesteróide

desidrogenase do tipo 2 (11β-HSD- 2), que tem a função biológica de converter nos humanos o cortisol materno em sua forma inativa, a cortisona, e nos animais converte a corticosterona em dehidrocorticosterona. (SECKL et. al., 2007; LANGLEY-EVANS et. al., 1996). Assim, animais filhos de mães submetidas à restrição proteica gestacional estão expostos a elevados níveis de glicocorticoides maternos que contribuem para a reduzida massa corporal dos animais ao nascer, como também observado neste estudo.

Confirmando estudos prévios (MESQUITA et. al., 2011; LIMA et. al., 2012; SCABORA et al., 2013), nossos resultados mostraram significativa elevação da pressão arterial sistólica acompanhada pela redução expressiva da excreção de sódio urinário em animais do grupo LP, sem que tenha havido alterações significativas na filtração glomerular estimada pelo clearance de creatinina (CCr). A dieta materna tem papel importante na ontogênese renal e a restrição de proteína durante a gestação promove redução no número de nefros nestes animais. (OJEDA et. al., 2008, MESQUITA et. al., 2011) Muitos modelos animais de programação fetal apresentam relação inversa entre a redução da massa corporal ao nascer e o desenvolvimento de hipertensão arterial sistêmica. Estudos em animais submetidos à restrição de crescimento intrauterino (RCIU) demonstraram que o desenvolvimento dos rins apresenta alta sensibilidade e alterações ontogênicas à variações do ambiente intrauterino, particularmente, durante as fases iniciais da nefrogênese (LANGLEY-EVANS et. al., 1999). Sendo assim, já está bem definido que o reduzido número de nefros contribui, pelo menos parcialmente, para a elevação da pressão arterial na vida adulta na prole de ratas submetidas à restrição proteica gestacional (LANGLEY-EVANS et. al., 1999; MESQUITA et al., 2011). Entretanto os mecanismos pelos quais isto ocorre ainda são mal entendidos (MESQUITA et. al., 2011). Devemos levar em consideração também as possíveis alterações causadas em outros órgãos e sistemas responsáveis pela manutenção da pressão arterial e pelo balanço hidroeletrolítico.

É sabido que animais, nascidos de mães que sofreram restrição proteica durante a gestação apresentam diversas alterações de desenvolvimento do sistema nervoso central, dentre eles reduzido número de neurônios no tronco cerebral nos períodos iniciais da vida, menor quantidade de receptores AT1 e AT2 no núcleo do trato solitário

(NTS). Além de alterações destes mesmos receptores no hipotálamo (SCABORA et al., 2013; DE LIMA et al., 2012). O NTS assim como, a medula rostral ventrolateral e áreas do hipotálamo são importantes regiões do SNC, envolvidas na homeostase hidrossalina e atividade autonômica que implicam no controle central dos reflexos barorreceptores e quimiorreceptores, fazendo a modulação da atividade simpática cardiovascular. Alterações nestas regiões do SNC apresentam um potencial para o desenvolvimento de hipertensão arterial relacionada a distúrbios do metabolismo de água e sal bem como da atividade autonômica (GUYENET, 2006). A atividade simpática renal é um importante modulador da excreção dos eletrólitos e quando alterada, promove maior ou menor retenção de sais, principalmente sódio, podendo contribuir para o aumento da volemia e consequentemente a hipertensão arterial sistêmica. Estudos sobre a atividade nervosa renal neste modelo animal são escassos.

Tendo em conta as observações acima, é provável que animais programados (LP) apresentem maior atividade simpática renal que, assim como animais SHR, levam a menor fração de excreção urinária de sódio o que contribuiria, pelo menos em parte, para a elevação da pressão arterial sistólica na prole de animais LP. Sabe-se que a atividade neural aferente renal tem papel importante na modulação da atividade nervosa eferente renal, sendo esta última a moduladora da manipulação de eletrólitos pelos diferentes segmentos tubulares renais. Até o momento, não se conhecia se em animais programados ocorria elevação da atividade nervosa eferente renal, contribuindo com a maior retenção de sódio e água pelos rins, ou se a atividade aferente está reduzida, deixando de fazer seu papel modulador negativo sobre a atividade eferente simpática renal. Como observado no presente estudo, após denervação renal bilateral na 8ª semana de vida, houve uma expressiva redução da pressão arterial sistólica nos animais do grupo LP-DNX, sendo significativa a partir da 10ª semana de vida, acompanhada por um aumento da excreção de sódio quando comparado ao seu grupo controle LP-SHAM.

O sistema nervoso simpático tem papel fundamental na regulação do débito cardíaco, da pressão sanguínea e do equilíbrio hidroeletrolítico, mantendo a homeostase no fluxo tecidual e sobre a composição dos fluidos corporais. Barorreceptores e quimiorreceptores têm a função de enviar ao sistema nervoso central

sinais sensoriais. A atividade do sistema nervoso simpático é modulada pelo núcleo do trato solitário, localizado no mesencéfalo, que são coordenados por estruturas neurais diencefálicas e corticais. Os sinais eferentes simpáticos regulam a pressão arterial por de um conjunto de modificações hemodinâmicas, mediadas por receptores adrenérgicos, tais como receptores β-adrenérgicos que modificam a contratilidade cardíaca e consequentemente, o débito cardíaco e promovem a secreção de renina pelos rins. Simultaneamente, efeitos sobre a vasoconstrição periférica ocorrem pela ativação de receptores α–adrenérgicos, também responsáveis pela maior retenção de sódio e água nos túbulos renais. (ESLER, 2010)

Evidências clínicas têm demonstrado interação entre a atividade simpática renal e a atividade simpática global, através de achados em pacientes com doença renal crônica, em estágio final, nos quais a elevada atividade do sistema nervoso simpático foi reduzida após a nefrectomia, equiparando-se aos níveis encontrados em indivíduos saudáveis (CONVERSE, et al., 1992). No entanto, a maior parte destas evidências vem de estudos com animais. O papel crucial dos nervos aferente e eferente renais, sobre o controle da pressão arterial tem sido demonstrado em vários modelos animais de hipertensão arterial associado ou não à doenças renais pré-existentes (KRUM et al., 2011). A denervação renal bilateral foi capaz de reduzir os níveis venosos e urinários renais de noradrenalina em cerca de 80%, pós-estimulação simpática em modelos animais, (BRADLEY e HJEMDAHL, 1984). A estimulação dos nervos eferentes renais também promove aumento da resistência vascular renal contralateral associada à elevação da pressão arterial média. (BRADLEY e HJEMDAHL, 1986). Observando a ação tubular renal da atividade simpática renal, os receptores α-adrenérgicos induzem efeitos antinatriurético nos túbulos proximais com expressiva retenção de sódio, associado à vasoconstricção induzida por receptores α-adrenérgicos, reduzindo o fluxo sanguíneo renal. Já os receptores β1-adrenérgicos estimulam a secreção de renina pelas células justaglomerulares com subsequente ativação do sistema renina-angiotensina-aldosterona (WYSS et al., 1999), bem como a reabsorção tubular renal de sódio. No entanto, em diferentes espécies e modelos animais de hipertensão arterial, os efeitos e distribuição no parênquima renal de receptores α-adrenérgicos são variáveis. (HERING, et. al. 2011)

Com a completa denervação renal bilateral, há a supressão das vias aferentes e eferentes renais, reduzindo assim a atividade eferente simpática oriunda do SNC, reduzindo sua reabsorção tubular de sódio com observado no presente estudo a partir da 10ª semana de vida nos animais do grupo LP-DNX. Este efeito da supressão simpática renal ocorre a despeito da redução da atividade neurossensorial renal, que modula negativamente a atividade simpática. Vale ressaltar que, a partir da 12ª de vida observamos já uma elevação da pressão arterial dos animais do grupo LP DNX. Isto possivelmente ocorra devido ao processo progressivo de reinervação renal. Em nosso trabalho, observamos que a elevação da pressão arterial ocorreu de forma gradual a partir da 12ª semana de vida correspondente a 4ª semana pós denervação.

Estudos recentes demonstraram, por imunofluorescência, que a densidade óptica dos nervos simpáticos em rins denervados foi reduzida em mais de 95% em relação ao rim contralateral inervado no primeiro dia após a denervação renal. Gradualmente, há um aumento do conteúdo de NPY (marcador de atividade eferente) nos nervos simpáticos renais chegando, na 4ª semana, a cerca de 40-50% do conteúdo quando comparado ao rim controle inervado, e a 100% entre 9 e 12 semanas após a denervação renal. Este mesmo estudo mostrou que os nervos simpáticos renais, tanto aferente quanto eferentes, reinervam o tecido renal de maneira similar ao padrão de inervação pré-denervação. Estudos mostram que existem diferenças entre espécies quanto ao tempo necessário para que haja a reinervação simpática pós-denervação, sendo semanas para ratos, meses em cães e de meses anos em humanos. (MULDER et al., 2013).

Como já havíamos dito, nossos animais apresentaram a elevação da pressão arterial de forma gradual a partir da 12ª semana de vida, sendo esta a 4ª semana pós denervação. Com estes dados, podemos inferir que reinervação renal está ocorrendo, aparentemente já apresentando participação ativa no controle da pressão arterial quando esta atinge cerca de 50% de reinervação. Estes, porém, são dados controversos uma vez que a hipertensão é dependente de muitos fatores, dentre elas a retenção de sódio e líquidos que não foram ainda significativos neste período de observação especificamente.

Nos GRD, principal porta de entrada do sinal nervoso enviado pelos rins aos SNC, não observamos diferenças entre as áreas nas diferentes subpopulações de neurônios, tanto pequenos quanto médios e grandes, porém, entre as imagens colhidas aleatoriamente dentro do gânglio por microscopia confocal, observação redução do número de neurônios pequenos no grupo LP em relação ao grupo controle. Os Neurônios pequenos, possuem fibras não-mielinizadas do tipo C, sendo as principais fibras relacionadas a atividade sensorial . Estes resultados também coincidem com aqueles previamente descritos por SCABORA et al., 2013, onde animais expostos à restrição proteica durante sua gestação apresentaram alterações de desenvolvimento do SNC com reduzido número de neurônios do tronco cerebral. Os resultados observados no presente estudo podem sugerir que uma possível redução da atividade sensorial como consequente aumento da atividade eferente possa decorrer desta redução dos número de neurônios pequenos.

Os resultados do presente estudo mostram expressiva redução da pressão arterial associada a redução da reabsorção tubular renal de sódio nos animais LP após a denervação renal completa bilateral. Simultaneamente, o estudo apresenta redução da imunorreatividade de Substancia P (SP) e CGRP nos neurônios dos GRD nos animais da prole de ratas programadas quando comparadas à prole controle. Adicionalmente, o estudo apresenta elevação na imunorreatividade de NK_1R nos corpos neuronais do GRD da prole LP.

Como em outros órgãos neurossensoriais, a atividade neural aferente renal é mediada pela SP e CGRP, sendo que estes neuropeptídeos estão co-localizados em parte expressiva das terminações nervosas da pelve renal. A expressão neuronal renal de CGRP é abundante se não mais intensa que a de substancia P na parede pélvica (LIU e BARAJAS 1993). Assim, aproximadamente 90% dos neurônios localizados entre T10–L3 nos gânglios da raiz dorsal, em ratos, são imunorreativos para SP e CGRP (KUO et al. 1984; SU et al. 1986).

Estudos prévios tem demonstrado que anormalidades foram observadas na atividade reflexa renorrenal em animais espontaneamente hipertensos com uma redução na secreção de substância P no efluente pélvico renal (KOPP et al. 1987; 1998). Além disso, a administração pélvica de substância P não eleva a ARNA nestes

animais de maneira semelhante ao observado em animais controles, caracterizando se uma importante disfunção do reflexo renorrenal (KOPP et al. 1998). Outros estudos em ratos normotensos demonstraram que a administração pélvica de substância P ou CGRP promovem respostas reflexas renorrenais similares e, que o tratamento com antagonistas destes receptores (capsaicina e h-CGRP8-37), atenuam esta resposta aferente renal ao aumento progressivo da pressão pélvica (GONTIJO et al. 1994; 1999; KOPP et al. 1993;1996). Estas observações, tomados em conjunto com o presente estudo, sugerem que a possível disfunção neurossensorial renal na prole LP possa estar associada com redução na produção neural destes neuropeptídeos em gânglios da raiz dorsal destes animais. Estas observações são importantes porque fornecem um novo quadro para a compreensão da etiologia das modificações funcionais renais observadas nos animais LP associadas com o desenvolvimento de hipertensão arterial.

Estudos em modelos de hipertensão de causa genética tem demonstrado uma redução na concentração plasmática de SP em resposta ao estresse, em comparação com indivíduos normotensos (FAULHABER et al. 1987), bem como vários estudos têm demonstrado um aumentado limiar à dor em homens e ratos hipertensos (GUASTI et al. 1995; IRVINE et al. 1997; SCHOBEL et al. 1996; SITSEN et al. 1997). Estes trabalhos sustentam a possibilidade que uma menor capacidade de resposta de receptores sensoriais renais na prole LP possa estar, em parte, relacionada a um defeito na síntese e/ou liberação de SP e/ou CGRP em terminações neurais renais. Os achados relativos à reduzida imunorreatividade ganglionar de SP e CGRP, vista aqui na prole de ratos LP suportam ainda a hipótese de que uma reduzida resposta aferente renal esteja relacionada à redução destas neurocininas.

Os NK1R são amplamente distribuídos em vários tecidos e órgãos. A alta marcação de NK1R no sistema nervoso sensorial (gânglios dorsais) sugere que este desempenha um papel importante no controle desta atividade neuronal. A presença de NK1R contribui para a ativação de receptores sensoriais renais em ratos normotensos (KOPP et al. 1991a; 1991b; 1993) . Estudos prévios tem demonstrado que CGRP regula a expressão de NK1R na medula espinhal, (SEYBOLD et al. 2003) e retarda o metabolismo de SP (GONTIJO et al. 1999), e desta forma aumenta a quantidade de SP disponível para estimulação de receptores de SP. Observações em vasos têm

demonstrado redução nos níveis da substância P nas terminações axonais de indivíduos hipertensos (EGASHIRA et al. 1995; HUANG et al. 1995; PANZA et al. 1994). No presente trabalho, a maior imunomarcação ganglionar de NK1R nos neurônios sensoriais bem como a elevada concentração pélvica de CGRP podem corresponder à tentativa de "*upregulation"* de uma alterada atividade neural sensorial nos animais da prole LP. No entanto, o presente estudo não exclui a possibilidade de que uma presumível redução da atividade sensorial renal em LP possa estar relacionada a defeito nos mecanismos de acoplamento do receptor de membrana de e seu agonista, substância P.

Nossos resultados também não podem afastar a possibilidade, a despeito da elevada imunomarcação de NK1R nos gânglios dorsais, de que possíveis alterações neurais no rim da prole LP resultem de um defeito de translocação destes receptores do citosol para a membrana neuronal (SOLOMON et a. 1999). Uma disfunção nesta translocação de NK1R associada ao decréscimo na produção de SP e CGRP nos GRD em ratos LP, poderia causar um aumento da retenção renal de sal e água, contribuindo para o desenvolvimento de hipertensão arterial nestes animais programados (MESQUITA et. al., 2011; LIMA et. al., 2012; SCABORA et al., 2013). Embora o presente estudo tenha demonstrado que a imunorreatividade de NK1R esteja aumentada nos GRD em LP comparado ao grupo NP, pouco conhecemos sobre as moléculas de sinalização e vias que são ativadas em função da ativação de receptores NK1. Uma vez que a região promotora que respondem a ativação de receptores NK1 envolve vias mediadas pelo cAMP (CRE), nós aventamos a hipótese que a redução dos níveis neuronais de SP e CGRP nos GRD possam regular a expressão de receptores NK1 via fatores de transcrição relacionados a cAMP (SEYBOLD et al. 2003).

Substância P e CGRP são potentes neuropeptídeos vasodilatadores cujo sitio de síntese são predominantemente os GRD. Os níveis de mRNA para CGRP estão elevados nos GRD e a expressão junto ao conteúdo de CGRP é elevado na medula espinhal em ratos hipertensos DOCA-Sal (SUPOWIT et al. 1993; 1995; 1997). Os corpos celulares dos gânglios da raiz dorsal sintetizam CGRP e exportam este neuropeptídeo para terminações axonais, vasos sanguíneos e centralmente para

medula espinhal. Estes sítios estão envolvidos na sua maioria na regulação da pressão arterial. O aumento da síntese deste potente vasodilatador ocorre compensatoriamente, em resposta a elevação pressórica. Entretanto, não se conhece se a produção neuronal de CGRP é elevada simplesmente pela elevação pressórica ou por modificações de outros parâmetros. No presente estudo a expressiva redução dos níveis de SP e CGRP nos GRD a despeito da elevação pressórica no grupo LP sugere que a elevação da pressão arterial, isoladamente, não contribui para modificações neuronais do conteúdo destes neuromoduladores. Uma vez que neste estudo não houve a determinação da expressão de mRNA para SP e CGRP nos GRD, nós não podemos excluir a possibilidade que uma elevação da pressão arterial module os níveis destes neuropeptídeos.

Os dados sobre os níveis circulantes de SP e CGRP são controversos em modelos experimentais de hipertensão arterial e em hipertensos humanos, com resultados que mostram aumento (MATSUDA et al. 1993), ausência de modificações (SCHIFTER et al. 1991), ou redução dos níveis. (EDVINSSON et al. 1989) Como descrito acima, tendo em conta os resultados do presente estudo, estes diferentes achados podem ser consequência de disfunção do transporte axonal ou um transporte assimétrico destes neuropeptídeos entre as terminações sensoriais centrais e periféricas como observado em modelos de diabetes mellitus experimental (TOMLINSON et al. 1988).

Adicionalmente, os níveis teciduais e plasmáticos de neurotrofinas são significativamente elevados em modelos de hipertensão arterial experimental. Estes elevados níveis estão relacionados à sobrevivência, diferenciação e ramificação da inervação simpática visceral (ZHANG et al. 2001). Dentre estas neurotrofinas encontra-se o fator de crescimento do nervo (NGF) que atua através da via tirosina quinase (trkA) no controle da síntese de diversos neuropeptídeos dentre os quais CGRP. Assim, a presença de NGF é responsável pela manutenção do fenótipo de populações neurossensoriais, pelo menos em parte, pela regulação da expressão de SP e CGRP (SNIDER et al. 1998)

Trabalhos recentes sugerem que os fenótipos de neurônios do GRD e neurônios nociceptivos do corno dorsal da medula espinhal são determinados por conjuntos distintos de fatores de transcrição (ZHANG et al. 2006). A especificação desses neurônios inclui a distribuição de controle e redistribuição de canais íons, receptores e suas moléculas regulatórias. A expressão superficial induzida por estímulo de receptores e canais iónicos aparentemente funciona como mecanismo que modula rapidamente a sensibilidade neuronal em resposta a estímulos nociceptivos. Entretanto, esta regulação induzida por estímulo de receptores e canais na dinâmica das interações entre as proteínas da membrana e na transmissão de sinais continua a ser investigado. Portanto, alterações crônicas de neuromodulação podem levar à reprogramação de fenótipos de neurônios no circuito e distorcer as características de estímulo-resposta normais do circuito.

Em conclusão, os resultados do presente estudo sugerem que anormalidades na ativação da atividade sensorial neural nos rins nos animais da prole LP, e consequentemente, hiperatividade eferente simpática, possam estar relacionadas a modificações na expressão de neuropeptídios nos neurônios do DRG. Nossos achados também evidenciam que a expressão de receptores NK1 possa ser controlada pelos níveis de neuropeptídios no SNC. Nós especulamos ainda que uma provável resposta inapropriada da atividade sensorial aferente renal nos animais LP possa estar relacionada a mecanismos que controlam a síntese de CGRP e substancia P e independem da elevação pressórica. Estas anormalidades poderiam contribuir para a retenção tubular renal de sódio e a elevação da pressão arterial nos animais programados.

5.1 Considerações finais.

A programação fetal é um conceito muito bem estabelecido no qual o principal marcador neonatal é o baixo peso ao nascer. Em modelos experimentais a manipulação da dieta materna durante a gestação é um dos principais métodos de estudo para se entender os mecanismos que levam a esta programação. Nossos animais apresentaram baixo peso ao nascimento com o desenvolvimento posterior de hipertensão, além de alterações no padrão de excreção de sódio, a qual pôde ser atenuada através da denervação renal bilateral mostrando a importância da inervação renal na manipulação dos eletrólitos pelos rins e na contribuição desta para o desenvolvimento do processo hipertensivo.

Supomos que em nosso modelo de restrição proteica gestacional a prole de ratos machos apresentam alterações na atividade simpática e, de acordo com a teoria do reflexo renorenal, nestes há um desequilíbrio entre atividade aferente e eferente contribuindo para maior liberação renal de catecolaminas, promovendo maior retenção tubular de sódio particularmente, pelos túbulos distais, aumento na liberação de renina pela mácula densa e vasoconstrição das arteríolas renais contribuindo para o processo hipertensivo neste modelo animal. Com o processo de denervação, retiramos este estímulo eferente, ou seja, menor estimulo adrenérgico sobre o rim o que contribuiu para redução na reabsorção de sódio, liberação de renina, reduzindo a pressão arterial nestes animais programados.

Através destes resultados podemos assumir que:

1. A Denervação renal bilateral na 8ª semana de vida, promoveu redução da pressão arterial sistólica e aumento da excreção de sódio na prole de ratas submetidas a restrição proteica gestacional. Observado na 10ª semana de vida.

2. Não foi observado alteração nos tamanhos dos neurônios, nem na quantidade total de neurônios observados na estereologia, porém, nas amostragens obtidas por microscopia confocal, observou-se redução no número de neurônios pequenos no gânglio da raiz dorsal (T13) na prole de ratas submetidas a restrição proteica gestacional.

3. Houve redução da imunorreatividade Substância P e CGRP no gânglio da raiz dorsal (T13) da prole de ratas submetidas a restrição proteica gestacional.

4. Houve aumento da imunorreatividade do receptor 1 neurocinina no gânglio da raiz dorsal (T13) da prole de ratas submetidas a restrição proteica gestacional.

5. Houve aumento da imunorreatividade CGRP na parede pélvica renal sem alterações significativas na quantidade de SP

CONCLUSÃO

6 CONCLUSÃO:

A prole de ratas submetidas à restrição proteica durante a gestação, apresenta alteração da função tubular renal (redução da excreção urinária de sódio), que pode contribuir para o desenvolvimento e/ou manutenção da hipertensão arterial nestes animais. Ao mesmo tempo o estudo demonstrou que a denervação renal bilateral atenua significativamente, a retenção renal de sódio e a elevação pressórica observada nesta prole programada. O estudo também evidenciou que ocorrem reduções no conteúdo de substância P, CGRP e aumento dos receptores-1 para neuroquinina nos gânglios da raiz dorsal associadas a elevação de CGRP na pelve renal, sugerindo modificações da resposta neurossensorial renal na prole LP. Juntos estes achados podem sugerir o envolvimento neural renal na retenção de sódio e na elevação da pressão arterial observados neste modelo de programação fetal induzida pela restrição proteica durante toda a gestação

REFERÊNCIAS BIBLIOGRÁFICAS

7 REFERÊNCIAS BIBLIOGRÁFICAS

ASHTON N. Perinatal development and adult blood pressure. **Brazilian *Journal* of Medical and Biological Research**. 2000; 33: 731-40.

BARAJAS L, POWERS K, WANG P. Innervation of the renal cortical tubules: a quantitative study. **The American journal of physiology** 1984. 247; 50-60.

BARAJAS L, POWERS K. Monoaminergic innervation of the rat kidney: a quantitative study. **Am J Physiol**. 1990. 259: 503-11.

BARKER DJP GLUCKMAN PD, GODFREY KM, HARDING JE, OWENS JA, ROBINSON JS. Fetal nutrition and cardiovascular disease in adult life. Lancet . 1993; 341, 938–94.

BARKER DJP, WINTER PD, OSMOND C, MARGETTS B, SIMMONDS, SJ. Weight in infancy and death from ischaemic heart disease. **Lancet**. 1989. 577-580.

BARKER DJP. In utero programming of chronic disease. **Clin Sci** (Lond). 1998; 95:115-128.

BEITINS IZ, BAYARD F, ANCES IG, KOWARSKI A, MIGEON CJ: The metabolic clearance rate, blood production, interconversion and transplacental passage of cortisol and cortisone in pregnancy near term. **Pediatr Res.** 1973; 7: 509–519.

BENEDIKTSSON R, BRENNAND J, TIBI L, CALDER AA, SECKL JR, EDWARDS CRW. Fetal osteocalcin levels are related to placental 11b-hydroxysteroid dehydrogenase activity. **Clin Endocrinol**. 1995; 42: 551–555.

BENEDIKTSSON R, CALDER AA, EDWARDS CRW, SECKL JR. Placental 11b-hydroxysteroid dehydrogenase: a key regulator of fetal glucocorticoid exposure **Clin Endocrinol**. 1997; 46:161–166.

BENEDIKTSSON R, LINDSAY R, NOBLE J, SECKL JR, EDWARDS CRW. Glucocorticoid exposure in utero: A new model for adult hypertension. **Lancet**. 1993; 341:339–341.

BIANCHI G.; FOX U, DIFRANCESCO GF GIOVANNETI AM, BAGETTI D. Blood pressure changes produced by kidney cross-transplantation between spontaneously hypertensive rats (SHR) and normotensive rats (NR). **Clinical Science and Molecular Medicine**, 1974; 47: 435.

BOER PA, GONTIJO JA. Nuclear localization of SP, NK1R and CGRP in the dorsal root ganglion cells. **Cellular and Molecular Neurobiology**. 2006. 26; 191-207.

BOER PA, UENO M, SANTANA J, SAAD M, GONTIJO JA. Expression and localization of NK(1)R, substance P and CGRP are altered in dorsal root ganglia neurons of spontaneously hypertensive rats (SHR). **Molecular Brain Research**. 2005. 29; 138; 35-44

BRADLEY T, HJEMDAHL P. Influence of afferent renal nerve activity on contralateral renal overflow of noradrenaline and dopamine to plasma in the dog. **Acta Physiol Scand**. 1986.128(1):119-20.

CHALLIS JR, SLOBODA D, MATTHEWS SG, HOLLOWAY A, ALFAIDY N, PATEL FA, WHITTLE W, FRASER M, MOSS TJ, NEWNHAM J. The fetal placental hypothalamic-pituitary-adrenal (HPA) axis, parturition and post natal health. **Mol Cell Endocrinol.** 2001. 20;185 (1-2):135-44.

COLE TJ, BLENDY JA, MONAGHAN AP, SCHMID W, AGUZZI A, SCHUTZ G: Molecular genetic analysis of glucocorticoid signaling during mouse development. **Steroids**. 1995; 60:93–96.

COLINDRES RE, SPIELMAN WS, MOSS NG, HARRINGTON WW, GOTTSCHALK CW.Functional evidence for renorenal reflexes in the rat. **Am J Physiol**. 1980; 239(3):F265-70.

CONVERSE RL Jr, JACOBSEN TN, TOTO RD, JOST CM, COSENTINO F, FOUAD-TARAZI F, VICTOR RG. Sympathetic overactivity in patients with chronic renal failure**. N Engl J Med**. 1992. 327: 1912–1918.

CROWLEY SD, GURLEY SB, OLIVERIO MI, PAZMINO AK, GRIFFITHS R, FLANNERY PJ, et al. Distinct roles for the kidney and systemic tissues in blood pressure regulation by the renin-angiotensin system. **J Clin Invest** 2005; 115: 1092-1099.

DAVE-SHARMA S, WILSON RC, HARBISON MD, NEWFIELD R, AZAR M, KROZOWSKI ZS, FUNDER JW, SHACKLETON CHL, BRADLOW HL, WEI JQ, HERTECANT J, MORAN A, NEIBERGER RE, BALFE JW, FAT-TAH A, DANEMAN D, AKKURT HI, DESANTIS C, NEW MI: Extensive personal experience: Examination of genotype and phenotype relationships in 14 patients with apparent mineralocorticoid excess. **J Clin Endocrinol Metab**. 1998; 83:2244–2254.

DEVANE CL. Substance P: a new era, a new role. **Pharmacotherapy**. 2001 21(9):1061-9.

DIAZ R, FUXE K, OGREN SO: Prenatal corticosterone treatment induces long-term changes in spontaneous and apomorphine-mediated motor activity in male and female rats. **Neuroscience.** 1997; 81:129–140.

DIBONA GF, JOHNS EJ.A study of the role of renal nerves in the renal responses to 60 degree head-up tilt in the anaesthetized dog. **J Physiol**. 1980. 299:117-26.

DIBONA GF, KOPP UC. Neural control of renal function. **Physiol Rev** 1997. 77: 75–197.

DIBONA GF, SAWIN LL. Renal nerves in renal adaptation to dietary sodium restriction. **Am J Physiol Renal Fluid Electrolyte Physiol** 1983. 245: F322–F328.

DIBONA, G.F. Sympathetic neural control of the kidney in hypertension. **Hypertension**, 1992, 19(suppl I):28-35.

DONOVAN MK, WYSS JM, WINTERNITZ SR. Localization of renal sensory neurons using the florescent dye techinique. Brain Res, 1983; 259: 119-122.

EDVINSSON L, EKMAN R, THULIN T. Reduced levels of calcitonin gene-related peptide (CGRP) but not substance P during and after treatment of severe hypertension in man **J. Hum. Hypertens.** 1989. 3; 267–270

EDWARDS CRW Dysfunction of the placental glucocorticoid barrier: a link between the foetal environment and adult hypertension? **Lancet** 1993. 341, 355–357.

EDWARDS LJ, COULTER CL, SYMONDS ME, MCMILLEN IC. Pre-natal undernutrition, glucocorticoids and the programming of adult hypertension. ***Clinical* and Experimental *Pharmacology* and *Physiology*.** 2001; 28: 938-41.

EGASHIRA K, SUZUKi S, HIROOKA Y, Kai K, SUGIMACHI M, IMAIZUMI T, TAKESHITA A. Impaired endothelium-dependent vasodilation of large epicardial and resistance coronary arteries in patients with essential hypertension **Hypertension,** 1995. 25; 201–206.

ESLER MD, KRUM H, SOBOTKA PA, SCHLAICH MP, SCHMIEDER RE, BÖHM M. Renal sympathetic denervation in patients with treatment-resistant hypertension (The Symplicity HTN-2 Trial): a randomized controlled trial. **Lancet**. 2010. 376: 1903–1909.

FAULHABER HD, OEHME P, BAUMANN R, ENDERLEIN J, RATHSACK R, ROSTOCK RE, NAUMANN E. Substance P in human essential hypertension. **J. Cardiovasc. Pharmacol**. 1987. 10; 172–176

FERGUSON M, BELL C. Ultrastructural localization and characterization of sensory nerves in the rat kidney. **J Comp Neurol**, 1988; 247: 9-16,

FORSDAHL A. Are poor living conditions in childhood and adolescence an important risk factor for arteriosclerotic heart disease? **British Journal of Preventive and Social Medicine**, 1977, 31, 91-95

FREY BAJ, GRISK O, BANDELOW N, WUSSOW S, BIE P, RETTIG R. Sodium homeostasis in transplanted rats with a spontaneously hypertensive rat kidney. **American Journal of Physiology**, 2000; 279: R1099-R1104.

GIBSON SJ, POLAK JM, BLOOM SR, SABATE IM, MULDERRY PM, GHATEI MA, MCGREGOR GP, MORRISON JF, KELLY JS, EVANS RM, Calcitonin gene-related peptide immunoreactivity in the spinal cord of man and of eight other species. **J Neurosci**. 1984 4:3101-3111.

GLUCKMAN PD, HANSON MA. The developmental origins of the metabolic syndrome. **Endocrinology and Metabolism**. 2004; 15, 183-187

GONTIJO JAR, KOPP UC. Renal sensory receptor activation by calcitonin gene-related peptide. **Hipertension.** 1994; 23: 1063-1067.

GONTIJO JAR, SMITH LA, KOPP UC. CGRP activates renal pelvic substance P receptors by retarding substance P metabolism. Hipertension. 1999; 33: 493-498.

GUASTI L, CATTANEO R, RINALDI O, ROSSI MG, BIANCHI L, GAUDIO G, GRANDI AM, GORINI G, VENCO A. Twenty-four-hour noninvasive blood pressure monitoring and pain perception **Hypertension.** 1995. 25; 1301–1305

GUIDI E, MENGHETTI D, MILANI S, MONTAGNINO G, PALAZZI P, BIANCHI G. Hypertension may be transplanted with the kidney in humans: a long-term historical prospective follow-up of recipients grafted with kidneys coming from donors with or without hypertension in their families. **J Am Soc Nephrol** 1996; 7: 1131-1138.

GUYENET PG The sympathetic control of blood pressure. **Nature Reviews Neuroscience.** 2006. 7, 335-346.

HALES CN, BARKER DJP, CLARK PMS, COX LJ, FALL C, OSMOND C, WINTER PD. Fetal and infant growth and impaired glucose tolerance at age 64 **BMJ** 1991;303:1019-1022.

HANSON MA, GLUCKMAN PD. Developmental origins of health and disease: new insights. Basic Clin Pharmacol Toxicol. 2008; 102:90-93.

HARRIS A, SECKL JR. Glucocorticoids, prenatal stress and the programming of disease. **Hormones and Behavior.** 2011; 59; 279–289.

HOPPE CC, EVANS RG, BERTRAM JF, MORITZ KM. Effects of dietary protein restriction on nephron number in the mouse. **Am J Physiol Regul Integr**. 2007;292 (5): 1768-74.

HUANG A, KOLLER A. Both nitric oxide and prostaglandin-mediated responses are impaired in skeletal arterioles of hypertensive rats **J. Hypertens**. 1996.14; 887–895.

IRVINE RJ, WHITE JM. The effects of central and peripheral angiotensin on hypertension and nociception in rats. **Pharmacol. Biochem. Behav**. 1997. 57; 37–41.

JANSSON N, PETTERSSON J, HAAFIZ A, ERICSSON A, PALMBERG I, TRANBERG M, GANAPATHY V, POWELL TL, JANSSON T Down-regulation of placental transport of amino acids precedes the development of intrauterine growth restriction in rats fed a low protein diet. **J Physiol.** 2006. 576; 935–946.

JUDY WV, FARREL SK. Arterial baroreceptor reflex control of sympathetic nerve activity in the spontaneously hypertensive rat. **Hypertension**, 1: 605-614, 1979.

KOEPKE JP,DIBONA GF, Functions of the renal nerves. **Physiologist**. 28 1985:47-52.

KOPP UC, SMITH L A, Inhibitory renorenal reflexes: a role for substance P or other capsaicin-sensitive neurons. **The American journal of physiology**. 1991a; 260: 232-239.

KOPP UC, CICHA MZ, FARLEY DM, SMITH LA, DIXON BS. Renal Substance P-Containing neurons and supstance P receptors impaired in hypertension. Hypertension, 1998. 31:815-822.

KOPP UC, CICHA MZ, SMITH LA, MULDER J, HÖKFELT T. Renal sympathetic nerve activity modulates afferent renal nerve activity by PGE2-dependent activation of α 1- and α 2-adrenoceptors on renal sensory nerve fibers. **Am J Physiol Regul Integr Comp Physiol,** 2007 293: 1561–1572,

KOPP UC, CICHA MZ, SMITH LA, RUOHONEN S, SCHEININ M, FRITZ HOKFELT T. Dietary sodium modulates the interaction between efferent and afferent renal nerve activity by altering activation of α 2-adrenoceptors on renal sensory nerves. **Am J Physiol Regul Integr Comp Physiol**, 2011b 300: 298–310,

KOPP UC, CICHA MZ, SMITH LA. Impaired interaction between efferent and afferent renal nerve activity in SHR involves increased activation of α2-adrenoceptors. **Hypertensio**n. 2011a, 57: 640–647.

KOPP UC, OLSON LA, DIBONA GF. Renorenal reflex responses to mechano- and chemoreceptor stimulation in the dog and rat. **Am J Physiol**, 1984 246:F67–F77.

KOPP UC, SMITH LA, DIBONA GF. Impaired renorenal reflexes in spontaneously hypertensive rats. **Hypertension**,1987. 9:69–75.

KOPP UC, SMITH LA. Inhibitory renorenal reflexes: a role for renal prostaglandins in activation of renal sensory receptors. **Am J Physiol**, 1991b 261:1513–1521.

KREULEN DL, PETERS S. Noncholinergic Transmission In A Sympathetic-Ganglion Of The Guinea-Pig Elicited By Colon Distension. **Journal Of Physiology-London.** 1986; 374: 315-334.

KRUM H, BARMAN N, SCHLAICH M, SOBOTKA P, ESLER M, MAHFOUD F, BOHM M, DUNLAP M. Catheter-based renal sympathetic denervation for resistant hypertension. Durability of blood pressure reduction out to 24 months. **Hypertension.** 2011. 57: 911–917.

LANGLEY-EVANS SC, PHILIPS G, BENEDIKTSSON R, GARDNER D, EDWARDS CRW, JACKSON AA, SECKL JR. Maternal dietary protein restriction, placental glucocorticoid metabolism and the program of hypertension. **Placenta**. 1996a; 17:169–172.

LANGLEY-EVANS SC, WELHAM SJ, SHERMAN RC, JACKSON AA. Weanling rats exposed to maternal low-protein diets during discrete periods of gestation exhibit differing severity of hypertension. **Clin Sci (Lond).** 1996b; 91:607-615.

LANGLEY-EVANS SC, WELHAM SJM, JACKSON AA. Fetal exposure to a maternal low protein diet impairs nephrogenesis and promotes hypertension in the rat. **Life science**. 1999. 64; 11; 965-974.

LARSON AA, SUN X. Amino terminus of substance P potentiates kainic acid-induced activity in the mouse spinal cord. **J Neurosci**. 1992. 12; 4905-4910.

LAW CM, BARKER DJP, BULL AR, OSMOND C. Maternal and fetal influences on blood pressure. **Arch Dis Child**. 1991. 61: 1291-1295.

LE GREVES P, NYBERG F, TERENIUS L, HÖKFELT T. Calcitonin gene-related peptide is a potent inhibitor of substance P degradation **European Journal of Pharmacology**.1895, 24; 309–311.

LIMA MC, SCABORA JE, LOPES A, MESQUITA FF, TORRES D, BOER PA, GONTIJO JA. Early changes of hypothalamic angiotensin II receptors expression in gestational protein-restricted offspring: effect on water intake, blood pressure and renal sodium handling. **J Renin Angiotensin Aldosterone Syst**. 2012.

LINDSAY RS, LINDSAY RM, EDWARDS CRW, SECKL JR.Inhibition of 11β-Hydroxysteroid Dehydrogenase in Pregnant Rats and the Programming of Blood Pressure in the Offspring. **Hypertension**.1996; 27: 1200-1204.

LIU L, BAJARAS L .The rat nerves during development, **Anat, Embriol**. 1993; 188: 345-361.

LUCAS A. Programming by early nutrition in man. In: Bock, G.R. and Whelan, J. (Eds) *The Childhood Environmental and Adult Disease*. John Wiley e Sons, Chichester, UK, 1991. pp. 38-55.

LUYCKX VA, BRENNER BM. Low birth weight, nephron number, and kidney disease. **Kidney Int Suppl**. 2005; (97): 68-77.

MAO C, SHI L, XU F, ZHANG L, XU Z. Development of fetal brain renin-angiotensin system and hypertension programmed in fetal origins. **Prog Neurobiol**. 2009. 87(4):252-63.

MASUDA A, SHIMAMOTO K, MORI Y, NAKAGAWA N, URA N, IIMURA O. Plasma calcitonin gene-related peptide levels in patients with various hypertensive diseases. **J. Hypertens.** 1992. 10 ; 1499–1504.

MATTHEWS, S.G.,. Antenatal glucocorticoids and programming of the developing CNS. **Pediatr. Res**. 2000; 47, 291–300.

MCMILLEN IC, ROBINSON JS. Developmental origins of the metabolic syndrome: prediction, plasticity, and programming. Physiol Rev. 2005; 85:571-633.

MCMULLEN S, GARDNER DS, LANGLEY-EVANS SC. Prenatal programming of angiotensin II type 2 receptor expression in the rat. **British Journal of Nutrition**. 2004;91:133–140.

MCMULLEN S, LANGLEY-EVANS SC. Maternal low-protein diet in rat pregnancy programs blood pressure through sex-specific mechanisms. **American Journal of Physiology**. 2005; 288:85–90.

MEANEY M, SZYF M, SECKL JR. Epigenetic mechanisms of perinatal programming of hypothalamic–pituitary–adrenal function and health. **Mol. Med**. 2007; 13, 269–277.

MENDELSOHN ME. In hypertension, the kidney is not always the heart of the matter. **J Clin Invest** 2005; 115: 840-844.

MESQUITA FF, GONTIJO JAR, BOER PA. Expression of renin-angiotensin system signalling compounds in maternal protein-restricted rats: effect on renal sodium excretion and blood pressure. **Nephrol Dial Transplant.** 2010b) 25: 380–388.

MESQUITA FF, GONTIJO JAR, BOER PA. Maternal undernutrition and the offspring kidney: from fetal to adult life. **Brazilian Journal of Medical and Biological Research**. 2010a; 43: 1010-1018.

MULDER J1, HÖKFELT T, KNUEPFER MM, KOPP UC. Renal sensory and sympathetic nerves reinnervate the kidney in a similar time-dependent fashion after renal denervation in rats. **Am J Physiol Regul Integr Comp Physiol**. 201315;304(8):R675-82.

NAKAYA Y, KANEKO T, SHIGEMOTO R, NAKANISHI S, MIZUNO N. Immunohistochemical localization of substance-p receptor in the central-nervous-system of the adult-rat. **Journal Of Comparative Neurology.** 1994; 347: 249-274.

NISHINAKAMURA R, UCHIYAMA Y, SAKAGUCHI M, FUJIMURA S. Nephron progenitors in the metanephric mesenchyme. **Pediatr Nephrol**. 2011. 26(9):1463-1467.

OJEDA NB, GRIGORE D, ALEXANDER BT. Role of fetal programming in the development of hypertension. **Future Cardiol**. 2008. 4(2):163-74.

PANETH N, AHMED F, STEIN AD. Early nutritional origins of hypertension: a hypothesis still lacking support. **Journal of Hypertension**. 1996. 14 (Suppl 5): S121-S129.

PANZA JA, CASINO PR, KILCOYNE CM, QUYYUMI AA. Impaired endothelium-dependent vasodilation in patients with essential hypertension: evidence that the abnormality is not at the muscarinic receptor level. **J. Am. Coll. Cardiol**. 1994. 23; 1610–1616.

PERSSON, E.; JANSSON, T. Low birth weight is associated with elevated adult blood pressure in the chronically catheterized guinea-pig. **Acta Physiologica Scandinavica**. 1992; 145(2): 195-196.

RECORDATI G, GENOVESI S, CERATI D, di CINTIO R Reno-renal and reno-adrenal reflexes in the rat. **Clin Sci (Lond).** 1980. 59 Suppl 6:323-325.

RECORDATI GM, MOSS NG, GENOVESI S, ROGENES PR Renal receptors in the rat sensitive to chemical alterations of their environment. **Circ Res**. 1980. 46(3):395-405.

RECORDATI GM, MOSS NG, WASELKOV L. Renal chemoreceptors in the rat.**Circ Res**. 1978.43(4):534-43.

RETTIG R, FOLBERTH C, STAUSS H, KOPF D, WALDHERR R, UNGER T. Role of the kidney in primary hypertension: a renal transplantation study in rats. **Am J Physiol** 1990; 258: F606-F611.

REYNOLDS, RM. Glucocorticoid excess and the developmental origins of disease: two decades of testing the hypothesis–2012 Curt Richter Award Winner. **Psychoneuroendocrinology** , 2013; 38, 1–11.

ROSE G. Familial patterns in ischaemic heart disease. **British Journal of Preventive and Social Medicine**. 1964; 18:75-80.

ROSTAND SG. Oligonephronia, primary hypertension and renal disease: 'is the child father to the man?'. **Nephrol Dial Transplant**. 2003; 18(8):1434-1438.

SANDMAN CA, DAVIS EP, BUSS C, GLYNN LM. Prenatal programming of human neurological function. **International Journal of Peptides** Vol 2011.

SCABORA JE, DE LIMA MC, LOPES A, DE LIMA IP, MESQUITA FF, BRÁZ TORRES D, BOER PA, GONTIJO JA. Impact of taurine supplementation on blood pressure in gestational protein-restricted offspring: Effect on the medial solitary tract nucleus cell numbers, angiotensin receptors, and renal sodium handling. **J Renin Angiotensin Aldosterone Syst**. 2013.

SCHIFTER S, KRUSELL LR, SEHESTED J. Normal serum levels of calcitonin gene-related peptide (CGRP) in mild to moderate essential hypertension. **Am. J. Hypertens**. 1991. 4; 565–569.

SCHOBEL HP, RINGKAMP M, BEHRMANN A, FORSTER C, SCHMIEDER RE, HANDWERKER HO**.** Hemodynamic and sympathetic nerve responses to painful stimuli in normotensive and borderline hypertensive subjects. **Pain**. 1996. 66 ; 117–124

SECKL JR, HOLMES MC. Mechanisms of Disease: glucocorticoids, their placental metabolism and fetal 'programming' of adult pathophysiology. **Endocrinology e Metabolism**. 2007; v.3; n.6; 479-489.

SECKL, JR. Glucocorticoids, feto-placental 11 beta-hydroxysteroid dehydrogenase type 2, and the early life origins of adult disease. **2nd International Symposium on Molecular Steroidogenesis**. 1997; 62(1):89-94

SEYBOLD VS, MCCARSON KE, MERMELSTEIN PG, GROTH RD, ABRAHAMS LG. Calcitonin gene-related peptide regulates expression of neurokinin1 receptors by rat spinal neurons **J. Neurosci**. 2003. 23 ; 1816–1824

SITSEN JMA, DE JONG W. Hypoalgesia in genetically hypertensive rats is absent in rats with experimental hypertension **Hypertension.** 1983. 5; 185–190

SKOFITSCH G, JACOBOWITZ DM Quantitative distribution of calcitonin gene-related peptide in the rat central nervous system. **Peptides**. 1985 6:1069-1073.

SNIDER WD, MCMAHON SB. Tackling pain at the source: new ideas about nociceptors**. Neuron**. 1998. 20; 629–632

SOLOMON SG, LLEWELLYN-SMITH IJ, MINSON JB, ARNOLDA LF, CHALMERS JP, PILOWSKY PM. Neurokinin-1 receptors and spinal cord control of blood pressure in spontaneously hypertensive rats**. Brain Res**. 1999. 815; 116–120

SUPOWIT SC, CHRISTENSEN MD, WESTLUND JN, HALLMAN DM, DIPETTE DJ. Dexamethasone and activators of the protein kinase A and C signal transduction pathways regulate neuronal calcitonin gene-related peptide expression and release. **Brain Res**. 1994. 686; 77–86.

SUPOWIT SC, GURURAJ A, RAMANA CV, WESTLUND KN, DIPETTE DJ. Enhanced neuronal expression of calcitonin gene-related peptide in mineralocorticoid-salt hypertension **Hypertension**, 1995. 25; 1333–1338.

SUPOWIT SC, RAMANA CV, WESTLUND KN, DIPETTE DJ**.** Calcitonin gene-related peptide gene expression in the spontaneously hypertensive rat**. Hypertension**, 1993. 21; 1010–1014.

SUPOWIT SC, ZHAO H, HALLMAN DM, DIPETTE DJ. Calcitonin gene-related peptide is a depressor of deoxycorticosterone-salt hypertension in the rat **Hypertension.** 1997. 29; 945–950.

TAKAMI K, Kawai Y, SHIOSAKA S, LEE Y, GIRGIS S, HILLYARD CJ, MACINTYRE I, EMSON PC, TOHYAMA M. Immunohistochemical evidence for the coexistence of calcitonin gene-related peptide- and choline acetyltransferase-like immunoreactivity in neurons of the rat hypoglossal, facial and ambiguus nuclei. **Brain Res**. 1985. 4;328 (2):386-389.

TOMLINSON DR, ROBINSON JP, WILLARS GB, KEEN P. Deficient axonal transport of substance P in streptozocin-induced diabetic rats. Effects of sorbinil and insulin **Diabetes**. 1988. 37; 488–493.

VACCARI B, MESQUITA FF, GONTIJO JA, BOER, PA. Fetal kidney programming by severe food restriction: Effects on structure, hormonal receptor expression and urinary sodium excretion in rats. **Journal of the Renin-Angiotensin-Aldosterone System**. 2013, (0), 1-14.

WATKINS AJ, WILKINS A, CUNNINGHAM C. Low protein diet fed exclusively during mouse oocyte maturation leads to behavioural and cardiovascular abnormalities in offspring. J Physiol. 2008; 586, 2231–2244.

WEINSTOCK M, POLTYREV T, SCHORER-APELBAUM D, MEN D, MCCARTY R. Effect of prenatal stress on plasma corticosterone and catecholamines in response to footshock in rats. **Physiol Behav**. 1998. 15;64(4):439-44.

WEINSTOCK, M, The long-term behavioural consequences of prenatal stress. **Neurosci. Biobehav. Rev**. 2008; 32, 1073–1086

WEISS ML, CHOWDHURY SI. The renal afferent pathways in the rat: a pseudorabies virus study. **Brain Res**. 1998. 23;812 :227-41.

WELBERG LA, SECKL JR, HOLMES MC. Prenatal glucocorticoid programming of brain corticosteroid receptors and corticotrophin-releasing hormone: possible implications for behaviour. **Neuroscience**. 2001;104(1):71-9.

WOODS LL, INGELFINGER JR, NYENGAARD JR, RASCH R. Maternal protein restriction suppresses the newborn renin-angiotensin system and programs adult hypertension in rats. Pediatr Res. 2001; 49(4): 460-467.

WOODS LL, WEEKS DA, RASCH R. Programming of adult blood pressure by maternal protein restriction: role of nephrogenesis. **Kidney Int**. 2004; 65(4): 1339-1348.

WYSS JM, CARLSON SH. The role of the central nervous system in hypertension. **Curr Hypertens Rep**. 1999. 1(3): 246-253.

XAVIER, F.; MAGALHÃES, A.M.F.; GONTIJO, J.A.R. Effect of inhibition of nitric oxide synthase on blood pressure and renal sodium handling in renal denervated rats. **Brazilian Journal of Medical and Biological Research**, 33: 347-354, 2000.

ZANDI-NEJAD K, LUYCKX VA, BRENNER BM. Adult hypertension and kidney disease: the role of fetal programming. *Hypertension* 2006; 47: 502-508.

ZEMAN FJ. Effects of maternal protein restriction on the kidney of the newborn young of rats disease. Hormones and Behavior Volume 59, Issue 3, March 2011, Pages 279–289

ZHANG SH, RUSH RA. Neurotrophin 3 is increased in the spontaneously hypertensive rat. **J. Hypertens.** 2001. 19; 2251–2256.

ZHANG X, BAO L. The development and modulation of nociceptive circuitry. **Curr Opin Neurobiol**. 2006 16(4): 460-466.
