# Supplementary material for: Renal sodium handling and blood pressure changes in gestational protein-restricted offspring: Role of renal nerves and ganglia neurokinin expression
Source: PLoS One. 2017 Jun 20;12(6):e0179499. doi: 10.1371/journal.pone.0179499 (PMC5478103; doi:10.1371/journal.pone.0179499)
Supplement: S1 File — (DOCX) [file pone.0179499.s001.docx]

Campinas, Brazil May 2017

Dear Editor:

In response to your questions:

1. Please confirm if the Supporting Information thesis PDF file contains all the data used within this study in order to draw the conclusions of your submitted manuscript. Additionally, please confirm that this data will allow future. If this is not the case and you need to supply additional data as part of your minimal data set, please confirm this and upload your data as Supporting Information files or to a public repository.

*R.: I confirm that supporting Information in PDF version of the Master Custódio Thesis file contains all the data used within this study in order to draw the conclusions of your submitted manuscript. Additionally, I confirm that all data and PDF file will permit future access by public repository.*

2) Please confirm if permission is required to upload and re-publish the PDF version of the Master Custódio Thesis. Who owns the thesis and the PDF file? Please note, PLOS ONE applies the CC-BY license to all articles we publish. If permission is needed, we will need permission in writing that your thesis can be republished with PLOS under the CC-BY license. Please see the following link for more information: <http://journals.plos.org/plosone/s/licenses-and-copyright>

*R.: I confirm that permission to upload or reproduction of the PDF version of the Master Custódio Thesis is not required; it can accessed any time by site of Digital Library of State University of Campinas: (*[*http://www.bibliotecadigital.unicamp.br/document/?code=000932922&fd=y*](http://www.bibliotecadigital.unicamp.br/document/?code=000932922&fd=y)*) or by CAPES Thesis Bank: (*[*https://sucupira.capes.gov.br/sucupira/public/consultas/coleta/trabalhoConclusao/viewTrabalhoConclusao.jsf?popup=true&id_trabalho=1473651*](https://sucupira.capes.gov.br/sucupira/public/consultas/coleta/trabalhoConclusao/viewTrabalhoConclusao.jsf?popup=true&id_trabalho=1473651)*)*

3) Please confirm if the following updated Data Availability statement is accurate and acceptable to you:

*R.: Yes, I confirm that "All relevant data are within the paper and its Supporting Information files."*

Sincerely,

José Gontijo, MD

State University of Campinas, Campinas, SP

Brazil
